# From alkylating to shape-shifting G-quadruplex ligands: the RHAU peptide story

**DOI:** 10.1093/nar/gkag039

**Published:** 2026-01-29

**Authors:** Lessandro De Paepe, Simona Marzano, Camille Vesschemoet, Jussara Amato, Bruno Pagano, Enrico Cadoni, Annemieke Madder

**Affiliations:** Department of Organic and Macromolecular Chemistry, Organic and Biomimetic Chemistry Research Group, Ghent University, Krijgslaan 281, Building S4, B-9000 Gent, Belgium; Department of Pharmacy, University of Naples Federico II, via D. Montesano 49, 80131 Naples, Italy; Department of Organic and Macromolecular Chemistry, Organic and Biomimetic Chemistry Research Group, Ghent University, Krijgslaan 281, Building S4, B-9000 Gent, Belgium; Department of Pharmacy, University of Naples Federico II, via D. Montesano 49, 80131 Naples, Italy; Department of Pharmacy, University of Naples Federico II, via D. Montesano 49, 80131 Naples, Italy; Department of Organic and Macromolecular Chemistry, Organic and Biomimetic Chemistry Research Group, Ghent University, Krijgslaan 281, Building S4, B-9000 Gent, Belgium; Department of Organic and Macromolecular Chemistry, Organic and Biomimetic Chemistry Research Group, Ghent University, Krijgslaan 281, Building S4, B-9000 Gent, Belgium

## Abstract

G-quadruplexes (G4s) are non-canonical secondary nucleic acid structures with important biological implications in telomere elongation and gene expression. A large number of small molecules have been developed to bind and even covalently target these structures, enhancing the potency and duration of binding. Alternatively, peptide-based ligands have been studied and shown to offer several advantages, including high specificity, a modular design, and ease of synthesis. In this work, we describe a peptide-based methodology for covalent G4-targeting, based on the introduction of two photoactivatable moieties in a peptide derived from the RHAU helicase. Rational insertion of crosslinkers at different positions yielded nine different peptides, which were evaluated for their G4-stabilizing effect and alkylation potential. Moderate to high alkylation yields towards G4s were obtained. The G4 stabilizing potential drastically increased for N-terminal modifications of the RHAU18 peptide. This led to the design of a further series of peptides with varying N-terminal residues to gain insight in the stabilization potential of each single amino acid modification and provided a comprehensive study of the binding behaviour of modified RHAU peptides.

## Graphical Abstract

**Graphical Abstract:**
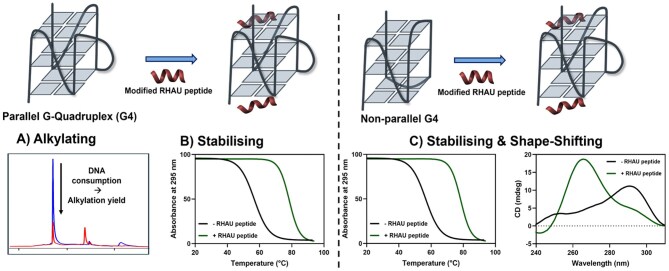

## Introduction

**Figure 1.**
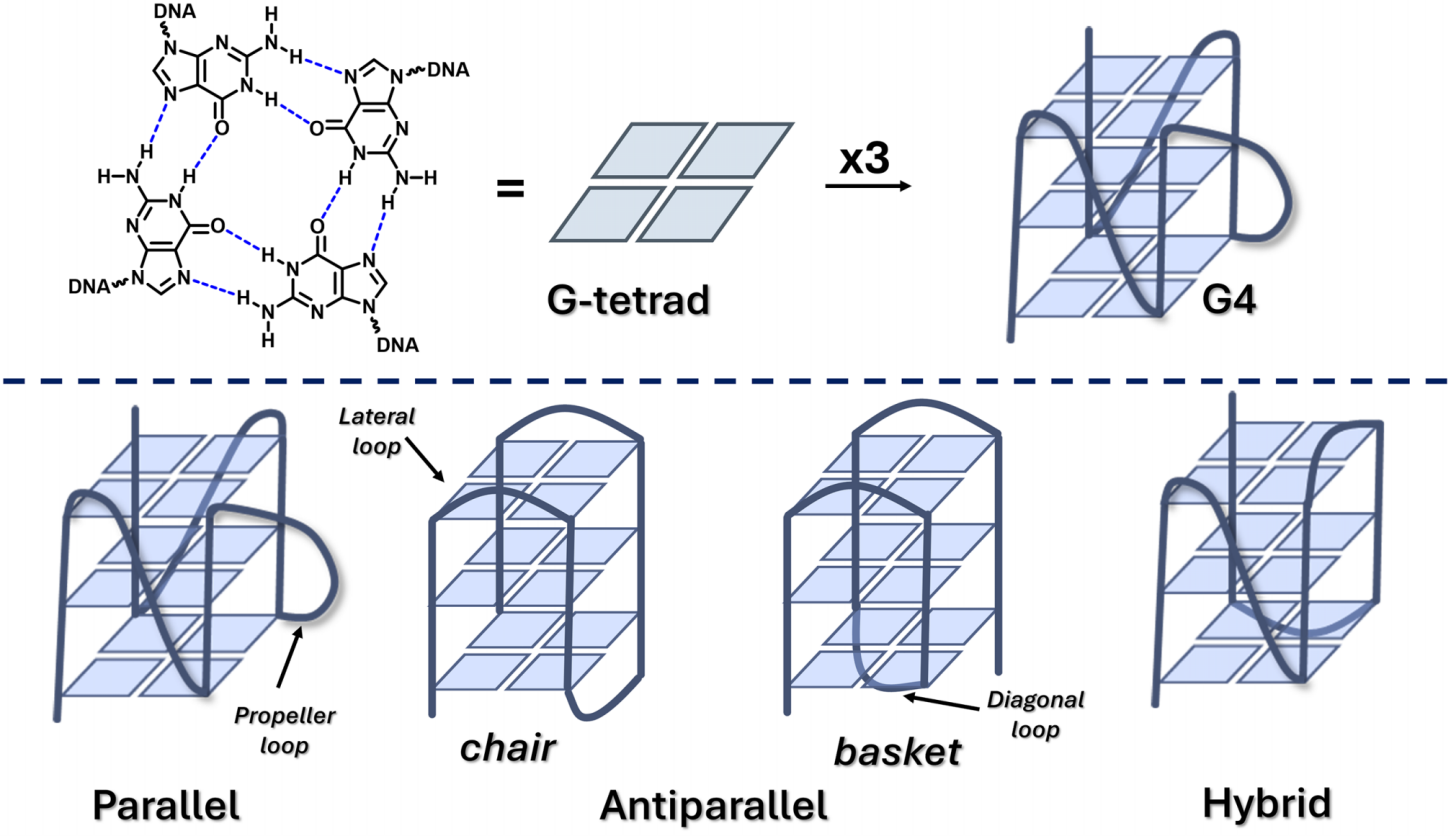
Schematic representation of G-quadruplex formation (top) and topological diversity (bottom).

Guanosine-rich nucleic acid sequences have the intrinsic propensity to fold into alternative secondary structures, known as G-quadruplexes (G4s). These G4s are composed of two or more stacking G-tetrads, planar structures formed by four guanine residues, stabilized by Hoogsteen hydrogen bonds and cations (including, but not exclusively, K^+^ or Na^+^) [1]. While the G-tetrad structure is a common feature, G4s are highly polymorphic and dynamic structures that can adopt various topological conformations (parallel, anti-parallel, and multiple hybrid conformations, illustrated in Fig. 1), depending on the type of loops and guanine glycosidic bond orientation. Parallel G4s are characterized by propeller-type loops, connecting the upper and lower G-tetrads. Among the anti-parallel G4s, those adopting a chair conformation contain only lateral loops, while those with both lateral and diagonal loops adopt a basket conformation. Conversely, hybrid or mixed topologies can contain all three types of loops. In case of anti-parallel and hybrid G4s, both anti- and syn-oriented guanines can be found, while parallel ones exclusively contain anti-guanines [2]. Bioinformatic analysis and next-generation sequencing data demonstrated the wide distribution of G4s throughout the human genome, with the highest prevalence at telomeric ends and in promoter regions of proto-oncogenes, suggesting their pivotal role in gene regulation and telomere maintenance [3–6]. G4s are found in RNA sequences as well, where they might play important roles in RNA maturation and translation regulation [7]. Due to their specific occurrence in these strategic areas, and supported by the recent discovery of G4-formation in living cells using G4-specific antibodies (BG4 and SG4) as well as by sequencing-based evidence [8–10], G4s have gained increased interest as promising therapeutic targets in cancer research.

**Figure 2.**
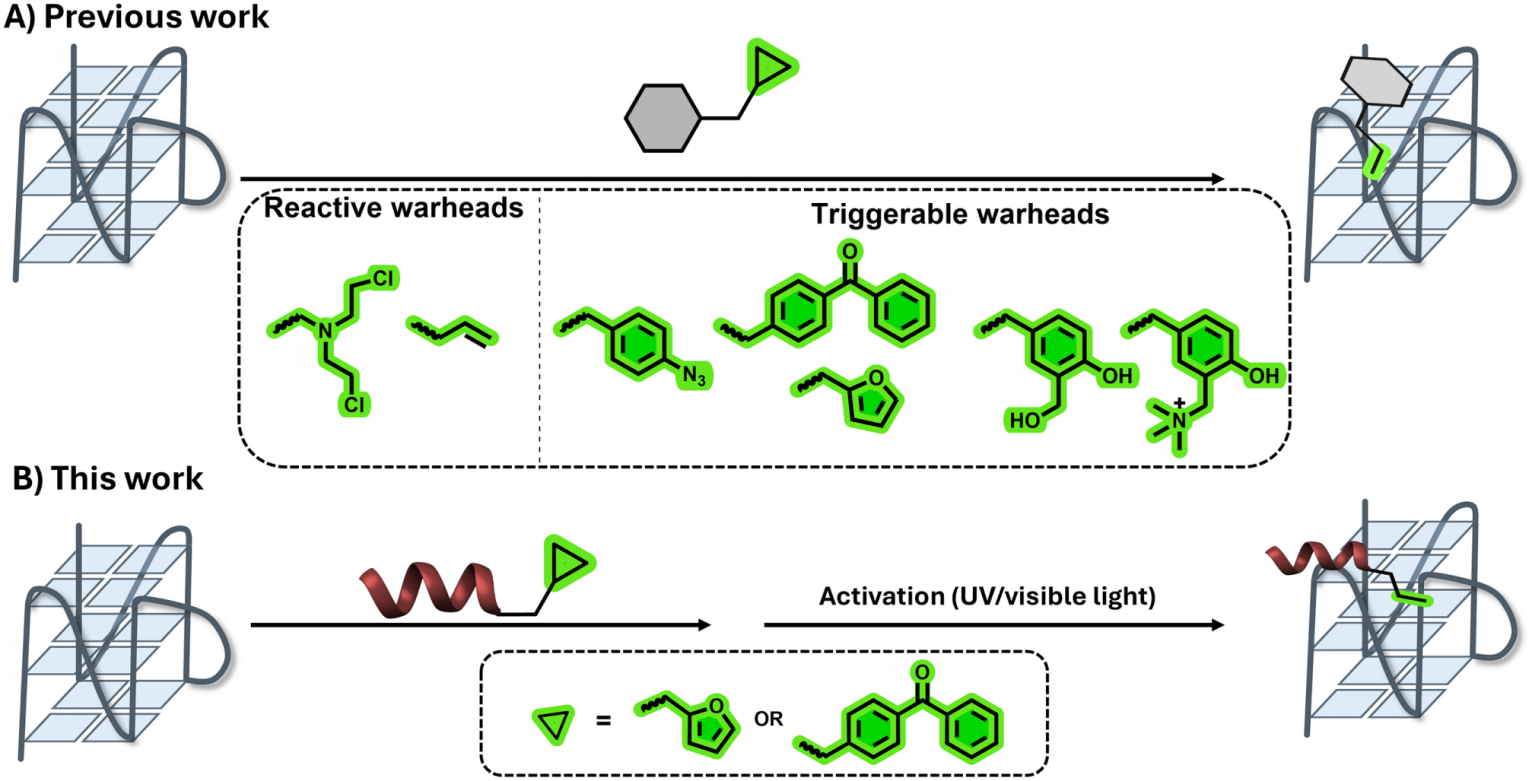
(**A**) Overview of the reported (non)-triggered alkylation strategies. (**B**) Proposed alkylation methodology in this work.

A variety of small molecules have been designed and investigated for their G4-binding capacity. In general, G4 ligands share common structural features, such as a planar aromatic core (allowing π–π-stacking with the G-tetrads) and positively charged groups (engaging in electrostatic interactions with the nucleic acid backbone). The vast majority of ligands interact with G4s via stacking on the outer G-tetrads; however, intercalating and groove/loop binding modes have also been reported [11]. Some ligands are even able to induce a topological shift upon binding [12–15]. In addition to non-covalent stabilizing approaches, various covalent strategies have been explored for G4-targeting, mainly relying on the decoration of well-established G4-binders with reactive moieties, such as nitrogen mustards [16] or vinyl derivatives [17] (Fig. 2A). A more sophisticated approach consists in the use of a system that can be activated upon demand for covalent bond formation, allowing for spatiotemporal control over the reaction. Among the potential triggers, light represents a highly controllable and easily accessible source of activation that has been applied in combination with several reactive groups (e.g. phenols [18], quinone methides [19], arylazides, benzophenones [20], and furan derivatives [21, 22]) to achieve G4-alkylation.

Peptides, such as those derived from proteins able to bind G4s, represent a promising alternative to small molecule ligands [23]. Given their high G4-specificity, inherently derived from mimicking the natural protein-G4 interactions, ease of synthesis, modular structure, and straightforward introduction of modifications, peptides offer several advantages compared to small molecules [24, 25]. In this work, we exploited the benefits of peptide-based ligands to develop a covalent G4-targeting methodology (Fig. 2B) using a peptide derived from the N-terminal domain of the RHAU protein. Inspired by the large thermal G4 stabilization that one of the modified alkylating peptides was able to induce even before activation, we further evaluated various N-terminal RHAU modifications, ultimately providing a chemical toolbox of new, biocompatible ligands with very high affinity for biologically relevant G4-DNA.

## Materials and methods

### Peptide synthesis

All peptides were synthesized according to standard Fmoc/t-Bu solid-phase peptide synthesis. Peptide elongation was performed on Fmoc Rink amide AM resin (0.64 mmol/g) as solid support using the automated MultiPep Rsi (Intavis). First, the resin was swollen for 30 min in DCM, followed by Fmoc-deprotection using 40% piperidine in DMF. Subsequently, to 5 eq. of amino acid and 5 eq. of HBTU in DMF, 10 eq. of DIPEA were added for each coupling step. The reactors were shaken for 30 min at room temperature, and the coupling procedure repeated twice. After the coupling, a capping step was performed using a 5/6/89 acetic anhydride/DIPEA/DMF solution (1 × 5 min). For modification of the lysine side chain (K6 + FurA and K6 + BPA), Fmoc-Lys(Dde)-OH was used. The Dde-protecting group was removed manually by stirring the resin for 1 h in a solution of 250 mg hydroxylamine HCl and 184 mg imidazole in 1 ml dry DMF. The side chain was then modified by the coupling of the photo-crosslinking amino acid. Once completed, the peptides were cleaved from the solid support by treatment with an optimized cleavage cocktail, taking into account the acid-lability of the furan [26, 27] and the oxidation-prone methionine (81.5% w/w trifluoroacetic acid, 5% w/w meta-cresol, 5% w/w triisopropylsilane, 5% w/w water, 2% w/w dimethylsulfide, and 1.5% w/w ammonium iodide) at room temperature for 1.5 h stirring. The peptides were, prior to purification, supplemented with a spoon tip (5 mg circa) of ammonium iodide and left for 1h to react. Then, they were purified by RP-HPLC (HPLC-2 conditions) and characterized by MALDI-TOF and LC-MS (Supplementary Table S1 and Supplementary Figs. SA1–SA16 in the Appendix).

### CD analysis

For analysing the peptide signature, 250 µl of 11 µM peptide in 20 mM Tris HCl, pH 7.4 buffer containing 10 mM KCl was prepared. A CD scan over a wavelength range of 190 to 320 nm was performed both in or without the presence of 5 µM G4-DNA (using T95-2T as reference), with a continuous scanning speed of 50 nm min^−1^, a response time of 1 s, 10 nm bandwidth, and 1 nm data pitch. For analysing the DNA signature, a 150 µl of 5 µM DNA in 20 mM Tris HCl, pH 7.4 buffer was prepared. 25 mM of KCl was added to obtain the hybrid fold of h-TELO, while 25 mM of NaCl was added to obtain its anti-parallel fold. 2.2 eq. of peptide were added, and the sample was left to equilibrate for a specific time. Each sample was measured in triplicate. All CD spectra were analysed and smoothed using GraphPad Prism 10.1.2 software. Additionally, CD spectra of RHAU18 (**1**), Nt + FurA (**6**), and Nt + His (**12**) were recorded in 5 mM KH_2_PO_4_/K_2_HPO_4_ buffer (pH 7.0) containing 20 mM KCl, both in the absence and presence of the c-Myc G4 at 25°C over a wavelength range of 200–320 nm. These spectra were acquired using a scan rate of 100 nm/min, with a 2 s response time and 2 nm bandwidth, and represent the average of three scans with the buffer baseline subtracted. The CD spectrum of the c-Myc G4 alone was also acquired using the same conditions and then subtracted from the corresponding G4/peptide mixtures to isolate the spectral contribution of the peptides in the presence of G4. The percentage of secondary structure content adopted by each peptide in the absence and presence of G4 was estimated using the BeStSel software [28].

### Alkylation experiments

Five micromolar DNA solutions (Supplementary Table S2) were freshly prepared in a 20 mM phosphate (K_2_HPO_4_) pH 7 buffer. To these working solutions, 2.5 eq. (up to 12.5 µM) of the allocated peptide was added and left equilibrating for 1 h. For the furan-containing peptides, 20 µM of PS was added, and the samples were irradiated with light (500 nm) for 1.5 h. For the benzophenone-containing peptides, the samples were exposed for 10 min to UV-A light (360 nm). Before light irradiation, each sample was aliquoted in a blank (no light exposure) and tested sample (light exposure). All DNA solutions were pre-annealed by heating for 5 min at 95°C and cooled down to ambient temperature over a period of 4 h. Finally, the samples were shielded from light and analysed by RP-HPLC-UV (HPLC-4; see general information in SI). Alkylation efficiencies were determined by the %DNA consumption, calculated via integration of the DNA peak. The formed reaction products were isolated, freeze-dried, and analysed by MALDI-TOF to characterize the formed alkylation product. HPLC alkylation experiments were performed in duplicate.

### UV melting experiments

One hundred fifty microliters of 5 µM DNA in 20 mM Tris HCl, pH 7.4, buffer was prepared. For c-Myc and T95-2T, up to 25 mM NaCl was added, while for BCL-2, c-Kit2, VEGF, and h-TELO up to 10 mM KCl was added. Each experiment was performed without or in presence of 2.2 equivalents of peptide. The annealing was performed inside the instrument by heating up to 95°C in 10 min, followed by a slow cooling ramp of 1 h to 15°C. The absorbance at 295 nm (for G4-DNAs) or 260 nm [for double-stranded DNA (dsDNA)] was followed during both melting (15–95°C) and annealing (95–15°C) ramps, at a scanning speed of 1°C/min. The *T*_m_ values were calculated based on the melting ramps using Boltzmann sigmoidal function in the GraphPad Prism 10.1.2 software. Each *T*_m_ represents the mean value of triplicate measurements.

### Isothermal titration calorimetry experiments

Isothermal titration calorimetry (ITC) experiments were carried out at 25°C using a nano-ITC Low Volume calorimeter (TA Instruments, Lindon, UT, USA). To ensure consistency in buffer composition and pH, G4 and peptide solutions were prepared from the same buffer batch (5 mM KH_2_PO_4_/K_2_HPO_4_ buffer, pH 7.0, containing 20 mM KCl). The c-Myc solution (23–25 μM) was titrated with 25 injections of peptide solution (2 µl, 313–340 μM) into the calorimetric cell (190 μl), with a 300 s interval between injections to allow the system to reach equilibrium. All ITC measurements were performed using non-activated peptides, and the thermodynamic parameters reported correspond to the non-covalent binding of the peptides to the G4 structure. A blank titration was performed by injecting the peptide solution into the buffer alone to account for dilution effects. The corrected heat values were plotted against the molar ratio to obtain the corresponding binding isotherms. The experimental binding isotherms were analysed using the NanoAnalyze software (TA Instruments), fitting them to a theoretical model to determine binding enthalpy (Δ*H*°), equilibrium binding constant (*K*_a_), and interaction stoichiometry (*n*). The Gibbs free energy (Δ*G*°) and entropy (*T*Δ*S*°) changes were calculated using the following equations: Δ*G*° = −*RT* ln *K*_a_ (*R* = 8.314 J mol^−1^ K^−1^, *T* = 298.15 K) and *T*Δ*S*° = Δ*H*° − Δ*G*°. The results reported represent the average of at least two independent experiments.

### NMR analysis

NMR experiments were performed at 25°C on a Bruker Advance NEO NMR spectrometer (Bruker BioSpin, Rheinstetten, Germany) operating at 600 MHz (1H Larmor frequency), equipped with a 5-mm QCI cryo-probe set and a cooled SampleJet autosampler. DNA samples were prepared at 0.05 mM strand concentration in 0.6 ml of buffer solution (5 mM KH_2_PO_4_/K_2_HPO_4_ buffer, pH 7.0, containing 20 mM KCl), supplemented with 10% D_2_O. Aliquots of peptide stock solution (5 mM in water) were added to the DNA solution, followed by an equilibration of 5 min before acquiring the spectra. Peptide spectra were recorded at the same concentrations used for titrations. One-dimensional proton spectra were recorded using excitation sculpting with gradients for water suppression [29], and 512 scans for spectrum with a recovery delay of 1.5 s. The Fourier transformed spectra were phase adjusted, baseline corrected and calibrated with respect to the 4,4-dimethyl-4-silapentane-1-sulfonic acid, DSS, used as a reference to calibrate the chemical shifts, with the DSS methyl groups set at 0.0 ppm. NMR spectra were processed and analysed using TopSpin 4.3.0 (Bruker), and MestReNova software.

## Results and discussion

### Design and characterization of G4-alkylating peptides

**Figure 3.**
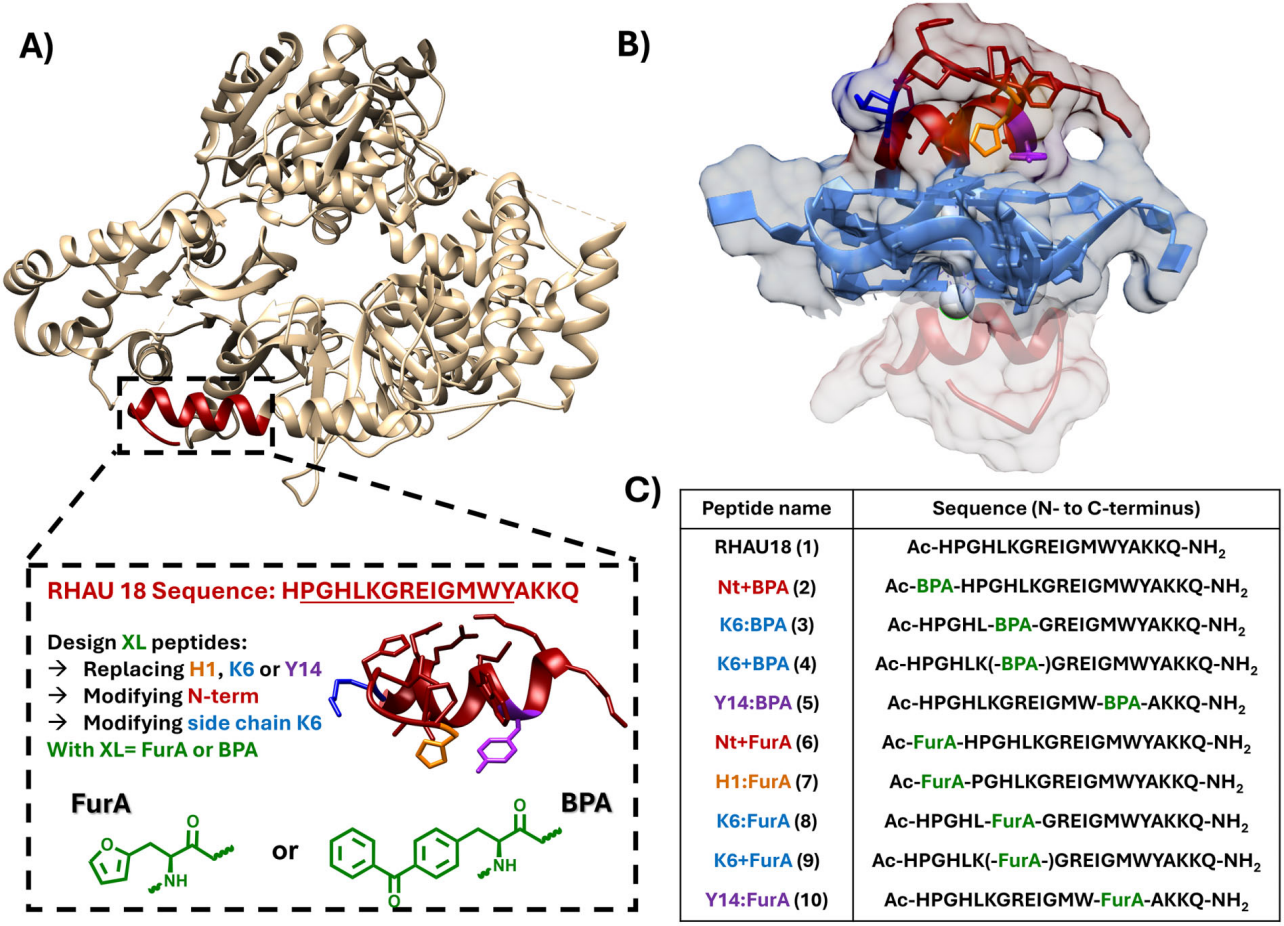
(**A**) Design of the crosslinking peptides derived from the N-terminal domain of the RHAU helicase. The coloured residues were either replaced (:) or elongated (+) with a crosslinking amino acid (XL), either L-furyl-alanine (FurA) or L-BPA. (**B**) Illustration of the sandwich binding mode of two RHAU18 peptides in complex with T95-2T G4. PBD 5N9A obtained from [35] and PDB 2N21 adapted from [31]. (**C**) Overview of all synthesized peptides, containing either no XL (RHAU18), BPA or FurA as XL. The numbering of the peptides (in brackets) refers to the nomenclature used throughout the text.

In this study, the RHAU18 sequence was utilized as a reference to design multiple photo-crosslinking peptides. RHAU (or DHX36) is a DEAH (aspartic acid–glutamic acid–alanine–histidine)-box helicase, able to unwind DNA- and RNA-G4s. It features a well-documented N-terminal domain, comprising the 13 amino acid-long RHAU specific motif (RSM), that is responsible for G4-recognition (Fig. 3A) [30]. Previously, the Phan group investigated the interaction of RHAU peptides of varying lengths with both parallel and non-parallel G4s. These peptides have been shown to bind through stacking interactions at the 5′-G-tetrad (Fig. 3B) and consequently bind preferentially to parallel G4s, where the outer G-tetrads are more accessible compared to non-parallel G4 topologies. Moreover, they reported the NMR solution structure of RHAU18 in complex with the parallel T95-2T G4 [31]. This fragment of the full N-terminal domain of RHAU represents the best compromise in terms of length and target binding affinity, thus serving as a good model for our covalent G4-targeting strategy, in which two commercially available crosslinker-bearing amino acids, L-furyl-alanine (FurA) and L-benzoyl-phenylalanine (BPA), can be introduced at specific positions within the peptide sequence.

The peptide adopts a L-shaped α-helical structure spanning from Gly3 to Ala15, with a sharp kink before residue Gly7, which is crucial for G4-recognition [31]. More specifically, Pro2, Leu5, and Trp13 are essential to maintain the conformation of the peptide, while Gly7, Ile10, Gly11, and Ala15 are involved in stacking interactions with the top G-tetrad [31, 32]. Based on these structural insights, rationalized substitutions and modifications were introduced to preserve the binding affinity and conformation of the peptide (see Fig. 3C for the design and nomenclature used). FurA and BPA were selected among the available crosslinkers for their effectiveness in earlier peptide–peptide [33, 34] and ligand-G4 [20, 21] photo-labelling studies. As Lys6 does not appear to be crucial for binding to the target, we reasoned it could be substituted by either of the crosslinking amino acids. Besides completely replacing Lys6, alternatively, the Lys6 side chain was also extended by adding a photo-crosslinking moiety. While replacement of Tyr14 by non-aromatic amino acids appeared to be detrimental for target binding [31], replacement with the aromatic residues FurA and BPA was considered sensible. Finally, His1, which is neither part of the α-helix nor the RSM, was substituted with the isosteric FurA.

**Figure 4.**
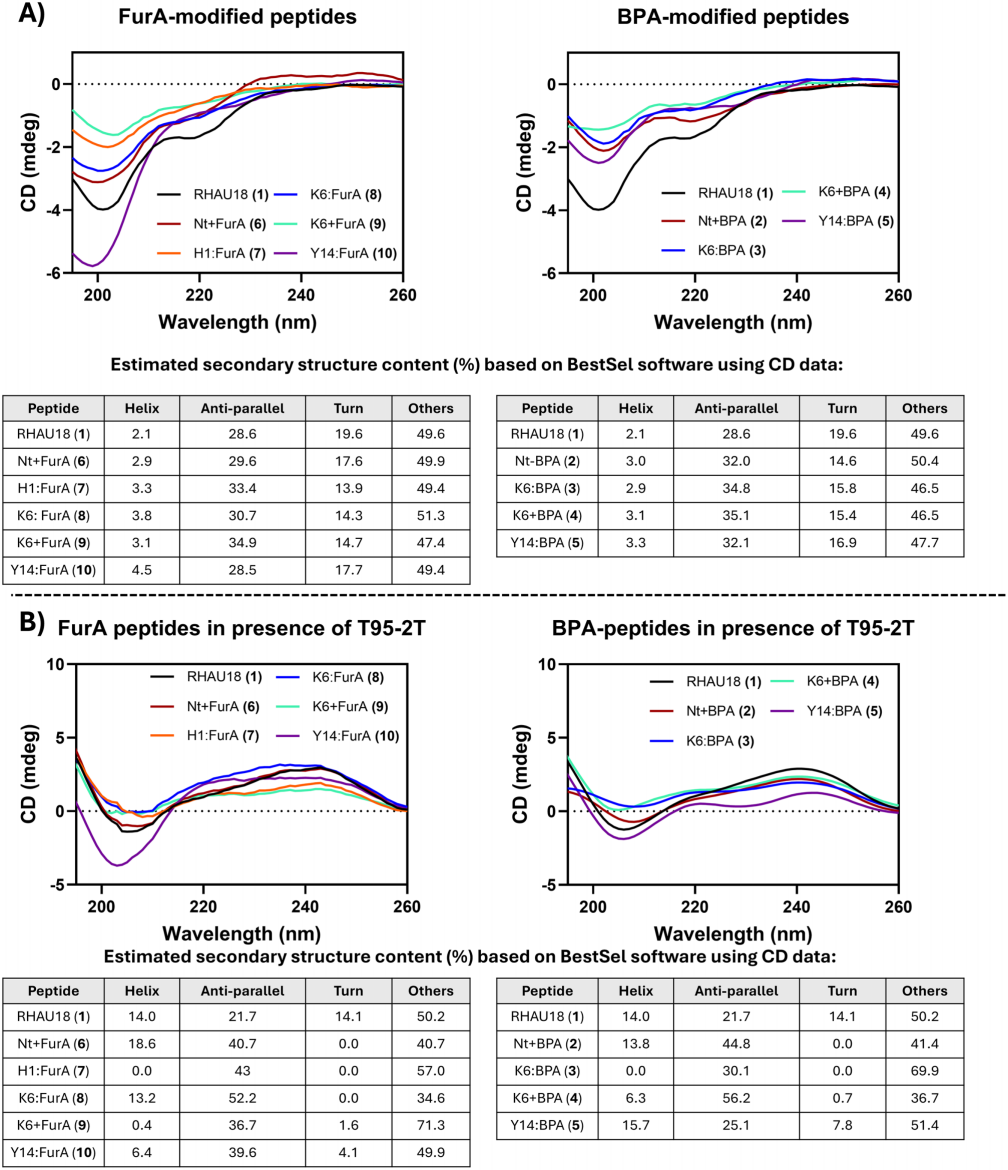
(**A**) CD spectra and estimated secondary structure content (including α-helix, antiparallel β-sheets, turns, and other conformations) of the FurA and BPA peptides. (**B**) Subtracted CD spectra and estimated secondary structure content (including α-helix, antiparallel β-sheets, turns, and other conformations) of the FurA and BPA peptides in presence of parallel G4-sequence T95-2T. All CD experiments were performed in buffered solution (20 mM Tris HCl, pH 7.4, supplemented with 10 mM KCl) at 11 µM peptide concentration (with or without 5 µM DNA).

To evaluate the influence of each modification on the peptide folding, the secondary structure of each peptide was analysed by circular dichroism (CD) spectroscopy. Typically, α-helical peptides display two negative bands at around 205 and 220 nm [36], whereas peptides adopting a random conformation exhibit very low ellipticity above 210 nm and a negative band near 200 nm. The acetylated wild-type RHAU18 (peptide **1**) showed a spectrum consistent with a predominantly disordered structure, with only a minor α-helical contribution (2.1%), as summarized in the table in Fig. 4A. A similar behaviour was observed for the modified analogues of peptide **1** (peptides **2–10**), which also displayed spectra characteristic of largely unstructured conformations in solution. Additional CD experiments were conducted to investigate potential conformational changes in the peptides upon binding to a G4 (T95-2T). By subtracting the CD signal of the G4 from that of the corresponding G4/peptide mixture [23, 36], an increase in the estimated α-helical content was observed forpeptide **1** and for most FurA- and BPA-containing analogues, consistent with observations reported for other G4-binding peptides [23, 36–38]. Exceptions were peptides **3, 7**, and **9**, whose helical content did not significantly increase, or even decreased, upon G4 binding (Fig. 4B).

### Optimization and evaluation of peptide-G4-alkylation reaction

In total, nine modified peptides were synthesized by incorporating one of the selected photo-crosslinkers (FurA or BPA) into the peptide sequence and evaluated for their alkylation potential. The pro-reactive furan moiety, upon oxidation by singlet oxygen (^1^O_2_) generated by visible light irradiation of a photosensitizer (PS), forms an electrophilic keto-enal via a Diels-Alder reaction. This electrophile can subsequently react with proximate nucleophilic amines of the DNA or RNA nucleobases [27]. Benzophenone, on the other hand, requires UV-A irradiation to convert into a reactive species that can, in principle, undergo multiple reactions with DNA: (i) a [2 + 2] cycloaddition via a Paterno-Buchi reaction with pyrimidine bases, (ii) benzophenone-mediated photosensitized reaction of thymines resulting in the formation of cyclobutene thymine dimers (CTDs) via a triplet-triplet energy transfer mechanism, (iii) radical-mediated covalent bond formation upon hydrogen abstraction, or (iv) guanine oxidation through the formation of ^1^O_2_ [39].

In an initial set-up, the activation conditions were optimized for each photo-crosslinker, using either peptide **3** or **8** containing BPA or FurA instead of Lys6, respectively, to reach complete activation and at the same time avoid excessive (UV-A) light exposure, which might lead to oxidative damage or CTDs. For peptide **3**, complete conversion was observed after 10 min of UV-A irradiation (λ = 360 nm) (Supplementary Figs. S1 and S2B), whereas peptide **8** required either green light irradiation (λ = 500 nm) for 90 min in the presence of Rhodamine B (RhoB) or 15 min for Rose Bengal (RB) as PSs, or red light irradiation (λ = 650 nm) for 15 min in presence of Methylene Blue (MB) as PS (Supplementary Figs. S2A and S3). There it can also be seen that, rather than a single, well-defined activation product, a broad signal at earlier retention times was observed suggesting the formation of a mixture of products. For example, in the absence of a G4-target, the activated peptides may react via intra- or intermolecular reactions due to the presence of nucleophilic or other reactive side chains. Additionally, for the FurA series, the generation of ^1^O_2_ can lead to oxidative alterations of the peptide, as it contains multiple oxidation-sensitive amino acids, such as Met, Trp, and Tyr [40].

In a preliminary alkylation experiment, using c-Kit2 as the G4-target, three PSs were tested in combination with peptide **6** to assess their G4-alkylation capacity. A 1:2.5 molar ratio between G4 and peptide was employed, since RHAU18 appeared to have an optimal binding at a 1:2 G4:peptide stoichiometry [31]. Despite being the least effective PS, RhoB (^1^O_2_-QY < 0.1) yielded the highest alkylation efficiency (30%) compared to MB (20%) and RB (12%) (Supplementary Fig. S4). A possible explanation for the lower efficacy of those stronger PSs (^1^O_2_-QY of 0.52 for MB; ^1^O_2_-QY of 0.75 for RB [41]) is that they may over-oxidize the peptide, potentially altering its structure and reducing binding to the G4 target. Alternatively, MB, known to be a G4 binder [42], may compete with the peptide for the same G4-binding site, thereby disturbing the correct positioning of the keto-enal moiety. Considering these observations, RhoB was selected as the preferred PS for oxidation of the furan-containing peptides. Moreover, the use of a milder PS, such as RhoB, also minimizes the risk of guanine oxidation (Supplementary Fig. S5) [41].

**Figure 5.**
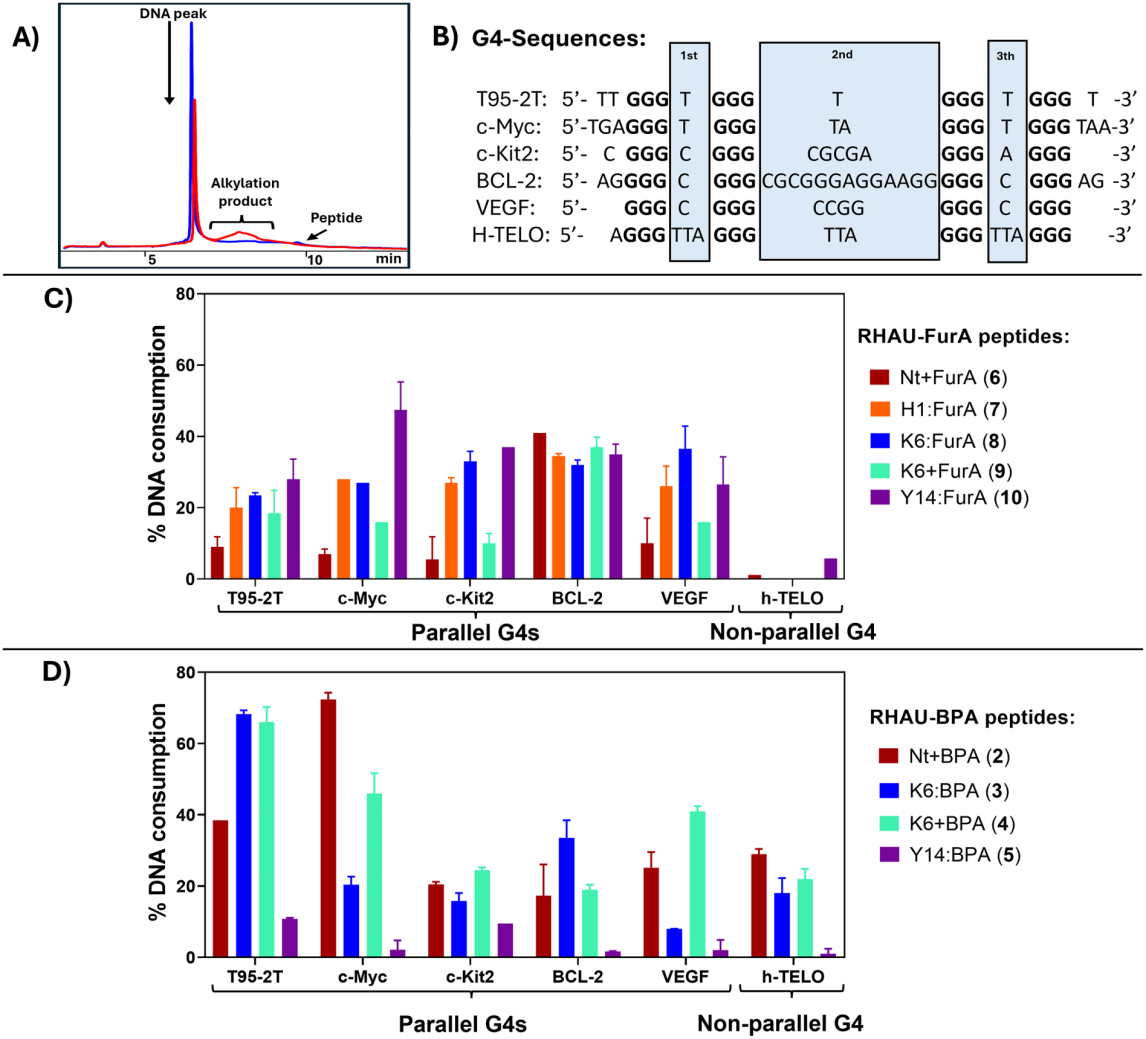
Overview of the alkylation experiments: (**A**) Example of a typical HPLC chromatogram before (blue) and after (red) irradiation. (**B**) Selected G4-DNA sequences in this study, with their loops highlighted in the blue box. (**C**) Alkylation efficiency of each FurA-containing peptide for all tested G4 sequences. (**D**) Alkylation efficiency of each BPA-containing peptide for all tested G4 sequences. Experiments were performed in a buffered solution (20 mM phosphate, K_2_HPO_4_, pH 7 buffer) at 5 µM strand concentration in a 1:2.5 DNA:peptide ratio.

The alkylation potential of the alkylating peptide library was then evaluated on both parallel (T95-2T, c-Myc, c-Kit2, BCL-2, and VEGF) and non-parallel (h-TELO) G4s by RP-HPLC. We have previously established an HPLC-based quantification method for G4-DNA alkylation by following the consumption of the DNA starting material (via integration of the DNA peak before and after reaction) [21]. This provides a straightforward means of quantifying alkylation efficiency, as the direct quantification of the resulting alkylation products is often challenging. Indeed, these products typically consist of a mixture of products that yield broad peaks eluting at higher retention times than the DNA (Fig. 5A). In some cases, the unreacted, but activated, probes may co-elute at similar retention times, making quantification of the product formation equivocal.

In general, for T95-2T, a three-layered G4 connected by single-thymine (T) loops (Fig. 5B), the lowest alkylation yields were obtained with the FurA series (Supplementary Figs S6–S10). This can be attributed to the limited number of potential reaction partners, as the activated keto-enal, resulting from furan-oxidation, can only react with exocyclic amines of guanine (G), cytosine (C), and adenine (A) [21], implying that, in this specific case, the furan can only target the guanines constituting the G-tetrads. In a stable G4, the bases in the G-tetrad are tightly bound in a Hoogsteen hydrogen-bonding network, limiting the availability of the exocyclic G amine for reaction with the alkylating moiety. Peptide **6** exhibited low alkylation efficiency for most of the parallel G4 targets, except for BCL-2, for which a similar alkylation yield of around 40% was found for all FurA-modified peptides (Fig. 5C). The BCL-2 G4 has a distinctive structure, since it contains a remarkably long middle loop (13 nt) compared to the other parallel G4s (Fig. 5B) [43]. This long loop, exclusively consisting of residues equipped with exocyclic amines, might be in close vicinity to the furan, increasing the chance of reaction with** 6**. Furan modifications at Lys6 (**8**) and (**9**)result in a distinct pattern of DNA consumptions, with higher yields obtained for peptide **8** in combination with the shorter middle-loop G4s (T95-2T, c-Myc, c-Kit2, and VEGF). Finally,peptides **7** and **10** showed low to moderate (15%–30%) and moderate to high (30%–50%) yields, respectively.

Alternatively, as mentioned above, benzophenone can react with DNA through multiple mechanisms [39], potentially enabling higher alkylation efficiency. Indeed, very high alkylation yields were obtained for T95-2T (65% with** 3 ** and 62% with **4**) and c-Myc (72% with **2 ** and 50% with **4**) (Fig. 5D and Supplementary Figs. S11–S14). In contrast, low DNA consumptions were observed for peptide **5** for all tested sequences. A possible explanation might be that, due to the substitution of the Trp residue with BPA, the peptide binds in another orientation with the G4 targets, wherein the benzophenone can be positioned away from suitable reaction partners. The formation of covalent peptide-G4 adducts was confirmed by MALDI-TOF and MS analysis (Supplementary Figs S15–S17).

Similar observations could be made when analysing the alkylation reaction of T95-2T with the BPA series by denaturing gel analysis. Two conditions were tested, each optimized for the migration of either the DNA or peptide components (denaturing polyacrylamide gel electrophoresis (PAGE) with SYBR gold staining, and USDS–PAGE with silver staining, respectively). USDS–PAGE represents the first instance in which both peptide and DNA could be visualized using a single staining method. Under these conditions, clear alkylation products were observed for the BPA series (Supplementary Fig. S18, panel B), appearing as bands between the peptide and DNA signals. In contrast, for the FurA-peptides, the band corresponding to the alkylation products likely falls below the detection limit of the silver staining method (Supplementary Fig. S18, panel A). After light-triggered activation of the furan, the peptide seems to be fully consumed. This can be explained by its oxidation sensitivity. Indeed, in addition to the furan, the RHAU peptide contains multiple amino acids that may oxidize during the photoactivation step, resulting in disappearance of the peptide band. In the case of the BPA-peptides, the disappearance of the peptide band is correlated with the formation of new product bands.

In the SYBR-stained gel, which offers a greater sensitivity for DNA detection, a faint, upper band was detected (Supplementary Fig. S19, panel A). Densitometric analysis of the bands corresponding to the DNA starting material in USDS–PAGE (for T95-2T, panel B in Supplementary Fig. S19) was performed and demonstrated a trend consistent with the data obtained using the HPLC analysis (Supplementary Table S3).

The alkylation yield of both the FurA and BPA series was also determined for non-parallel (h-Telo) G4-DNA, dsDNA, and single-stranded DNA. The FurA peptides showed minor alkylation of the hybrid h-TELO [44] (Supplementary Fig. S20) and no consumption of non-G4-DNA (Supplementary Fig. S21). This lack of reactivity observed for the telomeric G4 can be explained by the unavailability of a suitable reaction partner. Indeed, furan-based alkylation reactions are highly dependent on the close proximity of an available reaction partner. Since furan is known to react with the exocyclic amines of adenine, cytosine, and guanine [21, 22], the potential reaction partners in the h-TELO sequence are limited to the adenines present in the loops and the guanines involved in G-tetrad formation, though they are more shielded by the Hoogsteen hydrogen-bond network.

The BPA peptides (except** 5**) were able to covalently target non-parallel G4s, with moderate alkylation yields forpeptides **2** (27%), 4 (22%), and **3** (16%) (Supplementary Fig. S22), while clearly preferring G4 over dsDNA (Supplementary Fig. S23). In a control experiment, we exposed RHAU18 (**1**), which is not functionalized with a photo-crosslinker, to the FurA and BPA activation conditions in presence of a G4-target (c-Kit2). As expected, no DNA consumption or alkylation products were observed (Supplementary Fig. S24).

Similar conclusions were drawn when performing the alkylation reaction of a parallel G4 (c-Kit2 was used for the FurA-containing peptides and T95-2T for the BPA-containing peptides) in the presence of either a non-G4 sequence (dsLAC) or the hybrid h-TELO G4 sequence. All peptides retained their high G4-selectivity, as evidenced by the neat decrease in the G4-DNA peak, without affecting the non-G4 sequence (Supplementary Fig. S25). Notable differences were observed in the alkylation efficiency of c-Kit2 and T95-2T with their respective peptides when comparing single target (Fig. 5) with competition experiments (Supplementary Fig. S25). We attribute this to the possible differences in the orientation of the photo-activated warheads towards the G4 structure, likely influenced by the presence of the bulky fluorophore label. In the competition experiments involving two G4 sequences, one hybrid (h-TELO) and one parallel (T95-2T or c-Kit2), the outcomes appeared to be crosslinker-dependent (Supplementary Fig. S26). Peptides **9** and **10** did not react with the hybrid h-TELO, whereas peptides **3** and **4** showed low to moderate levels of alkylation. Since the obtained yields were only marginally affected when replacing h-TELO with dsLAC in the competition experiment, we assume that the peptides preferentially bind to parallel G4 sequences.

### Thermal stabilization of G4s induced by crosslinker-modified peptides

**Figure 6.**
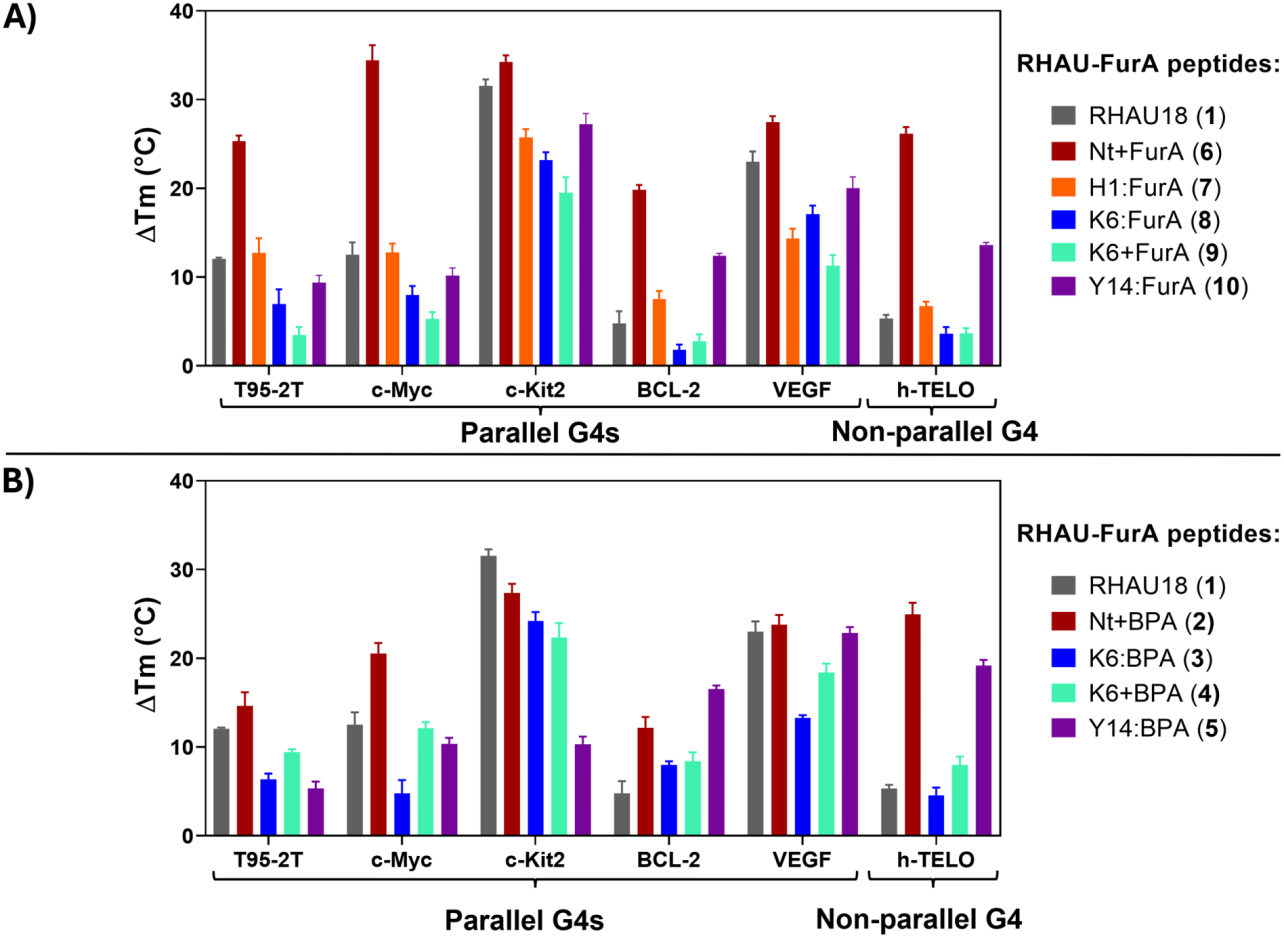
Induced thermal stabilization of each FurA (**A**) and BPA (**B**) peptide on both parallel (T95-2T, c-Myc, c-Kit2, BCL-2, and VEGF) and non-parallel (h-TELO) sequences, derived from UV melting experiments. Melting experiments were performed in a buffered solution (20 mM Tris HCl, pH 7.4 buffer, supplemented with 10 mM KCl for BCL-2 and h-TELO or 25 mM NaCl for T95-2T and c-Myc) at 5 µM strand concentration in a 1:2.2 DNA : peptide ratio.

Thermal denaturation experiments were carried out to assess the non-covalent stabilizing effect of each peptide on the different G4s, using a 1:2.2 molar ratio between DNA and peptide (Supplementary Figs S27–S29 and Supplementary Table S4), assuming that one peptide binds to the 5′-G-tetrad and the second peptide covers the 3′-G-tetrad, forming a ‘sandwich-like’ complex with the G4 [31]. In general, RHAU derivatives are reported to be good stabilizers of several parallel G4s [38]. UV-melting analysis of the G4s in presence of RHAU18 (**1**) confirmed this (Fig. 6), showing Δ*T*_m_ (variation of melting temperature) values above 10°C for T95-2T, c-Myc, c-Kit2, and VEGF G4s. Upon modification, the peptides retained the ability to stabilize G4s, with the degree of stabilization depending on the specific G4, and exhibited high selectivity for G4 over dsDNA (Supplementary Fig. S29). Remarkably, all peptides (except peptide **5**) strongly stabilized c-Kit2 with Δ*T_m_* values ≥20°C. Literature reports also support the introduction of furan residues into G4 ligands to tweak the binding affinity and stabilization potential [45, 46]. Peptide **7** showed Δ*T*_m_ values similar to those of RHAU18 (**1**) for most G4s. Modifications involving replacement (peptides **3** or **8**) or capping (peptides **4** or **9**) of the positively charged Lys6 side chain, which mediates electrostatic interactions with the G4-DNA backbone, slightly decreased the stabilization for most of the G4s. Substitution of Tyr14 with FurA (peptide **10**) or BPA (peptide **5**) had high G4-specific effects, resulting either in an enhancement (for BCL-2 and h-TELO), reduction (for T95-2T and c-Kit2), or negligible change (for c-Myc) in stabilization. Most notably, modification of the N-terminus seemed to have a pronounced positive effect on the stabilization of parallel G4 structures, particularly for T95-2T and c-Myc (both in Na^+^- and K^+^-containing solutions, Fig. 6 and Supplementary Figs S30 and  S31). The aromatic groups, BPA (peptide **2**) and FurA (peptide **6**), at the N-terminus can introduce additional stacking interactions with the G-tetrads and thereby enhance target stabilization. This may explain the explicit increase in Δ*T*_m_ values, which occurs without formation of any covalent linkage, as verified by HPLC analysis (see Supplementary Fig. S32). While RHAU peptides are known to preferentially bind parallel G4s, significant thermal stabilization of the h-TELO hybrid G4 was also observed, especially by the N-terminally modified peptides **2** and **6**, but also by Tyr14-modified peptides **5** and **10**, suggesting that a G4 conformational shift event may take place.

### Shape-shifting effect of G4-alkylating peptides

**Figure 7.**
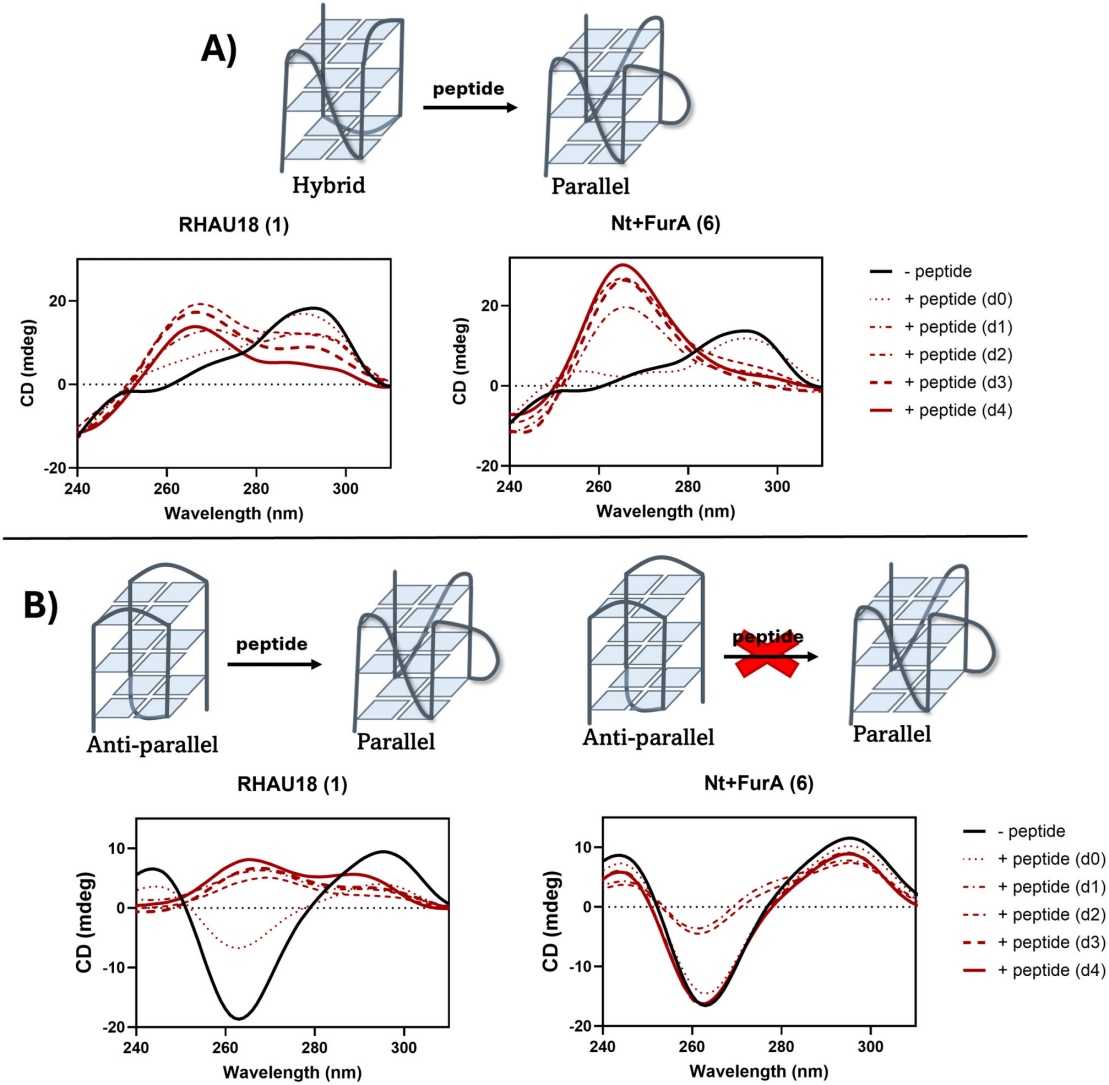
Overview of CD kinetic shift experiments: (**A**) Indication of the conformational transitioning from h-TELO hybrid to h-TELO parallel for RHAU18 (**1**) and Nt + FurA (**6**). (**B**) Indication of the conformational transitioning from h-TELO anti-parallel to h-TELO parallel for RHAU18 (**1**) while Nt + FurA (**6**) did not induce the conformational transitioning. CD experiments were performed in a buffered solution (20 mM Tris HCl, pH 7.4 buffer) at 5 µM strand concentration in a 1:2.2 DNA:peptide ratio (d0 refers to the direct measurement after addition of the peptide, d1–4 = measurement 1–4 days after addition of the peptide). For the hybrid h-TELO, 25 mM KCl was added to the solution; for the antiparallel h-TELO, 25 mM NaCl was added to the solution.

According to literature reports, the interaction between a RHAU-derived peptide, RHAU25, and the telomeric G4 sequence TA(GGGTTA)_3_ induces a conformational transition from anti-parallel or hybrid to parallel topology [38]. For this hybrid G4, importantly, the phenomenon was only observed after annealing the G4 in the presence of the peptide. To verify whether our modified peptides could also induce this conformational conversion, CD experiments were performed. Hybrid G4s are characterized by a major positive band at 290 nm, a shoulder at 260 nm, and a negative band at 240 nm [47]. Similar to RHAU18 (**1**), peptides **2, 5, 6**, and **10** induced a conformational shift to a parallel fold, characterized by a major positive band at 260 nm and a negative band at 240 nm [47], when added during the annealing (Supplementary Fig. S33). We further questioned whether this conformational conversion could be a kinetically controlled process. Therefore, a kinetic experiment was performed by adding the peptide to the folded G4 and following the CD signature over a time span of 4 days (Fig. 7A). Interestingly, for peptide **6**, which resulted in the highest induced thermal stabilization, we observed the topological conversion to the parallel fold after 1 day, and the refolded G4 remained stable over the course of 4 days. The addition of RHAU18 (**1**), on the contrary, resulted in a mixed signature after 1 day. The increased intensity of the peak at 264 nm suggests partial presence of a parallel species, while the peak still present at 290 nm suggests the presence of antiparallel and/or hybrid species. During the next days, a progressive increase in the parallel fold was observed, though a fraction of non-parallel folds remained present even after 4 days. To quantify the relative populations of the different species, we performed singular value decomposition analysis of the CD spectra [48], which allowed accurate allocation of the spectral contributions and confirmed a progressive increase in the parallel topology over time (see Supplementary Fig. S34A for the estimated percentages of each topology). The same kinetic experiment was performed for the other FurA-modified peptides (**7-10**)(Supplementary Fig. S35), revealing a direct correlation between the kinetics of the G4 conformational shift and the stabilization potential of the peptides.

The synthesized peptides were also investigated for their ability to induce conformational conversion of h-TELO in sodium-containing buffer. Under these conditions, h-TELO adopts a basket-type anti-parallel G4 featuring two lateral and a diagonal loop [49]. Anti-parallel conformations show a CD signature with two positive bands around 295 and 240 nm and a negative one at around 260 nm [47]. Similarly to the hybrid h-TELO, two types of experiments were performed: (i) measurement of the CD signature of the G4 annealed in the presence of peptide and (ii) following the change of the h-TELO CD signature after addition of the peptide over a timespan. RHAU18 (**1**) was able to induce a conformational shift of h-TELO towards the parallel structure, whereas peptide **6** did not produce significant conformational changes (Supplementary Fig. S36). Comparable results were obtained in the kinetic experiments (Fig. 7B), where **6** first induced a conformational change towards a hybrid topology, with the hyperchromic effect at the minimum at 260 nm. However, this seemed to be a transient, unstable structure that rearranged within 4 days to the original anti-parallel conformation (Fig. 7B and Supplementary Fig. S34B). To ensure that this conformational change was not caused by degradation phenomena, HPLC analysis of the peptide was performed after the 4-day period, showing no signs of degradation, precipitation, or decomposition of the peptide (Supplementary Fig. S34).

**Figure 8.**
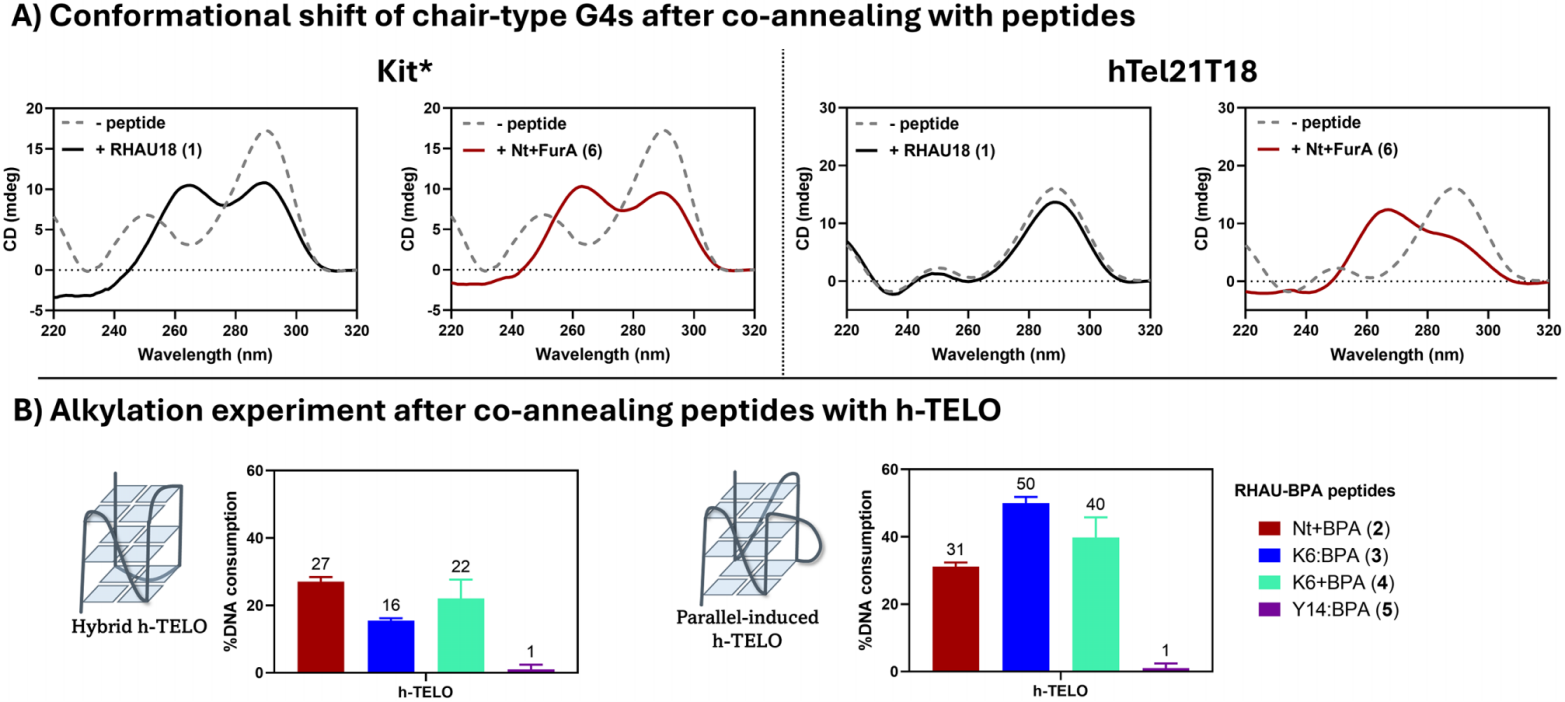
**A**) CD spectra of Kit* and hTel21T18 in 20 mM Tris HCl, pH 7.4 buffer (supplemented with 25 mM KCl), pre-annealed with RHAU18 (**1**) or Nt + FurA (**6**). The obtained spectra are compared with the signature of the two-quartet chair-type anti-parallel Kit* as such (grey dotted line, left) or the signature of the three-quartet chair-type anti-parallel hTel21T18 (grey dotted line, right). (**B**) Graph bars representing the alkylation experiment of h-TELO with Nt + BPA (**2**), K6:BPA (**3**), K6 + BPA (**4**), or Y14:BPA (**5**) either performed by adding the peptide to the annealed DNA and leaving the sample equilibrating for 1 h (left) or performed after preparing the G4-peptide mixture and annealing of this solution. Alkylation experiments were performed in a buffered solution (20 mM phosphate, K_2_HPO_4_, pH 7 buffer) at 5 µM strand concentration in a 1:2.5 DNA:peptide ratio.

A similar salt-dependent conformational shift of h-TELO observed for peptide **6** and other furan-derivatized RHAU peptides (**7-10**, Supplementary Fig. S38) was previously reported for small molecule N-methyl mesoporphyrin IX (NMM) [50]. Yatsunyk et al. ascribed this phenomenon to conformational selection, where NMM preferentially binds to the parallel fraction, influencing the structural equilibrium towards parallel topology [51]. Interestingly, the energy cost associated with the topological conversion from anti-parallel to parallel seems to be higher than that required for the hybrid-to-parallel shift [52]. Both RHAU18 (**1**) and peptide **6** were tested for inducing the conformational shift of two other anti-parallel G4s (kit* and hTel21T18T) to parallel structures (Fig. 8A). Interestingly, RHAU18 (**1**) only induced an increase in parallel content for kit*, whereas peptide **6** partially shifted the conformation of both kit* and hTel21T18T to predominantly parallel structures. This distinct behaviour (compared to the observations for the anti-parallel h-TELO) may be due to their chair-type G4 structures [53, 54], in which one G-tetrad is, in principle, more accessible for ligand binding compared to the basket-type h-TELO structure, which has edgewise-diagonal-edgewise loops covering the binding surface of the outer G-tetrads, hindering structural conversion.

In view of the shape-shifting behaviour of the modified RHAU peptides, the alkylation experiments with h-TELO were repeated by first co-annealing the peptide with the G4, thereby ensuring that the most stable, and thus presumably the parallel fold was adopted. For the RHAU-FurA peptides, no significant changes in alkylation yields were observed; these peptides remained unable to efficiently form covalent linkages with h-TELO, even when it rearranged in a parallel fold. In contrast, for the RHAU-BPA series, increased DNA consumption was observed for peptides **2** (27%–31%), **3** (16%–50%), and **4** (22%–40%) (Fig. 8B).

### Design and characterization of an extended set of N-terminal modified peptides

**Figure 9.**
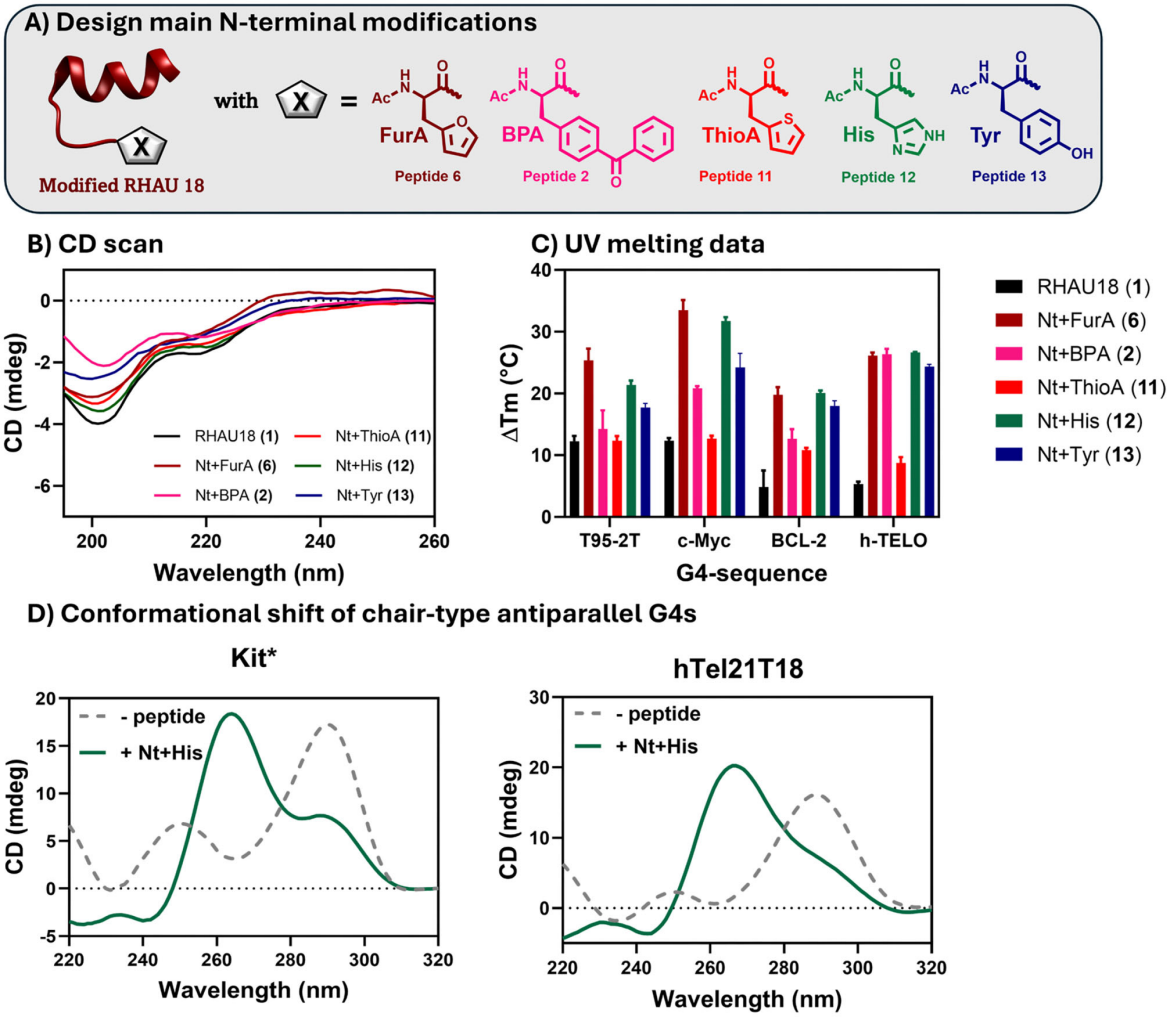
Analysis of N-terminally modified RHAU peptides. (**A**) Design of the N-terminally modified peptides. (**B**) CD scans taken of 11 µM peptide in a buffered solution (20 mM Tris HCl, pH 7.4 buffer). (**C**) Peptide-induced thermal stabilization of each N-terminally modified peptide on both parallel (T95-2T, c-Myc, BCL-2) and non-parallel (h-TELO) sequences, derived from UV melting experiments. Melting experiments were performed in a buffered solution [20 mM Tris HCl, pH 7.4 buffer, supplemented with either 10 mM KCl (for BCL-2 and h-TELO) or 25 mM NaCl (for T95-2T and c-Myc)] at 5 µM strand concentration in a 1:2.2 DNA:peptide ratio. (**D**) CD spectra of Kit* and hTel21T18 in 20 mM Tris HCl, pH 7.4 buffer (supplemented with 25 mM KCl), pre-annealed with Nt + His (**12**). The obtained spectra are compared with the signature of the two-quartet chair-type anti-parallel Kit* as such (grey dotted line, left) or the signature of the three-quartet chair-type anti-parallel hTel21T18 (grey dotted line, right). CD experiment performed at 5 µM strand concentration in a 1:2.2 DNA:peptide ratio.

The significant increase in peptide-induced thermal stabilization obtained for most of the tested G4s when introducing the N-terminal furan or benzophenone modification inspired us to perform a more in-depth analysis of the influence of aromatic and/or hydrophobic groups at the N-terminus of RHAU18. Modifications of the RHAU18 N-terminus are, until now, only studied with the aim of introducing a staple [55] and not to optimize the G4-binding. Therefore, we synthesized six additional RHAU18 derivatives by elongating the N-terminus with either the aliphatic Ala (**16**) or the aromatic amino acids Phe (**15**), Trp (**14**), Tyr (**13**), His (**12**), and thiophenyl-alanine (ThioA) (**11**). Tyr (**13**), His (**12**), and ThioA (**11**) were selected for their structural resemblance to furyl-alanine (Fig. 9A), and the resulting peptides displayed a CD spectrum similar to that of peptide **6** (Fig. 9B and Supplementary Fig. S39). The CD signature of each peptide was also measured in the presence of T95-2T. For all six peptides (**11-16**), we notice an increase in helicity content upon binding to the G4 (Supplementary Fig. S40). Importantly, while it was shown that the N-terminus is not involved in the α-helix formation in the unmodified RHAU18 [31], extending the chain clearly impacts the overall folding when the peptide is complexed to the G4, with the exception of peptides **13** (Nt + Tyr) and **15** (Nt + Phe) (Table in Supplementary Fig. S39 versus Table in Supplementary Fig. S40).

### Thermal stabilization and shape-shifting effect of N-terminal modified peptides

All peptides were screened for their impact on the stability and structure of the selected parallel and non-parallel G4 structures. In accordance with the previously obtained data, we observed a G4-dependent stabilization upon peptide binding. Peptides **6** and **12** proved to be the two best-performing ligands in terms of enhancing the thermal stability of G4-DNA (Fig. 9C, Supplementary Figs. S41 and S42, and Supplementary Table S5). Furthermore, the addition of the extra residue to the N-terminus resulted in a larger increase in *T_m_* for most of the tested G4s compared to the unmodified peptide **1**. We believe that additional interactions might occur after functionalization of the N-terminus with the tested amino acids, such as CH-, CH_3_- or π-π-stacking and intermolecular hydrogen bonding with the G4-DNA. Both stacking and hydrogen bonding interactions can take place upon binding of these peptides to the G4s. Peptide **11** conferred the lowest Δ*T_m_*, despite featuring a side chain which is isosteric with **12** and **13**. We argue that the lower electronegativity of the sulphur, compared to the nitrogen and oxygen atoms, makes it a poor H-bond acceptor [56], thus reducing its effectiveness in stabilizing the G4 structures.

Next, we investigated the conformational transition of h-TELO upon binding of the newly synthesized peptides. First, the conversion from hybrid to parallel h-TELO was studied. In analogy to peptides **2** and **6**, all tested N-term modifications were able to induce a (partial) conformational shift. Interestingly, peptide **12** caused the transition of the anti-parallel h-TELO to the parallel fold (Supplementary Fig. S43), which was not observed for peptide **6**. Since we performed the experiment at physiological pH (∼7.4), we reasoned that the protonation state of the His side chain could be the reason for this unique difference observed in the binding to the anti-parallel h-TELO. Additionally, peptide **12** demonstrated the universal ability to shift the G4 conformation, as it also converted the anti-parallel chair-type G4s, Kit* and hTel21T18T, to predominantly parallel structures (Fig. 9D).

### Investigation of ‘hit’ peptides by ITC, CD, and NMR experiments

**Figure 10.**
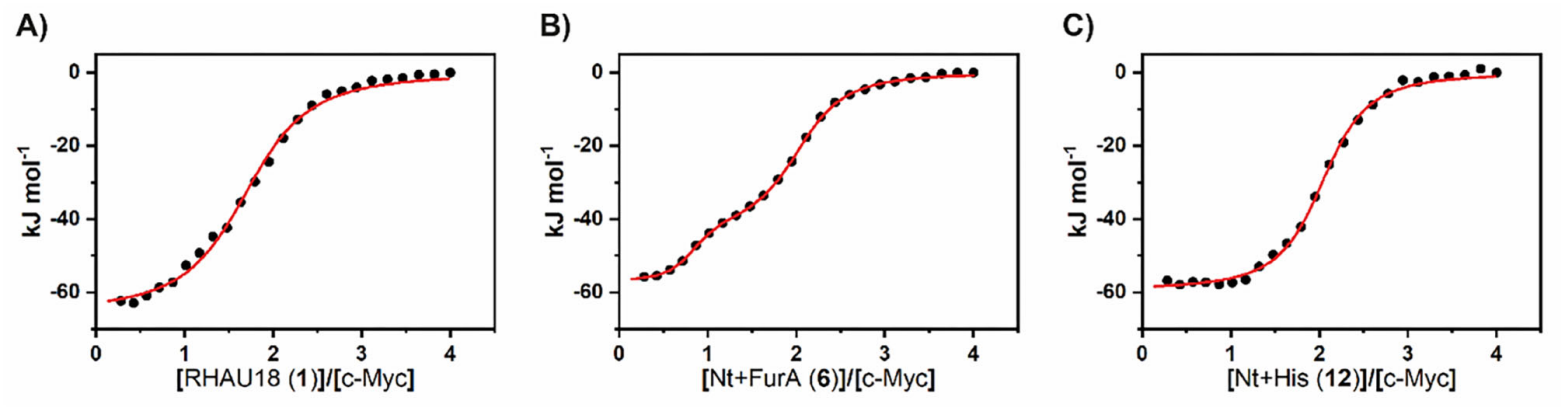
Binding isotherms for titration of c-Myc G4 with (**A**) RHAU18 (**1**), (**B**) Nt + FurA (**6**), and (**C**) Nt + His (**12**) peptides obtained at 25°C in 5 mM KH_2_PO_4_/K_2_HPO_4_ buffer containing 20 mM KCl (pH 7.0). The black dots represent the experimental data obtained by integrating the raw ITC data and subtracting the heat of peptide dilution into the buffer. The red lines represent the best-fit curve for the data.

Peptides **6** and **12** were selected as ‘hit’ peptides due to their general and significant increase in *T_m_* values of G4s and were further analysed by ITC and NMR to gain deeper insight into their interaction with G4-forming DNA. For this study, c-Myc was selected as the target, as it showed the highest stabilization among the investigated G4s. ITC experiments were performed to characterize the thermodynamics of peptide binding to G4. Indeed, this is the only technique capable of quantifying both the enthalpic and entropic contributions to the binding affinity, thereby revealing the driving forces of the molecular recognition [57]. Control experiments were also performed to evaluate the heat of peptide dilution, i.e. the heat associated with injection of ligand solution into the buffer (Supplementary Fig. S44). Raw ITC data (Supplementary Fig. S44) and binding isotherms (Fig. 10) for c-Myc titration with peptides **6, 12**, and the reference RHAU18 peptide **1** revealed exothermic binding processes. ITC data analysis indicated enthalpically driven interactions for all peptides (Δ*H*° < 0) with unfavourable entropic contributions (*T*Δ*S*° < 0) (Table 1). Given the absence of conformational changes for c-Myc G4 upon binding (*vide infra*), this suggests that the peptides may undergo conformational changes upon interaction. The overall stoichiometry for the binding of **1, 6**, and **12** to c-Myc was determined to be two peptides per G4-DNA. However, binding isotherms for peptides **1** and **12** were characterized by a single binding event, while ITC titration for peptide **6** resulted in an isotherm revealing a two-event binding process, each involving one peptide molecule. In particular, the binding isotherm for peptide **6** exhibited the typical profile of a system where the binding site with higher affinity is accompanied by a more exothermic enthalpy change. Indeed, data analysis revealed that the first event (*K*_a_ = 2.3 × 10^7^ M^−1^) is ~30-fold stronger than the second (*K*_a_ = 6.7 × 10^5^ M^−1^). Estimated equilibrium binding constants for peptides **1** and **12** were 6.0 × 10^5^ M^−1^ and 1.3 × 10^6^ M^−1^, respectively. Overall, in agreement with thermal stabilization data, the ITC results indicate that **6** and **12** interact with c-Myc G4 with higher affinity than **1**, albeit with different thermodynamic signatures.

**Table 1.** Thermodynamic parameters for the non-covalent binding of the peptides to c-Myc G4, obtained by ITC at 25°C

Peptide	No. of events	*n*	*K*_a_ (M^−1^)	$\Delta $ *G*° (kJ mol^−1^)	$\Delta $ *H*° (kJ mol^−1^)	*T* $\Delta $ *S*° (kJ mol^−1^)
**1**	1	2	6.0 (±0.7) × 10^5^	−33.0	−61.3	−28.3
**6**	2	1	2.3 (±0.7) × 10^7^	−42.0	−56.2	−14.2
		1	6.7 (±0.9) × 10^5^	−33.2	−39.2	−6.0
**12**	1	2	1.3 (±0.1) × 10^6^	−34.9	−59.1	−24.2

To evaluate any secondary structure changes in the peptides upon G4 binding, CD spectra were recorded for peptides **1, 6**, and **12**, the c-Myc G4, and their respective mixtures at a 2:1 peptide/G4 molar ratio, based on the stoichiometry determined by ITC. As shown in Supplementary Fig. S45, peptide binding does not perturb the conformation of the c-Myc G4. Indeed, the spectra retain the characteristic features of a parallel G4 (namely, a positive band at 264 nm and a negative band at 245 nm), with only minor intensity variations, indicating that the G4 topology remains unaffected in the presence of the peptides. To assess peptide-specific conformational changes, the CD spectrum of the free c-Myc G4 was subtracted from those of the G4/peptide mixtures. The resulting difference spectra revealed clear changes in the 200–220 nm region (Supplementary Fig. S46), where peptide secondary structure signatures are typically observed. These changes, observed for all three peptides, suggest conformational rearrangements upon G4 binding. Small changes were also observed in the 230–250 nm region but overlapping DNA absorption in this range prevents unambiguous assignment to the peptide. Notably, BeStSel analysis of the difference spectra (Supplementary Table S6) revealed a significant increase in α-helical content, confirming that the interaction with the G4 promotes peptide folding into an α-helical conformation. This behaviour is consistent with previous reports [23, 36], supporting the idea that G4 structures can act as molecular scaffolds inducing peptide folding.

Finally, to investigate the binding mode of selected peptides to the c-Myc G4, one-dimensional proton (1D ¹H) NMR experiments were performed. According to the literature, the free c-Myc G4 exhibits 12 peaks in the imino proton region (10.5–12 ppm), each corresponding to one of the 12 guanines involved in the three G-tetrad planes of the G4 structure [58]. Upon the addition of 0.5, 1.0, and 1.5 equivalents of peptides **1, 6**, and **12**, broadening of all imino proton signals was observed (Supplementary Fig. S47), confirming peptide interaction. At a G4/peptide ratio of 1:2, a new set of downfield-shifted imino proton peaks emerged, indicating the formation of G4/peptide complexes, while signals from free G4 were no longer detected. The titration was complete at this ratio, consistent with the stoichiometry determined by ITC. Indeed, further increasing the peptide concentration to G4/peptide ratios of 1:3 and 1:4 did not alter imino proton signals, suggesting saturation of binding sites. However, signals around 10.0–10.2 ppm, attributable to excess free peptides, were detected, as confirmed by comparison with 1D ¹H NMR spectra of peptides recorded at the same concentrations (Supplementary Fig. S47). Notably, these signals appeared even at lower G4/peptide ratios for peptide **1**, consistent with its lower binding affinity determined by ITC. Overall, these findings suggest that all three peptides exhibit similar binding modes and that both external G-tetrads of the c-Myc G4 are involved in peptide binding.

## Conclusion

In this study, we exploited the well-defined G4-binding motif of the RHAU helicase as an efficient and selective G4-binder for the development of a peptide-based covalent G4-targeting methodology. By leveraging the modularity of the peptide scaffold, furyl-alanine or benzoylphenyl-alanine were strategically introduced at specific locations within the sequence. Photo-uncaging of the masked warheads, furan and benzophenone, resulted in moderate to high alkylation yields for most of the peptides tested. All modified peptides demonstrated high selectivity for G4 over dsDNA, both in terms of stabilization and crosslinking ability. Besides the efficient alkylation, all modified peptides showed G4 stabilizing effects, with a significant enhancement for the N-terminal modifications. Screening of different aliphatic and hydrophobic N-terminal modifications led to the identification of two ‘hit’ peptides **6** and **12**, which were further characterized by means of ITC and NMR to get insight into their binding mode. These studies confirmed the 1:2 binding stoichiometry of the modified RHAU peptides to their G4 target. Moreover, they revealed that incorporating a single amino acid modification in the non-helical N-terminal part of the peptide significantly increases the binding affinity, yielding a series of versatile G4-binding ligands. In conclusion, we present a versatile chemical toolbox of peptide-based ligands for multiple purposes, ranging from crosslinking to stabilization and shape-shifting of G4s, thus expanding the current library of G4-targeting ligands and extending the alkylation approaches from small molecules to peptides.

## Supplementary Material

gkag039_Supplemental_File

## Data Availability

The data underlying this article are available in the article and is online in the supplementary materials. Raw data will be shared on reasonable request to the corresponding author.
