# Supplementary material for: From alkylating to shape-shifting G-quadruplex ligands: the RHAU peptide story
Source: Nucleic Acids Res. 2026 Jan 29;54(3):gkag039. doi: 10.1093/nar/gkag039 (PMC12852953; doi:10.1093/nar/gkag039)
Supplement: gkag039_Supplemental_File [file gkag039_supplemental_file.pdf]

## SUPPORTING INFORMATION

# From Alkylating to Shape-Shifting G-Quadruplex ligands: The RHAU Peptide Story

Lessandro De Paepe[1], Simona Marzano[2], Camille Vesschemoet[1], Jussara Amato[2,*], Bruno Pagano[2], Enrico Cadoni[1,*], Annemieke Madder[1,*]

*[1]Department of Organic and Macromolecular Chemistry, Organic and Biomimetic Chemistry Research Group, Ghent University, Krijgslaan 281, Building S4, B-9000, Gent, Belgium*
*[2]Department of Pharmacy, University of Naples Federico II, via D. Montesano 49, 80131 Naples, Italy*

* To whom correspondence should be addressed. Tel: +32 92644472; Fax: +32 9244998; Email: annemieke.madder@ugent.be
Correspondence may also be addressed to Jussara Amato. Email: jussara.amato@unina.it
Correspondence may also be addressed to Enrico Cadoni. Email: enrico.cadoni@ugent.be

## Table of contents

1. **General information**
2. **Peptide synthesis**
3. **Alkylation experiments**
4. **UV-melting experiments with first set of peptides**
5. **CD conformational shift experiments with FurA- and BPA-peptides**
6. **Alkylation experiments by co-annealing RHAU peptides with h-TELO**
7. **CD characterisation of N-terminal modified peptides**
8. **UV melting experiments with extended set of N-terminal modified peptides**
9. **CD conformational shift experiments with N-terminal modified peptides**
10. **ITC experiments**
11. **Structural analysis of peptides upon c-Myc G4-binding**
12. **NMR experiments**
13. **Appendix. HPLC-UV stability check & HPLC-MS characterisation of peptides**

## 1. General information

All amino acids were purchased from Iris Biotech Gmbh and TCI Europe. All the other reagents for peptide synthesis were purchased from Sigma-Aldrich, Fluorochem and TCI Europe. DNA sequences were ordered from Integrated DNA technologies (Leuven, Belgium) and are depicted in Table S2.

**HPLC-MS** data were collected on an Agilent 1100 Series instrument equipped with a Phenomenex Kinetex C18 100 Å column (150 x 4.6 mm, 5 µm at 35°C) connected to an ESMSD type VL mass detector (quadrupole ion trap mass spectrometer) with a flow rate of 1.5 ml/min was used with the following solvent system: (A): 0.1% HCOOH in $H_2O$ and (B) MeCN. Gradient: 100% A for 2 min, then a gradient from 0 to 100% B over 6 min was used, followed by 2 min of flushing with 100% B (further referred as **HPLC-1** conditions).

**Orbitrap-MS** data were generated on a Thermo Fisher Scientific Q Exactive Plus Orbitrap Mass Spectrometer.

**MALDI-TOF** analysis was performed using an Applied Biosystems - 4800 Plus MALDI TOF/TOF™ Analyzer. As matrix, either 100 mg/mL 2,5-Dihydroxybenzoic acid (DHB) in mQ water : MeCN (1:2) + 0.1% TFA was used, or a saturated solution of α-cyano-4-hydroxycinnamic acid (α-CHCA) in MeCN : 0.1% TFA, 1:2).

**HPLC-UV** data were collected on an Agilent 1100 or Agilent 1200 Series Instrument connected to a DAD. The peptides were purified on the Agilent 1200 equipped with a Luna C18 (5 µm, 100 Å, 250x10 mm) column, with the following solvent system: (A): 0.1% TFA in $H_2O$ and (B) MeCN. Gradient: 100% A for 5 min, then a gradient from 0 to 100% B over 30 min at a flow rate of 4.0 mL/min (**HPLC-2 conditions**). Peptide activation experiments were conducted on an Agilent 1200 equipped with a Narrowbore jupiter or Kinetex C18 300 Å column (150 x 2 mm, 5 µm at 50°C), with the following solvent system: (A) 0.1% TFA in $H_2O$ and (B) MeCN. Gradient: 100% A for 4 min, then a gradient from 0 to 40% over 12 min at a flow rate of 0.350 mL/min (**HPLC-3 conditions**). The alkylation experiments were analysed on Agilent 1100 equipped with a Waters X-Bridge BEH C18 XP Column (130Å, 2.5 µm, 4.6 mm X 50 mm) using a flow rate of 0.80 mL/min with the following solvent system: (A) 0.1 M TEAA-buffer + 5% MeCN and (B) MeCN. Column was flushed for 4 minutes with (A), then a gradient from 0 to 50% B in 12 minutes was used, followed by 4 minutes flush of 100% B (**HPLC-4 conditions**).

**CD** spectra were recorded at room temperature on an AVIV model 410 instrument (Biomedical, Inc, Lakewood NJ USA) equipped with a Thermo Scientific temperature-control, using quartz cuvettes of 250 µL with a 1 mm pathlength or quartz cuvettes of 350 µL with a 0.1 mm pathlength.

**Alkylation experiments** were performed in an Eppendorf Thermomixer Comfort for temperature control using for visible light irradiation: Euromex Illuminator EK-1 lamps, equipped with a 100 W halogen lamp LE.5210 and connected to an Euromex LE.5214 dual arm light conductor, with green-light filter (Euromex LE.5223) was used. Prior to each experiment, the power of the lamps was measured using a TES 1335 light meter equipped with a custom fitting for the dual-arms and adjusted to 7-9 kLux. A benchtop 2UV Transilluminator (UVP; Cambridge, UK) with 302/365 nm UV lamp (8 Watt, 0.2 Amps, 220-230V and 50-60 Hz) was used for UVA irradiation experiments.

**UV melting** experiments were recorded on a Varian Cary 300 Bio instrument equipped with a six-cell thermostatic cell holder.

**ITC experiments** were recorded on a nano-ITC Low Volume calorimeter (TA instruments, Lindon, UT, USA).

**NMR** spectra were recorded on a 600 MHz Bruker Avance NEO NMR spectrometer (Bruker Biospin, Rheinstetten, Germany) equipped with a 5-mm QCI cryo-probe and a cooled SampleJet autosampler.

## 2. Peptide synthesis

**Table S1**: Overview of the synthesized peptides, written from N- to C-terminus. FurA = L-furyl-alanine and BPA = L-benzoyl-phenylalanine.

| Peptide (**number**) | Sequence (N-to C-terminus) |
|---|---|
| RHAU18 (**1**) | Ac-HPGHLKGREIGMWYAKKQ-$NH_2$ |
| Nt+BPA (**2**) | Ac-**BPA**-HPGHLKGREIGMWYAKKQ-$NH_2$ |
| K6:BPA (**3**) | Ac-HPGHL-**BPA**-GREIGMWYAKKQ-$NH_2$ |
| K6+BPA (**4**) | Ac-HPGHLK(-**BPA**-)GREIGMWYAKKQ-$NH_2$ |
| Y14:BPA (**5**) | Ac-HPGHLKGREIGMW-**BPA**-AKKQ-$NH_2$ |
| Nt-FurA (**6**) | Ac-**FurA**-HPGHLKGREIGMWYAKKQ-$NH_2$ |
| H1:FurA (**7**) | Ac-**FurA**-PGHLKGREIGMWYAKKQ-$NH_2$ |
| K6:FurA (**8**) | Ac-HPGHL-**FurA**-GREIGMWYAKKQ-$NH_2$ |
| K6+FurA (**9**) | Ac-HPGHLK(-**FurA**-)GREIGMWYAKKQ-$NH_2$ |
| Y14:FurA (**10**) | Ac-HPGHLKGREIGMW-**FurA**-AKKQ-$NH_2$ |
| Nt+ThioA (**11**) | Ac-**ThioA**-HPGHLKGREIGMWYAKKQ-$NH_2$ |
| Nt+His (**12**) | Ac-**H**HPGHLKGREIGMWYAKKQ-$NH_2$ |
| Nt+Tyr (**13**) | Ac-**Y**HPGHLKGREIGMWYAKKQ-$NH_2$ |
| Nt+Trp (**14**) | Ac-**W**HPGHLKGREIGMWYAKKQ-$NH_2$ |
| Nt+Phe (**15**) | Ac-**F**HPGHLKGREIGMWYAKKQ-$NH_2$ |
| Nt+Ala (**16**) | Ac-**A**HPGHLKGREIGMWYAKKQ-$NH_2$ |

**RHAU18 (1):** 9.70%; rt = 3.22 min; ε $_{(\lambda=280nm)}$= 6970 $M^{-1}cm^{-1}$; ESI-MS: m/z calc: 2176 m/z found: 1089.2 $[M+2H]^{2+}$, 726.5 $[M+3H]^{3+}$, 545.1 $[M+4H]^{4+}$, 436.4 $[M+5H]^{5+}$, 363.9 $[M+6H]^{6+}$. **Nt+BPA (2)**: 15.48%; rt = 3.82 min; ε $_{(\lambda=280nm)}$= 10520 $M^{-1}cm^{-1}$; ESI-MS: m/z calc: 2428 m/z found: 1214.8 $[M+2H]^{2+}$, 810.3 $[M+3H]^{3+}$, 608.0 $[M+4H]^{4+}$, 486.6 $[M+5H]^{5+}$, 405.8 $[M+6H]^{6+}$. **K6:BPA (3)**: 13.47%; rt = 3.73 min; ε $_{(\lambda=280nm)}$= 10520 $M^{-1}cm^{-1}$; ESI-MS: m/z calc: 2300 m/z found: 1150.8 $[M+2H]^{2+}$, 767.5 $[M+3H]^{3+}$, 576.0 $[M+4H]^{4+}$, 461.0 $[M+5H]^{5+}$. **K6+BPA (4)**: 9.49%; rt = 3.72 min; ε $_{(\lambda=280nm)}$= 10520 $M^{-1}cm^{-1}$; ESI-MS: m/z calc: 2470 m/z found: 1235.8 $[M+2H]^{2+}$, 824.3 $[M+3H]^{3+}$, 618.5 $[M+4H]^{4+}$, 495.0 $[M+5H]^{5+}$. **Y14:BPA (5)**: 11.73%; rt = 3.42 min; ε $_{(\lambda=280nm)}$= 9240 $M^{-1}cm^{-1}$; ESI-MS: m/z calc: 2265 m/z found: 755.8 $[M+3H]^{3+}$, 567.2 $[M+4H]^{4+}$, 454.0 $[M+5H]^{5+}$, 378.5 $[M+6H]^{6+}$. **Nt+FurA (6)**: 10.27%; rt = 3.29 min; ε $_{(\lambda=280nm)}$= 6970 $M^{-1}cm^{-1}$; ESI-MS: m/z calc: 2316 m/z found: 1157.7 $[M+2H]^{2+}$, 772.2 $[M+3H]^{3+}$, 579.5 $[M+4H]^{4+}$, 463.8 $[M+5H]^{5+}$, 386.7 $[M+6H]^{6+}$. **H1:FurA (7)**: 5.65% rt = 3.47 min; ε $_{(\lambda=280nm)}$= 6970 $M^{-1}cm^{-1}$; ESI-MS: m/z calc: 2176 m/z found: 1089.3 $[M+2H]^{2+}$, 726.5 $[M+3H]^{3+}$, 545.1 $[M+4H]^{4+}$, 436.4 $[M+5H]^{5+}$. **K6:FurA (8)**: 5.21% rt = 3.55 min; ε $_{(\lambda=280nm)}$= 6970 $M^{-1}cm^{-1}$; ESI-MS: m/z calc: 2185 m/z found: 1093.8 $[M+2H]^{2+}$, 729.8 $[M+3H]^{3+}$, 547.3 $[M+4H]^{4+}$, 438.1$[M+5H]^{5+}$. **K6+FurA (9)**: 2.75%; rt = 3.50 min ; ε $_{(\lambda=280nm)}$= 6970 $M^{-1}cm^{-1}$; ESI-MS: m/z calc: 2356 m/z found: 786.2 $[M+3H]^{3+}$, 590.0 $[M+4H]^{4+}$, 472.2 $[M+5H]^{5+}$. **Y14:FurA (10)**: 9.82%; rt = 3.28 min; ε $_{(\lambda=280nm)}$= 5690 $M^{-1}cm^{-1}$; ESI-MS: m/z calc: 2151 m/z found: 1076.3 $[M+2H]^{2+}$, 717.9 $[M+3H]^{3+}$, 538.7 $[M+4H]^{4+}$, 431.3 $[M+5H]^{5+}$. **Nt+ThioA (11)**: 8.49%; rt = 3.36 min; ε $_{(\lambda=280nm)}$= 6970 $M^{-1}cm^{-1}$; ESI-MS: m/z calc: 2331 m/z found: 1165.8 $[M+2H]^{2+}$, 777.6 $[M+3H]^{3+}$, 583.5 $[M+4H]^{4+}$, 467.0 $[M+5H]^{5+}$, 389.4 $[M+6H]^{6+}$. **Nt+His (12)**: 6.01%; rt = 3.14 min; ε $_{(\lambda=280nm)}$= 6970 $M^{-1}cm^{-1}$; ESI-MS: m/z calc: 2316 m/z found: 1157.8 $[M+2H]^{2+}$, 772.3 $[M+3H]^{3+}$, 579.5 $[M+4H]^{4+}$, 463.8 $[M+5H]^{5+}$, 386.8$[M+6H]^{6+}$, 331.6$[M+7H]^{7+}$. **Nt+Tyr (13)**: 5.70%; rt = 3.30 min; ε $_{(\lambda=280nm)}$= 8250 $M^{-1}cm^{-1}$; ESI-MS: m/z calc m/z: 2342 found: 1170.9 $[M+2H]^{2+}$, 780.9 $[M+3H]^{3+}$, 586.0 $[M+4H]^{4+}$, 469.0 $[M+5H]^{5+}$, 391.0 $[M+6H]^{6+}$. **Nt-Trp (14)**: 8.10%; rt = 3.44 min; ε $_{(\lambda=280nm)}$= 12660$M^{-1}cm^{-1}$; ESI-MS: m/z calc: 2364 m/z found: 1182.3 $[M+2H]^{2+}$, 788.5 $[M+3H]^{3+}$, 591.8 $[M+4H]^{4+}$, 473.5 $[M+5H]^{5+}$. **Nt-Phe (15)**: 11.01%; rt = 3.41min; ε $_{(\lambda=280n)}$= 6970 $M^{-1}cm^{-1}$; ESI-MS: m/z calc: 2325 m/z found: 1162.8 $[M+2H]^{2+}$, 775.7 $[M+3H]^{3+}$, 582.0 $[M+4H]^{4+}$, 465.8 $[M+5H]^{5+}$, 388.4 $[M+6H]^{6+}$. **Nt+Ala (16)**: 8.51%; rt = 3.24 min; ε $_{(\lambda=280nm)}$= 6970 $M^{-1}cm^{-1}$; ESI-MS: m/z calc: 2249 m/z found: 1124.8 $[M+2H]^{2+}$, 750.2 $[M+3H]^{3+}$, 563.0 $[M+4H]^{4+}$, 450.6 $[M+5H]^{5+}$, 375.6 $[M+6H]^{6+}$.

## 3. Alkylation experiments

**Table S2**: Overview of the used DNA sequences, written from 5'- to 3'-end. [1.] Topology depends on the used cation. In this work, we focused on the hybrid h-TELO (formed in $K^+$) and the basket h-TELO (formed in $Na^+$).

| DNA | Sequence (5'-to 3'-end) | Description |
|---|---|---|
| T95-2T | TTG GGT GGG TGG GTG GGT | Parallel G4 |
| c-Myc | TGA GGG TGG GTA GGG TGG GTA A | Parallel G4 |
| c-Kit2 | CGG GCG GGC GCG AGG GAG GGT | Parallel G4 |
| BCL-2 | AGGGGCGGGCGCGGGAGGAAGGGGGCGGGAG | Parallel G4 |
| VEGF | GGGCGGGCCGGGGGCGGG | Parallel G4 |
| c-Kit2-FAM | FAM-CGG GCG GGC GCG AGG GAG GGT | Parallel G4 |
| T95-2T-FAM | FAM-TTG GGT GGG TGG GTG GGT | Parallel G4 |
| h-TELO | A GGG TTA GGG TTA GGG TTA GGG | Non-parallel G4[1] |
| Kit* | GGCGAGGAGGGGCGTGGCCGGC | Antiparallel chair G4 |
| hTel21T18 | GGGTTAGGGTTAGGGTTTGGG | Anti-parallel chair G4 |
| dsLAC | GAATTGTGAGCGCTCACAATTC | Self-complementary ssDNA |
| ssT3 | TTACCGTGACAC | ssDNA |
| DsDNA | CAATCGGATCGAATTCGATCCGATTG GTTAGCCTAGCTTAAGCTAGGCTAAC | dsDNA |

### Oxidation kinetics peptides:

In a typical experiment, a 200 µL peptide, solution (12.5 µM) was prepared in a 20 mM phosphate ($K_2HPO_4$) pH 7 buffer. K6:FurA (**peptide 8**), selected as representative for the furan-containing peptides, was irradiated with green light for 5h in presence of 20 µM RhoB. K6:BPA (**peptide 3**), selected as representative for the BPA-containing peptides, was irradiated with UVA-light for 10 min. Intermediate samples were taken, shielded from light and analysed using **HPLC-3 conditions**.

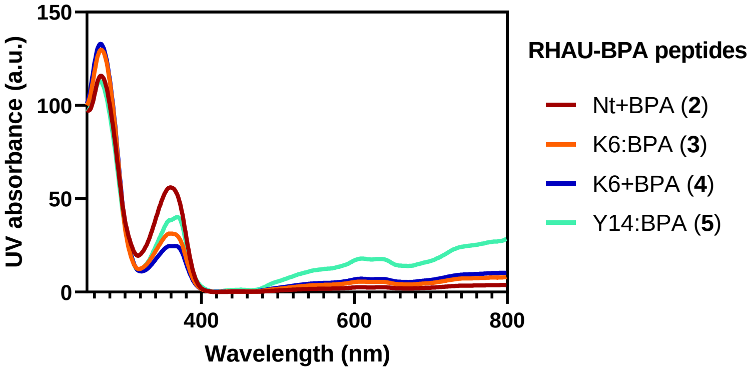


**Figure S1**. UV absorbance spectra of RHAU-BPA peptides: Nt+BPA (**2**), K6:BPA (**3**), K6+BPA (**4**) and Y14:BPA (**5**). Experiment performed at 150 µM peptide concentration.

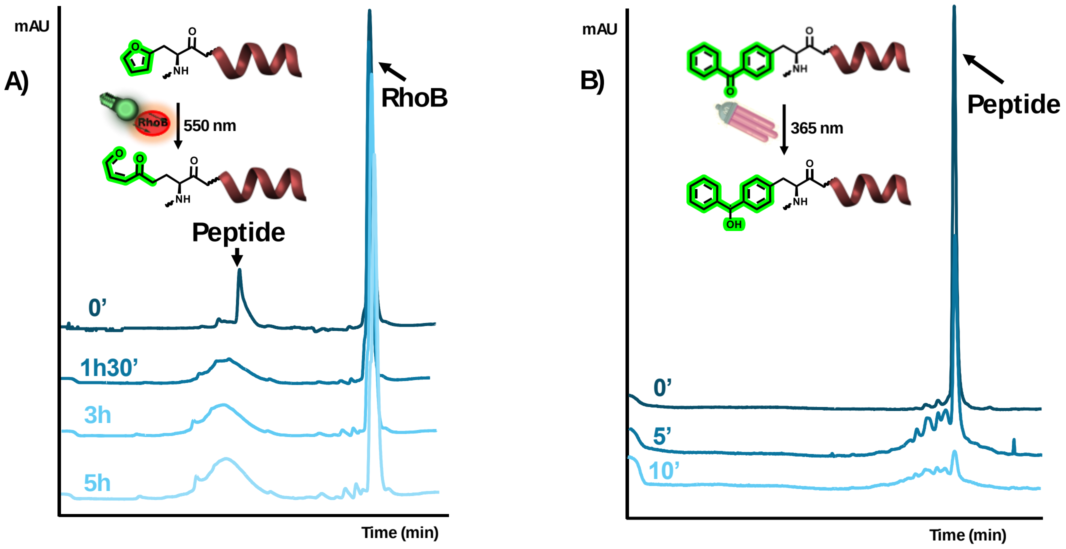


**Figure S2**. Evaluation of the activation kinetics of both alkylating peptide series. A) HPLC trace of K6:FurA (**8**) upon green light irradiation (λ=550 nm) in presence of Rhodamine B for 5h. B) HPLC trace of K6:BPA (**3**) upon UVA-light irradiation (λ=365 nm) for 10 min. Analyses were conducted under **HPLC-3 conditions**.

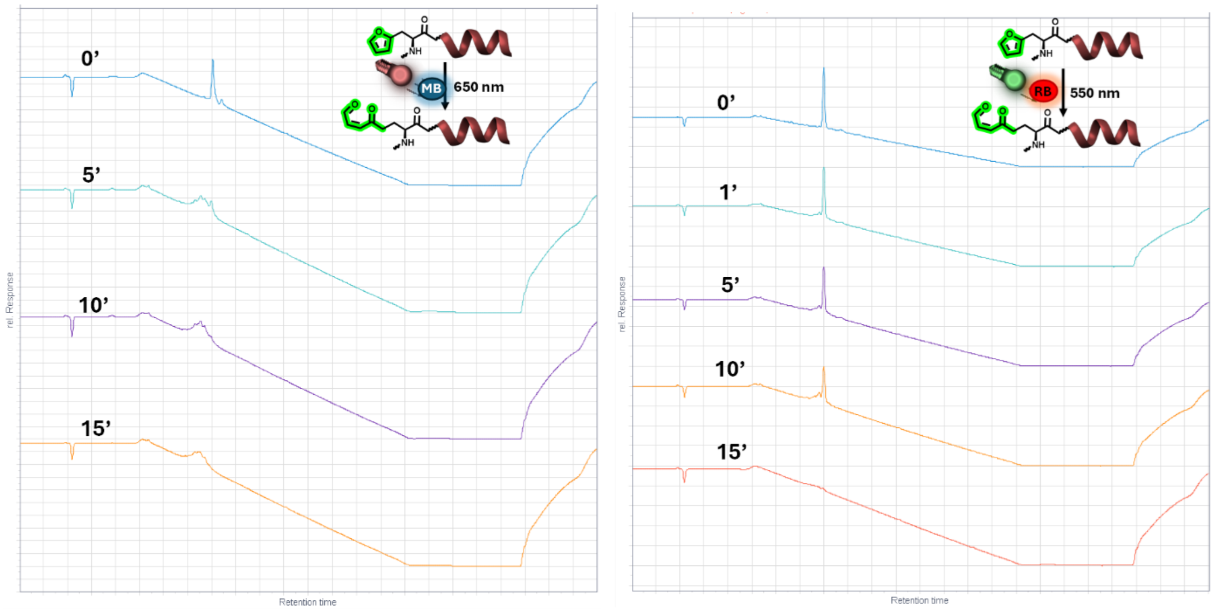


**Figure S3**. Evaluation of the activation kinetics of K6:FurA (**8**) upon either red light irradiation (λ=650 nm) in presence of Methylene Blue for a time period of 15 min (left) or green light irradiation (λ=550 nm) in presence of Rose Bengal for 15 min. Analyses were conducted under **HPLC-3 conditions**.

## Alkylation experiment FurA-peptides:

In a typical experiment, a 50 µL solution of 5 µM DNA was prepared in a 20 mM phosphate ($K_2HPO_4$) pH 7 buffer and pre-annealed by heating for 5 min at 95°C, followed by a cooling down period for 4h. 2.5 equivalents of FurA-peptide were added to the solution, equilibrated for 1 h and subjected to the 1 h 30 minutes of green light in presence of 20 µM RhoB. Prior irradiation, a blank was taken. Finally, the samples were analysed using **HPLC-4 conditions**. Under these conditions, peptides can in most cases not be visualised and indeed elute at longer retention times (Rt around 12 minutes).

The peak eluting later than 15 minutes corresponds to RhoB, which is typically not (completely) consumed. In contrast, the peptide peaks, when visible, are fully consumed in all HPLC spectra. In such cases, the peak around 12 minutes either disappears entirely or broadens and elutes at slightly shorter retention times.

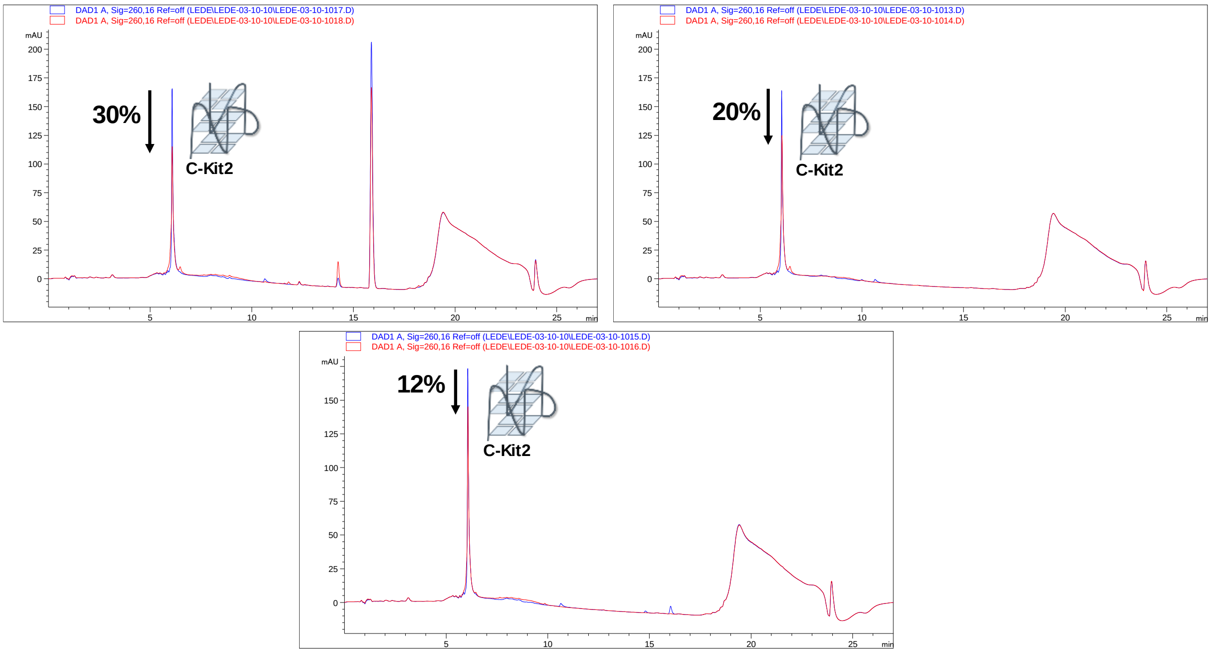


**Figure S4**. HPLC alkylation experiments of parallel c-Kit2 with K6:FurA (**8**) using 20 µM RhoB (left), 1 µM MB (right) or 1 µM RB (middle). Blue trace = before irradiation, red trace = after 1 h 30 (for RhoB) or 20’ irradiation (for MB and RB). Analyses were conducted under **HPLC-4 conditions**.

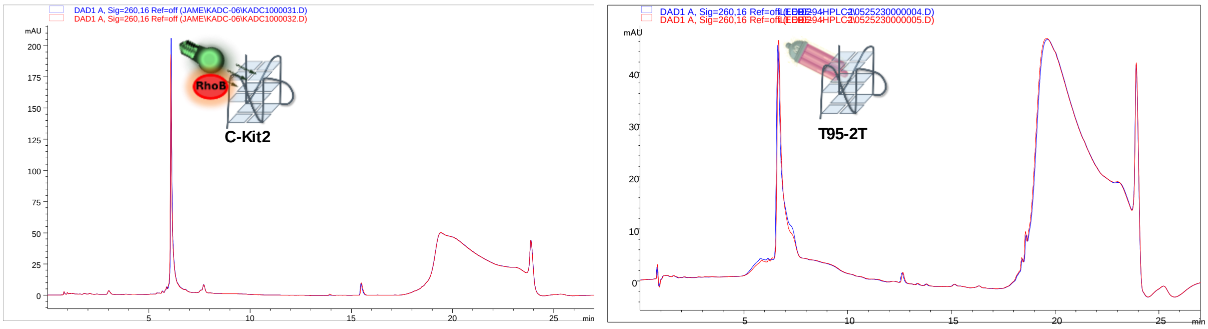


**Figure S5**. Control experiment: HPLC traces of c-Kit2 exposed to 1h30’ light irradiation in presence of Rhodamine B (left) and HLPC traces of T95-2T exposed to 10’ UVA light irradiation (right). Blue trace = before irradiation, red trace = after irradiation. Analyses were conducted under **HPLC-4 conditions**.

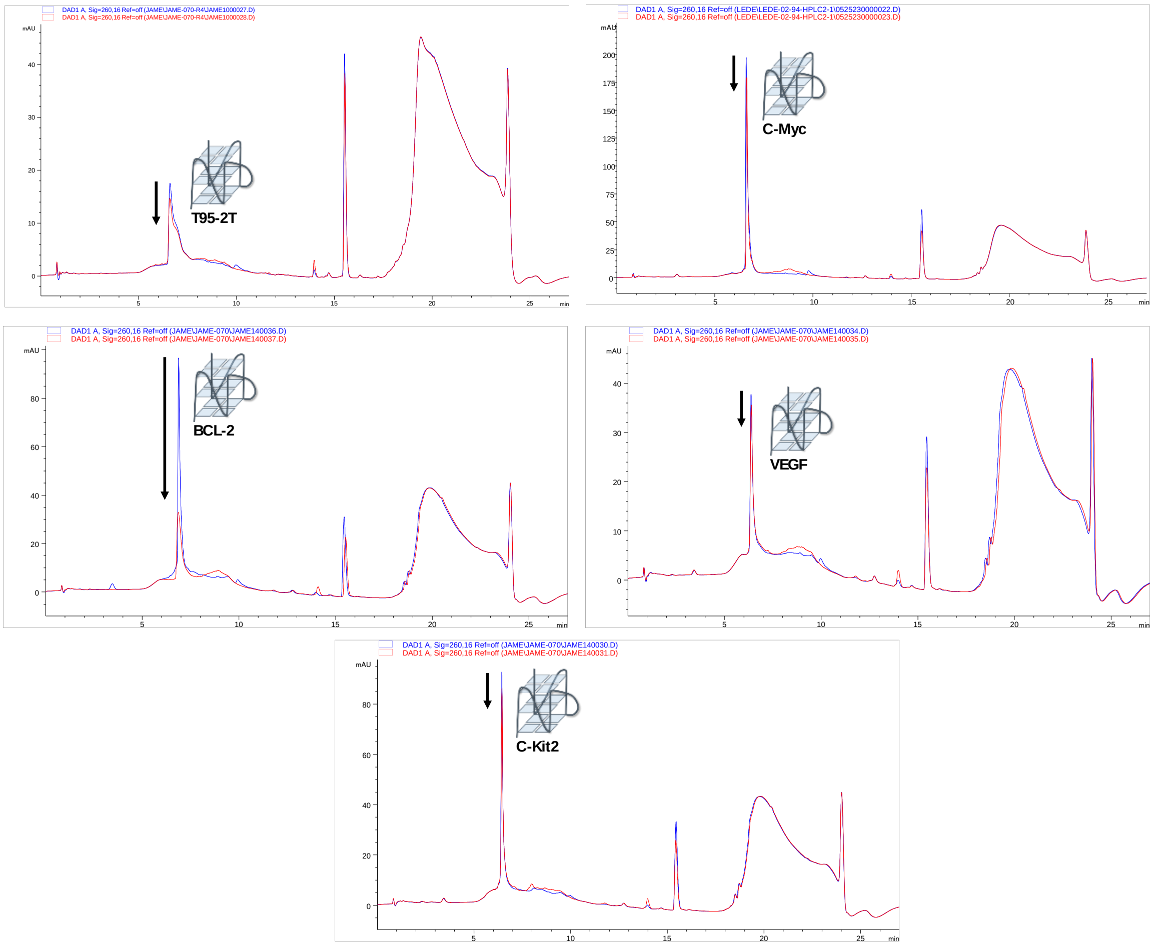


**Figure S6**. HPLC alkylation experiment of parallel G4 sequences (T95-2T, c-Myc, BCL-2, VEGF and c-Kit2) with Nt+FurA (**6**). Blue trace = before irradiation, red trace = after 1 h 30’ irradiation. Analyses were conducted under **HPLC-4 conditions**.

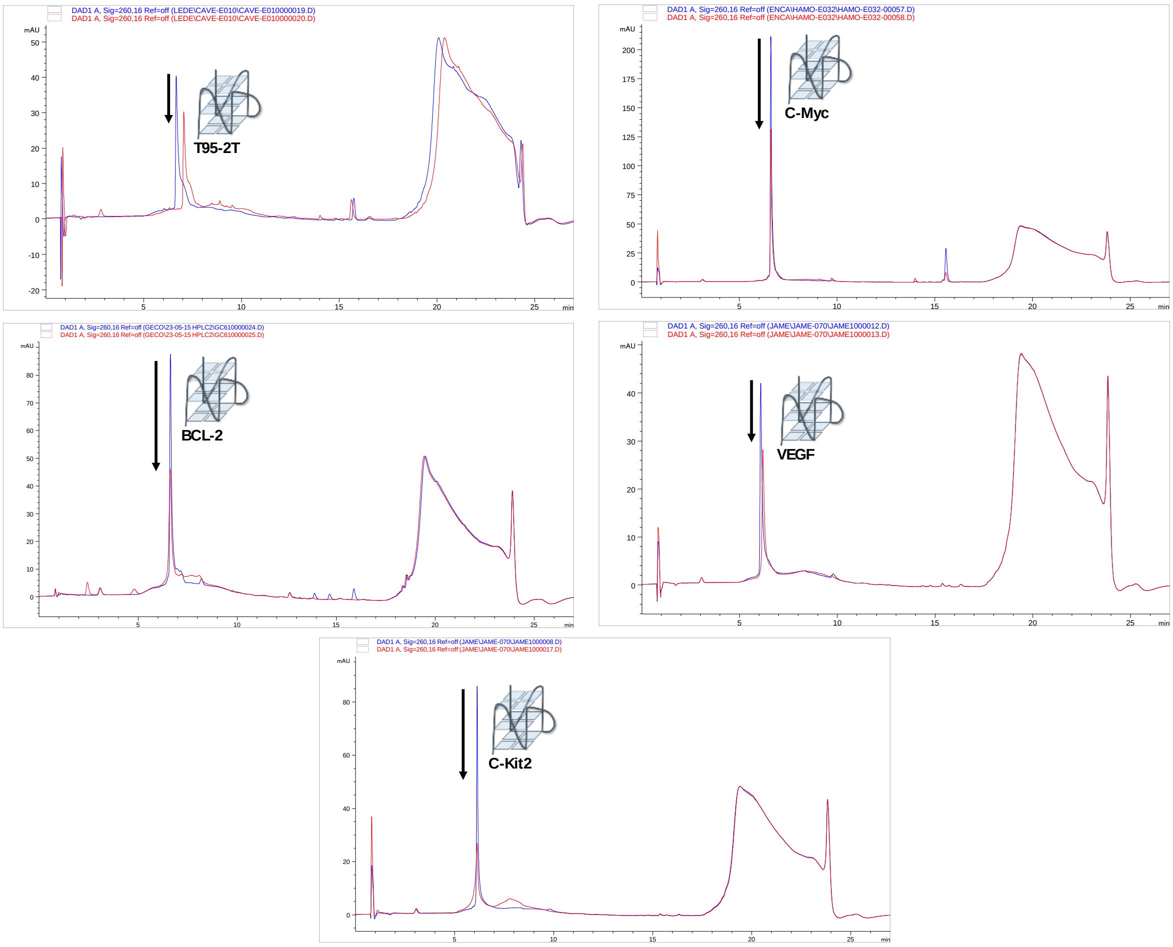


**Figure S7**. HPLC alkylation experiment of parallel G4 sequences (T95-2T, c-Myc, BCL-2, VEGF and c-Kit2) with H1:FurA (**7**). Blue trace = before irradiation, red trace = after 1 h 30’ irradiation. Analyses were conducted under **HPLC-4 conditions**.

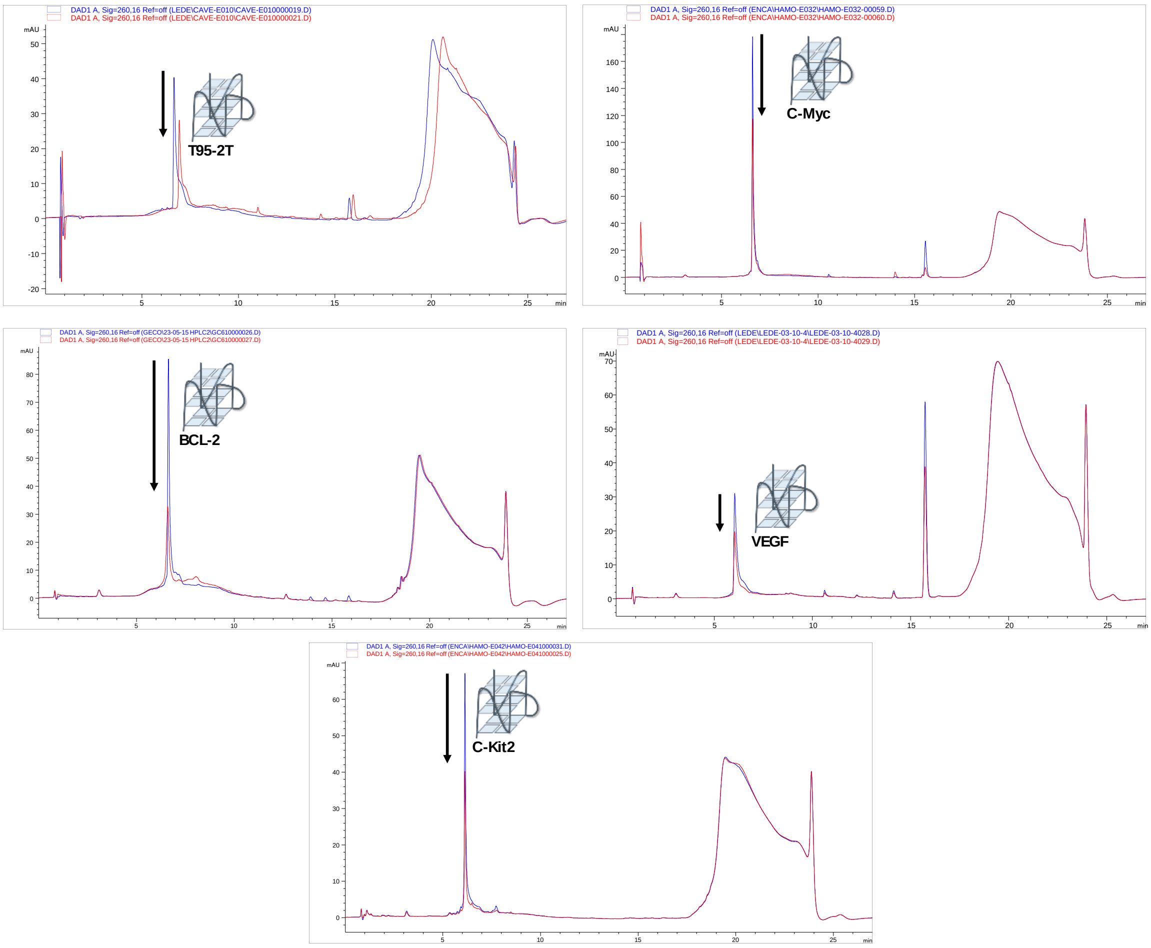


**Figure S8**. HPLC alkylation experiment of parallel G4 sequences (T95-2T, c-Myc, BCL-2, VEGF, c-Kit2) with K6:FurA (**8**). Blue trace = before irradiation, red trace = after 1 h 30' irradiation. Analyses were conducted under **HPLC-4 conditions**.

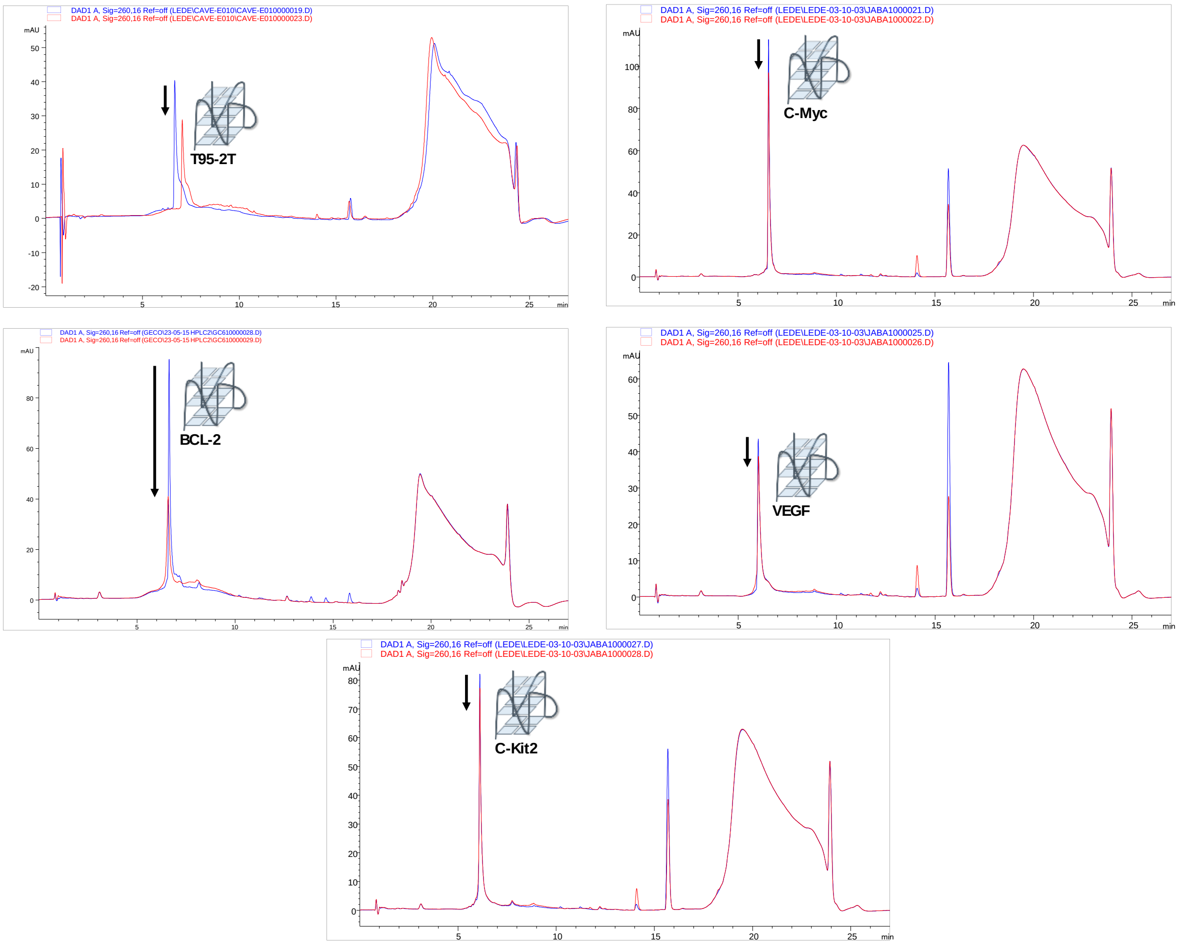


**Figure S9**. HPLC alkylation experiment of parallel G4 sequences (T95-2T, c-Myc, BCL-2, VEGF, c-Kit2) with K6+FurA (**9**). Blue trace = before irradiation, red trace = after 1 h 30' irradiation. Analyses were conducted under **HPLC-4 conditions**.

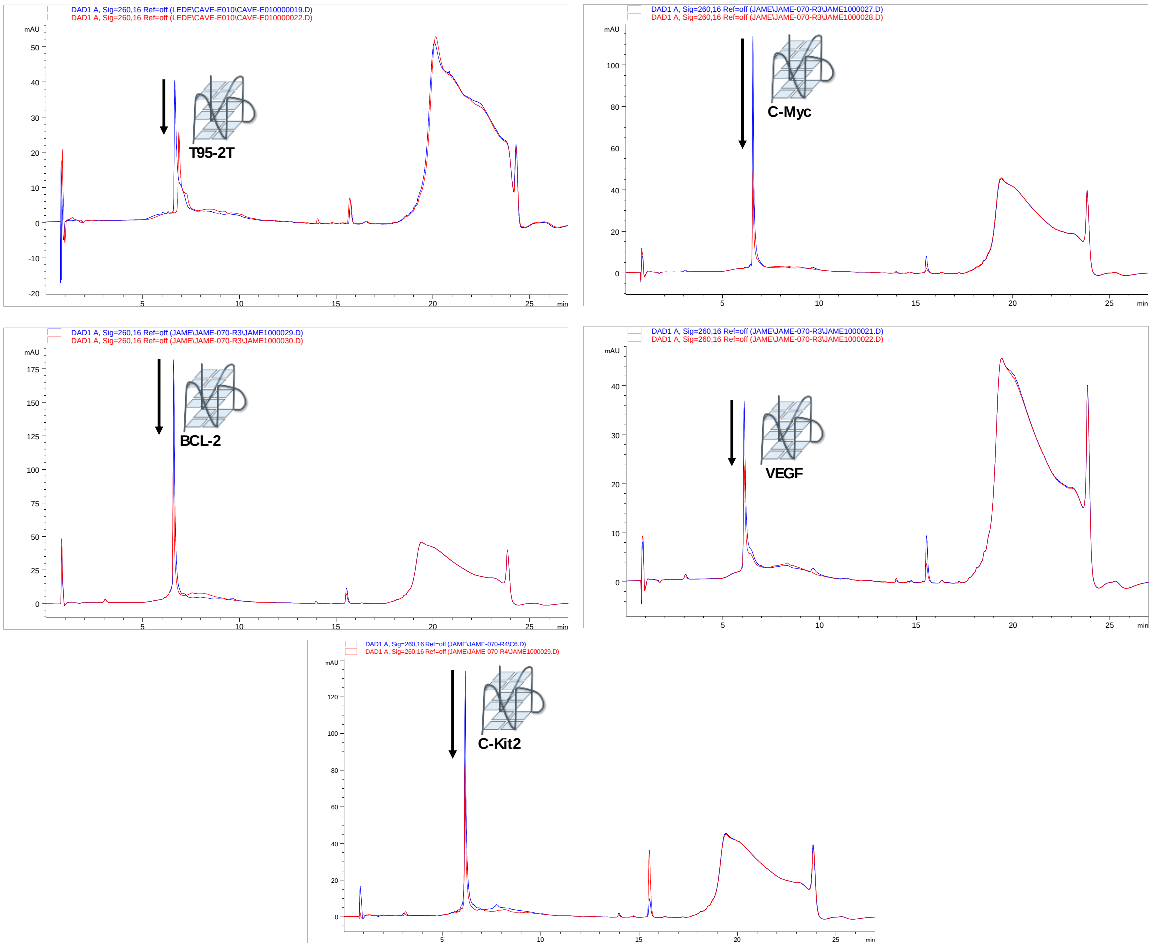


**Figure S10**. HPLC alkylation experiment of parallel G4 sequences (T95-2T, c-Myc, BCL-2, VEGF, c-Kit2) with Y14:FurA (**10**). Blue trace = before irradiation, red trace = after 1 h 30’ irradiation. Analyses were conducted under **HPLC-4 conditions**.

## Alkylation experiments with BPA-peptides:

In a typical experiment, a 50 µL solution of 5 µM DNA was prepared in a 20 mM phosphate ($K_2HPO_4$) pH 7 buffer and pre-annealed by heating for 5 min at 95°C, followed by a cooling down period for 4h. 2.5 equivalents of BPA-peptide were added to the solution, equilibrated for 1 h and subjected to the 10 minutes of UVA light. Prior irradiation, a blank was taken. Finally, the samples were analysed using **HPLC-4 conditions**.

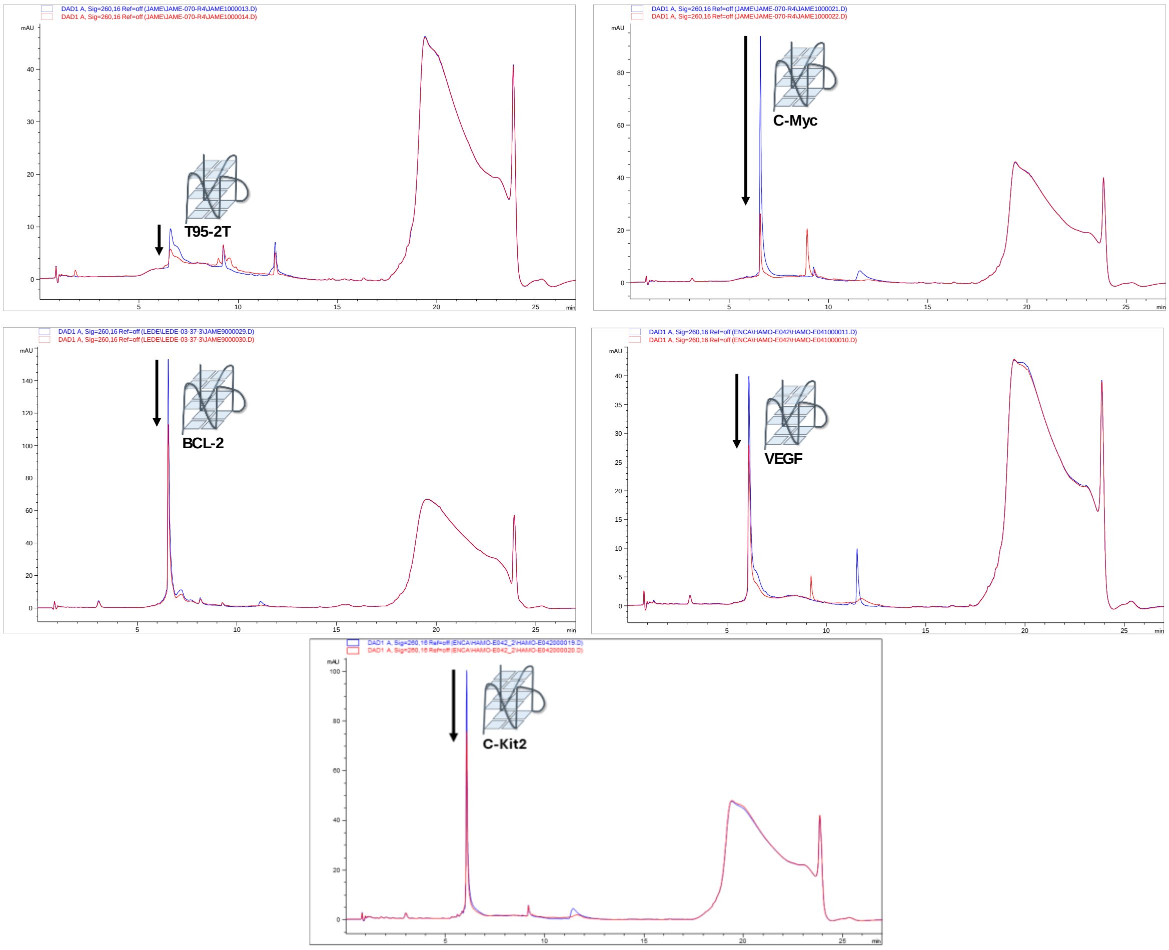


**Figure S11**. HPLC alkylation experiment of parallel G4 sequences (T95-2T, c-Myc, BCL-2, VEGF, c-Kit2) with Nt+BPA (**2**). Blue trace = before irradiation, red trace = after 10' irradiation. Analyses were conducted under **HPLC-4 conditions**.

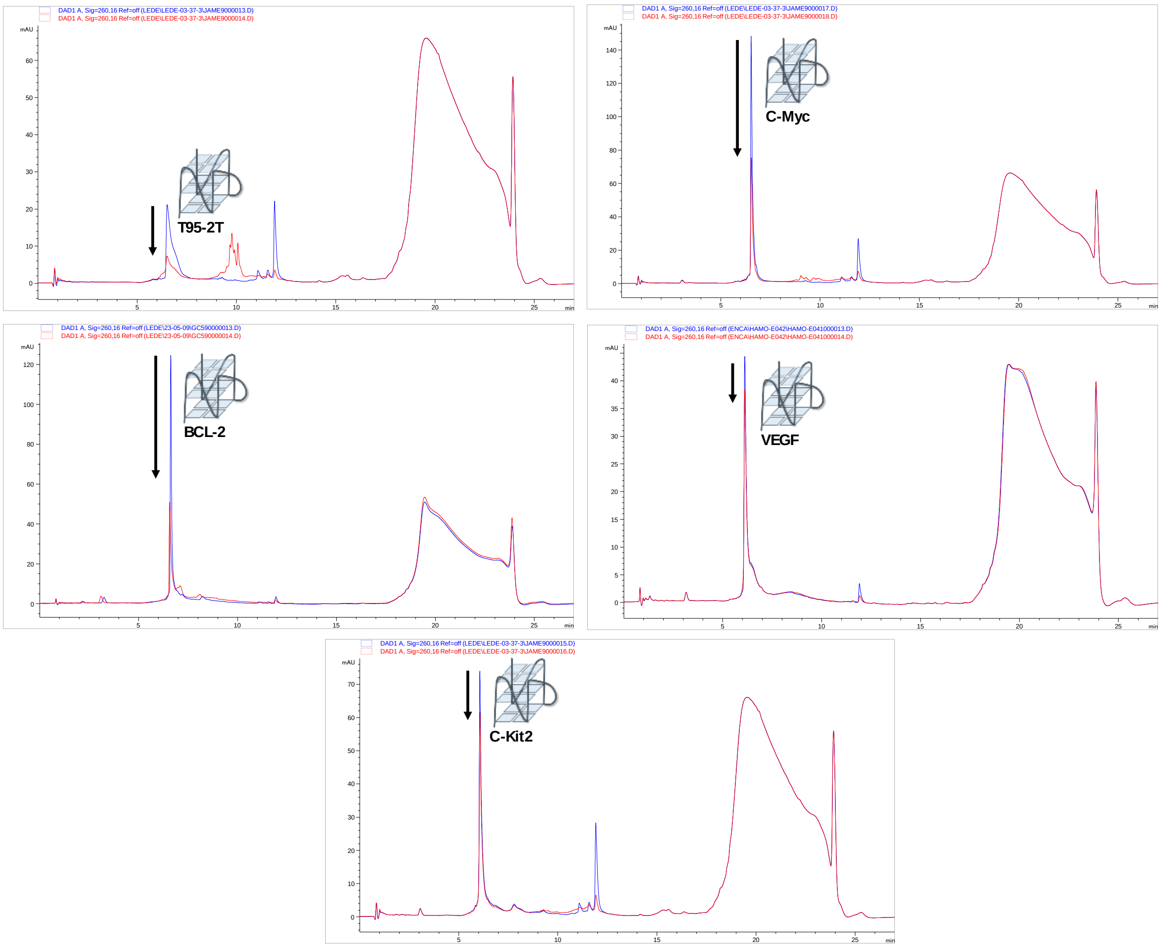


**Figure S12**. HPLC alkylation experiment of parallel G4 sequences (T95-2T, c-Myc, BCL-2, VEGF, c-Kit2) with K6:BPA (**3**). Blue trace = before irradiation, red trace = after 10’ irradiation. Analyses were conducted under **HPLC-4 conditions**.

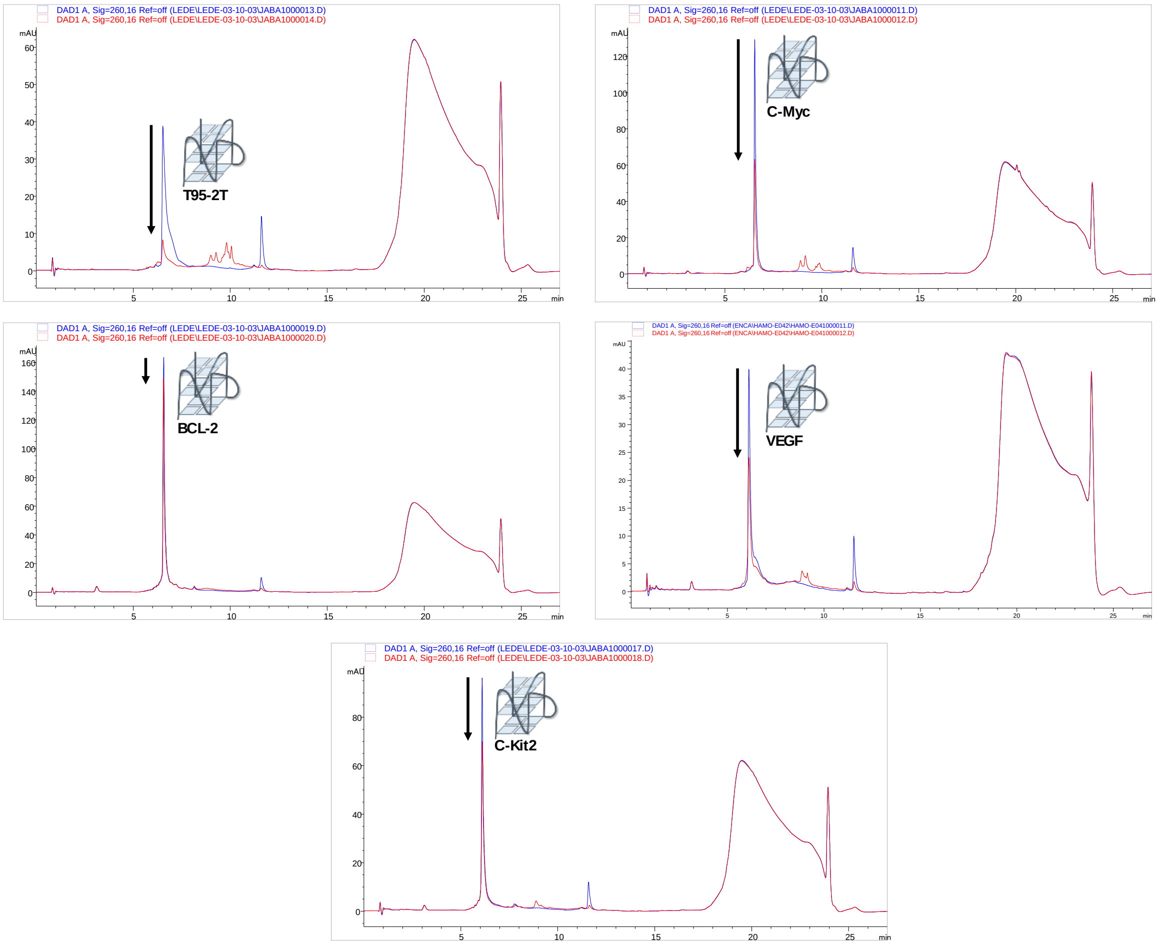


**Figure S13**. HPLC alkylation experiment of parallel G4 sequences (T95-2T, c-Myc, BCL-2, VEGF, c-Kit2) with K6+BPA (**4**). Blue trace = before irradiation, red trace = after 10' irradiation. Analyses were conducted under **HPLC-4 conditions**.

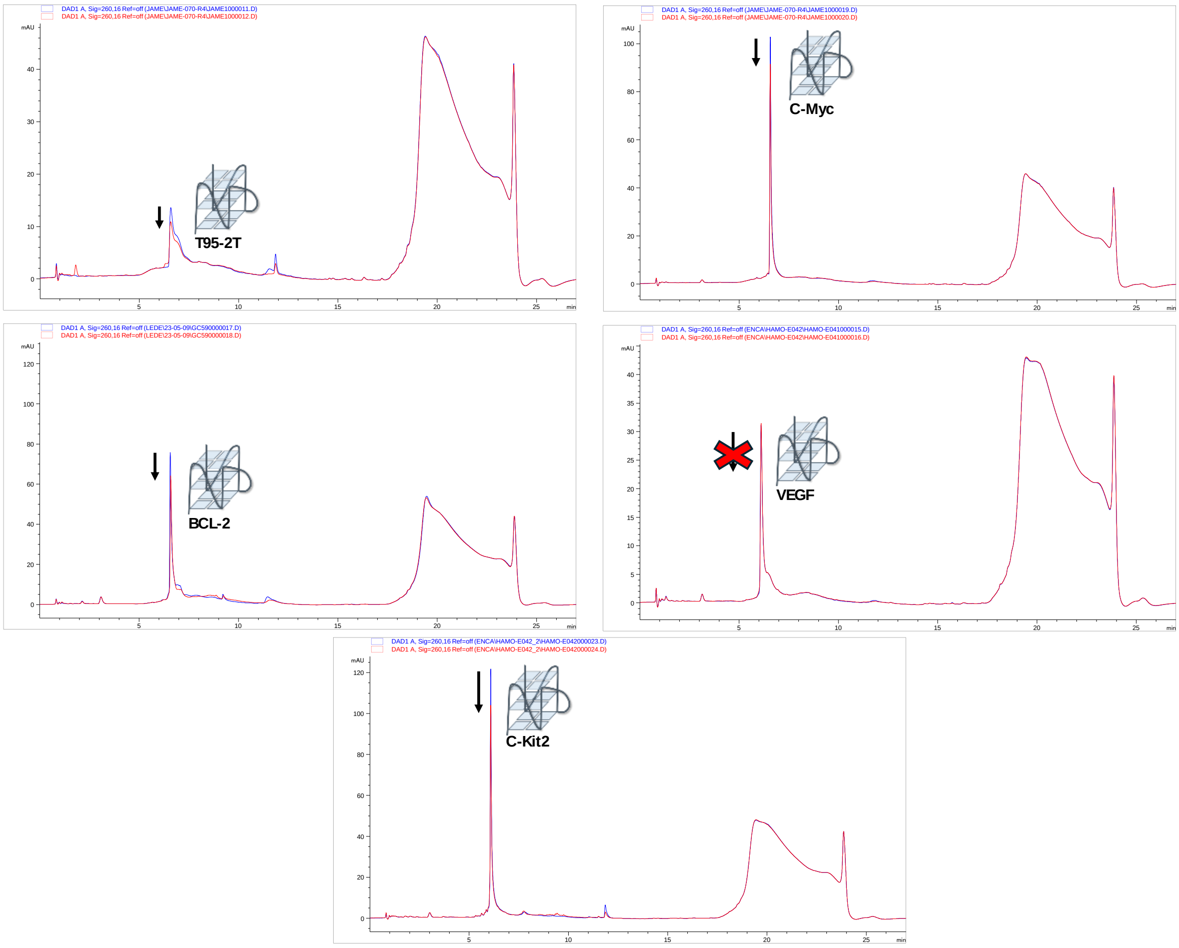


**Figure S14**. HPLC alkylation experiment of parallel G4 sequences (T95-2T, c-Myc, BCL-2, VEGF, c-Kit2) with Y14:BPA (**5**). Blue trace = before irradiation, red trace = after 10' irradiation. Analyses were conducted under **HPLC-4 conditions**.

## Mass experiments:

The formed alkylation products were purified by HPLC, desalted, concentrated and analysed by MALDI-TOF and MS-orbitrap analysis.

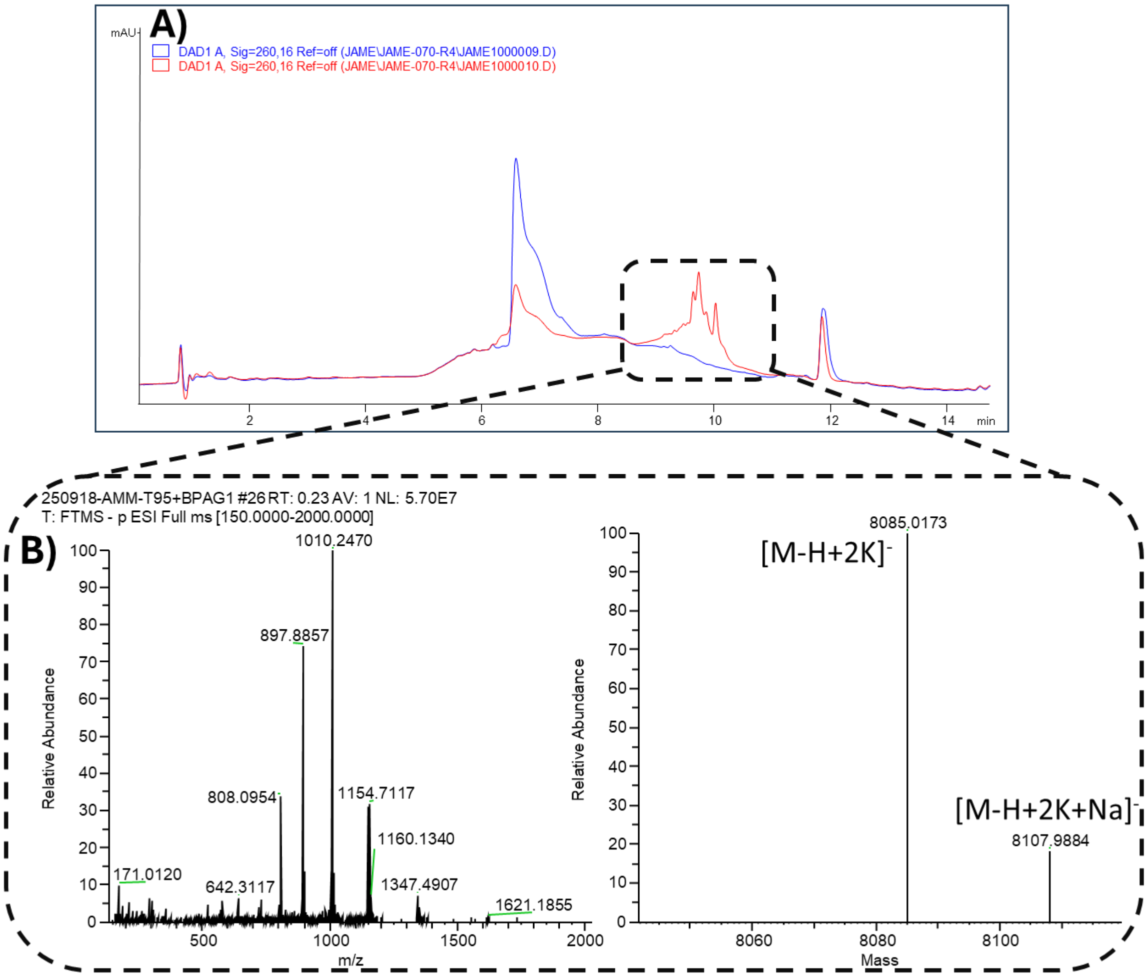


**Figure S15**. Mass analysis of the formed alkylation products. **A)** HPLC alkylation experiment of the parallel T95-2T with K6:BPA (**3**). **B)** Orbitrap-MS results confirming the formation of a covalent peptide-G4 adduct. Calculated mass (sum of DNA and peptide mass): m/z: 8010 Da.

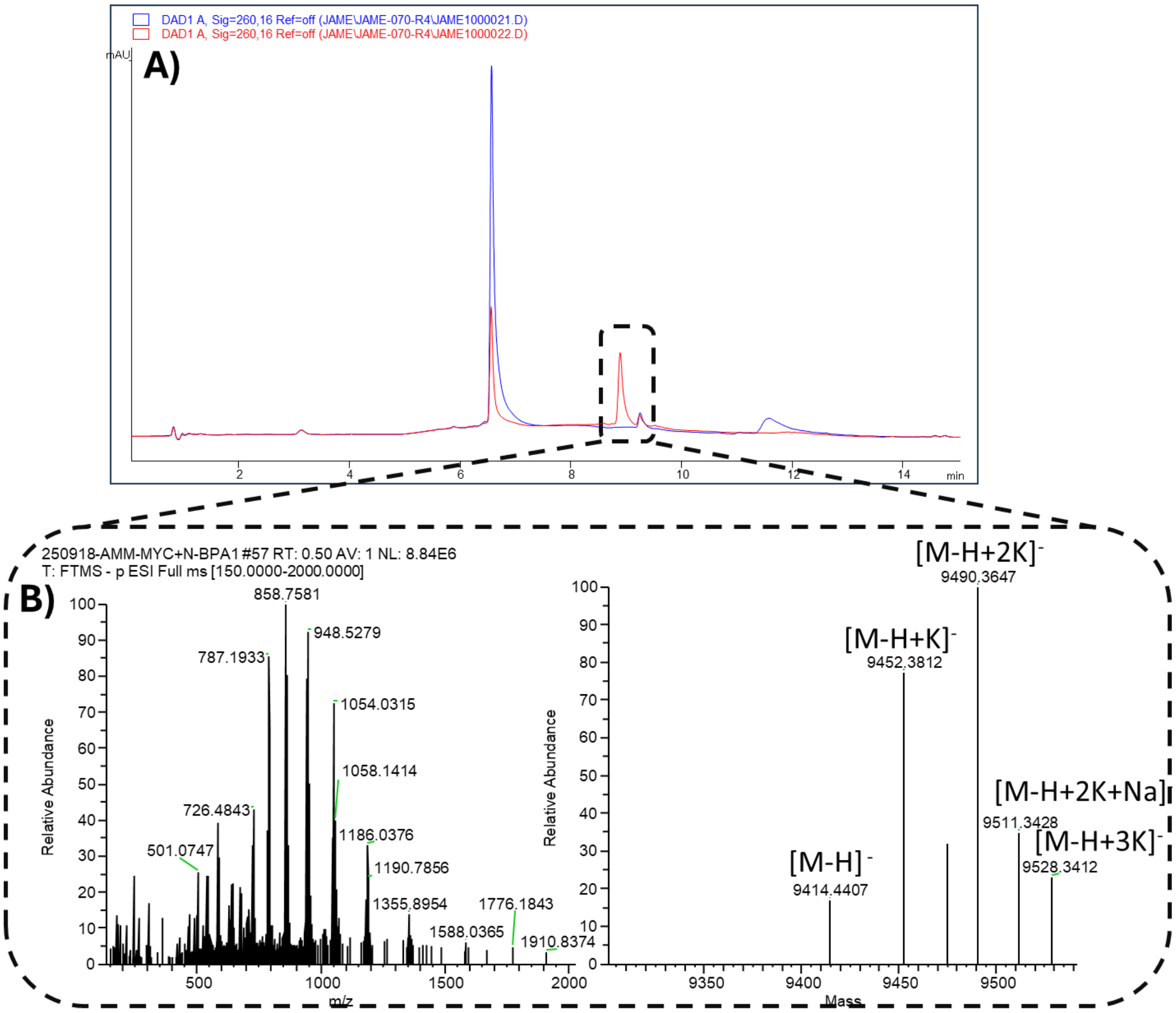


**Figure S16**. Mass analysis of the formed alkylation products. **A)** HPLC alkylation experiment of the parallel c-Myc with Nt+BPA (**2**). **B)** Orbitrap-MS results confirming the formation of a covalent peptide-G4 adduct. Calculated mass (sum of DNA and peptide mass): m/z: 9415 Da.

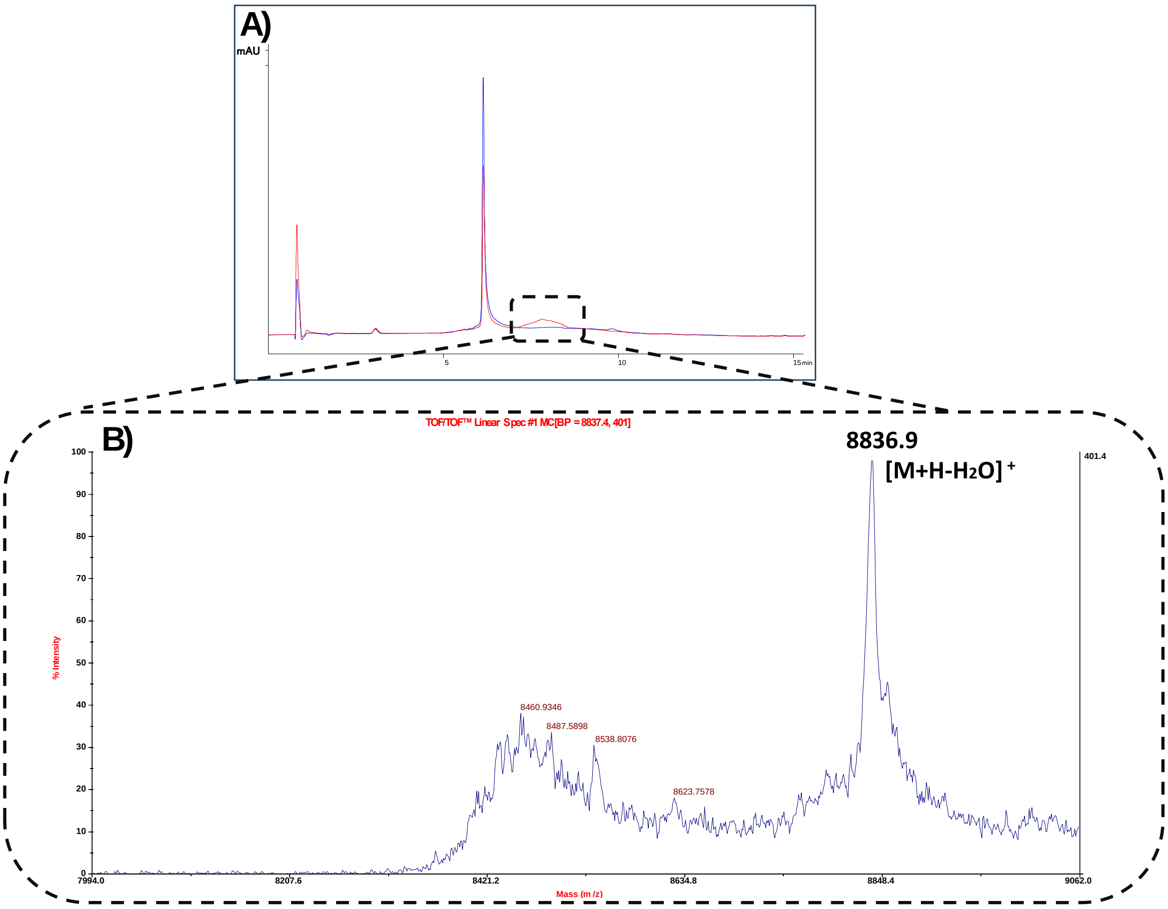


**Figure S17**. Mass analysis of the formed alkylation products. **A)** HPLC alkylation experiment of the parallel c-Kit2 with Nt+BPA (**2**). **B)** Orbitrap-MS results confirming the formation of a covalent peptide-G4 adduct. Calculated mass (sum of DNA and peptide mass): m/z: 8852 Da.

## PAGE experiments:

50 µL of a buffered solution (20 mM phosphate, $K_2HPO_4$, pH 7 buffer) containing 5 µM DNA was pre-annealed by heating for 5 min at 95°C, followed by a cooling down period for 4h. 2.5 eq of peptide was then added to the sample and left to equilibrate for 1 h. The furan-containing peptides were irradiated for 1 h 30' in presence of 20 mM Rhodamine B, whereas the benzophenone-containing peptides were irradiated with UV-A light for 10 minutes. 20 µL of the samples were then diluted with the appropriate loading dye (2X Tris-Glycine SDS running buffer for peptide conditions and 2X DNA loading dye, both purchased from Thermo Scientific nv).

*Peptide conditions:*

The samples were run on a 20% polyacrylamide gel (acrylamide:bisacrylamide 19:1) prepared in 250 mM Tris HCl buffer pH 8.8 containing 7 M urea and 10% SDS. 20 µL of a 1:1 sample/loading dye solution are loaded in each lane. The gel was run at constant voltage of 120 V. The power supply used for running the gel was provided by a consort EV202. Gels were stained with Silver staining (Thermo Fisher Scientific, Life Technologies, Merelbeke, Belgium) and imagined with a HP Photosmart scanner.

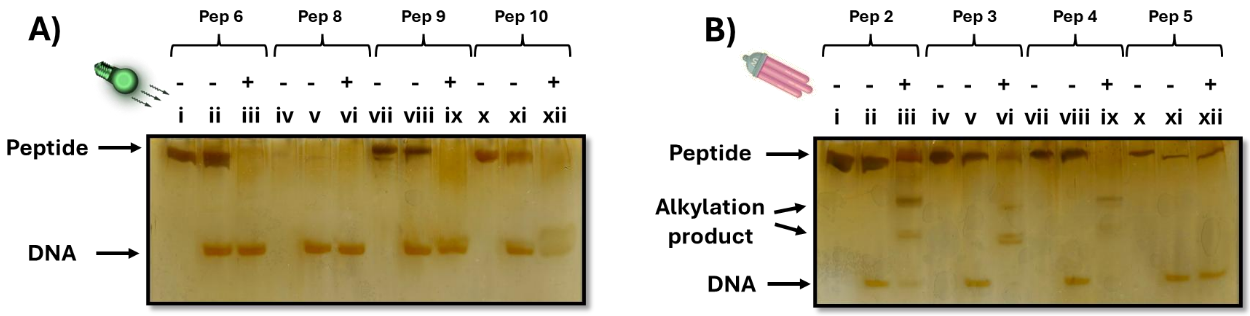


**Figure S18**. USDS-PAGE experiment of the alkylation reaction of **A**) c-Myc with the furan-containing peptides (Nt+FurA (**6**), K6:FurA (**8**), K6+FurA (**9**) and Y14:FurA (**10**) and **B**) T95-2T with the benzophenone-containing peptides (Nt+BPA (**2**), K6:BPA6 (**3**), K6+BPA (**4**) and Y14:BPA (**5**). Left lanes: i) **Peptide 6** alone; ii-iii) **Peptide 6** + c-Myc G4 before (ii) and after (iii) green light (550 nm) irradiation; iv) **Peptide 8** alone; v-vi) **Peptide 8** + c-Myc G4 before (v) and after (vi) green light (550 nm) irradiation; vii) **Peptide 9** alone; viii-ix) **Peptide 9** + c-Myc G4 before (viii) and after (ix) irradiation; x) **Peptide 10** alone; xi-xii) **Peptide 10** + c-Myc G4 before (xi) and after (xii) green light (550 nm) irradiation. Right lanes: i) **Peptide 2** alone; ii-iii) **Peptide 2** + T95-2T G4 before (ii) and after (iii) UV light (365 nm) irradiation; iv) **Peptide 3** alone, v-vi) **Peptide 3** + T95-2T G4 before (v) and after (vi) UV light (365 nm) irradiation; vii) **Peptide 4** alone, viii-ix) **Peptide 4** + T95-2T G4 before (viii) and after (ix) UV light (365 nm) irradiation; x) **Peptide 5** alone, xi-xii) **Peptide 5** + T95-2T G4 before (xi) and after (xii) irradiation. The gels were run on a 20% polyacrylamide gel at a constant voltage of 120 V and stained with silver staining. The differences in peptide band intensity depend on their potential interactions with the silver stain (and thus do not reflect differences in concentration).

**Table S3**: Densitometric analysis of the DNA band, derived from the USDS-PAGE experiment under peptide conditions. Note: No internal standard was used, thus the obtained %DNA band disappearance represents rather a qualitative than a quantitative control.

| Sample | % DNA band disappearance |
|---|---|
| c-Myc + Nt+FurA (**6**) | 13% |
| c-Myc + K6:FurA (**8**) | 22% |
| c-Myc + K6+FurA (**9**) | 12% |
| c-Myc + Y14:FurA (**10**) | 41% |
| T95-2T + Nt+BPA (**2**) | 49% |
| T95-2T + K6:BPA (**3**) | 91% |
| T95-2T + K6+BPA (**4**) | 92% |
| T95-2T + Y14:BPA (**5**) | 4% |

*DNA conditions:*

The samples were run on a 15% polyacrylamide gel (acrylamide:bisacrylamide 19:1) prepared in 1X Tris-borate buffer containing 7 M urea. 20 µL of a 1:1 sample/loading dye solution are loaded in each lane. The gel was run at constant voltage of 120 V. The power supply used for running the gel was provided by a consort EV202. Gels were stained with SYBR gold (Thermo Fisher Scientific, Life Technologies, Merelbeke, Belgium) and imaged using an Autochemi imaging system (UVP).

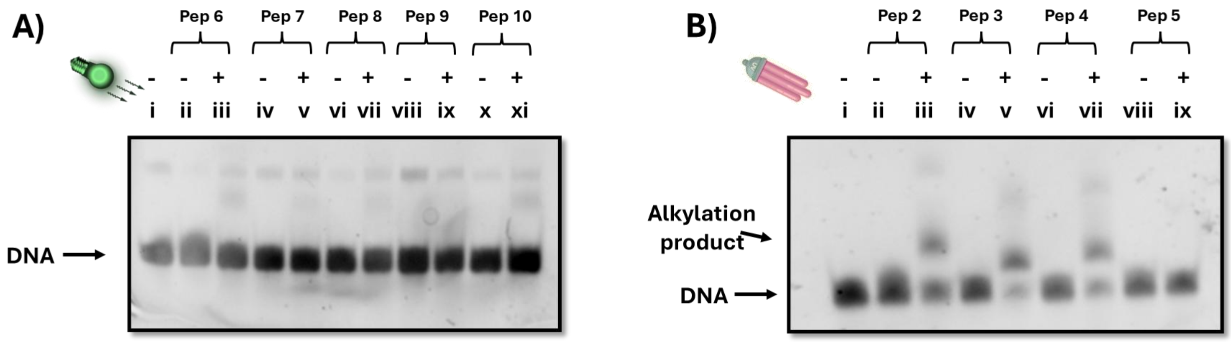


**Figure S19**. Denaturing PAGE experiment of the alkylation reaction of **A**) c-Myc with the furan-containing peptides (Nt+FurA (**6**), H1:FurA (**7**), K6:FurA (**8**), K6+FurA (**9**) and Y14:FurA (**10**) and **B**) T95-2T with the benzophenone-containing peptides (Nt+BPA (**2**), K6:BPA6 (**3**), K6+BPA (**4**) and Y14:BPA (**5**). Left lanes: i) c-Myc G4 alone; ii-iii) c-Myc G4 + **Peptide 6** before (ii) and after (iii) green light (550 nm) irradiation; iv-v) c-Myc G4 + **Peptide 7** before (iv) and after (v) green light (550 nm) irradiation**;** vi-vii) c-Myc G4 + **Peptide 8** before (vi) and after (vii) green light (550 nm) irradiation; viii-ix) c-Myc G4 + **Peptide 9** before (viii) and after (ix) green light (550 nm) irradiation; x-xi) c-Myc G4 + **Peptide 10** before (x) and after (xi) green light (550 nm) irradiation. Right lanes: i) T95-2T G4 alone, ii-iii) T95-2T G4 + **Peptide 2** before (ii) and after (iii) UV light (365 nm) irradiation; iv-v) T95-2T G4 + **Peptide 3** before (iv) and after (v) UV light (365 nm) irradiation; vi-vii) T95-2T G4 + **Peptide 4** before (vi) and after (vii) UV light (365 nm) irradiation; viii-ix) T95-2T G4 + **Peptide 5** before (viii) and after (ix) UV light (365 nm) irradiation. The gels were run on a 15% polyacrylamide gel at a constant voltage of 120 V and stained with SYBR gold staining.

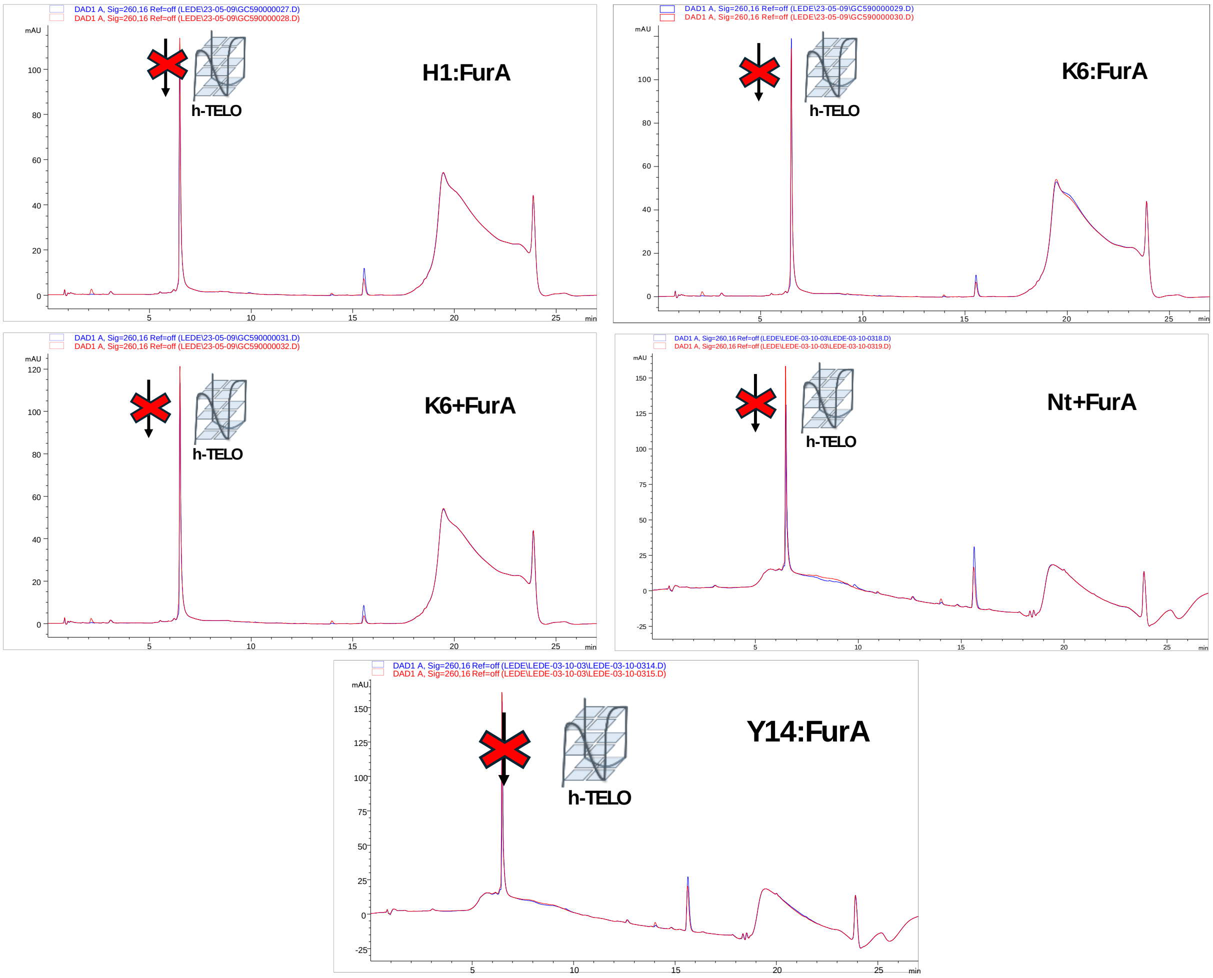


**Figure S20**. HPLC alkylation experiment of non-parallel h-TELO G4 with Nt+FurA (**6**), H1:FurA (**7**), K6:FurA (**8**), K6+FurA (**9**) and Y14:FurA (**10**). Blue trace = before irradiation, red trace = after 1 h 30’ irradiation. Analyses were conducted under **HPLC-4 conditions**.

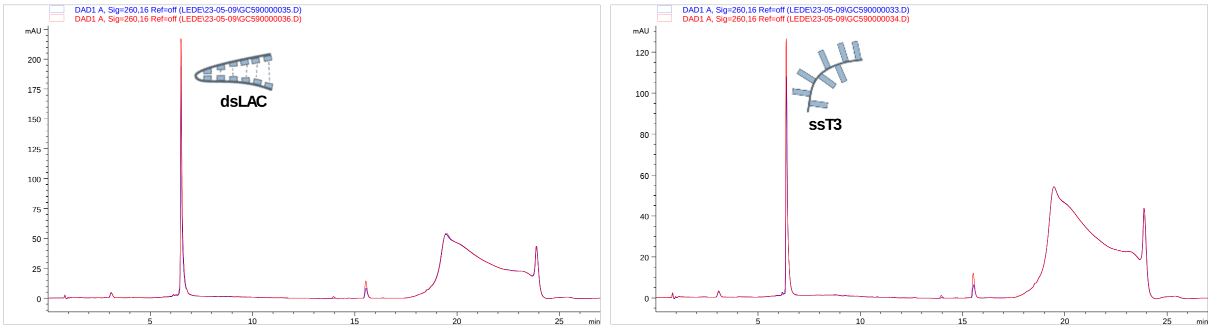


**Figure S21**. Control experiment: HPLC alkylation experiment of dsLAC (hairpin) and ssT3 (ssDNA) with K6:FurA (**8**). Blue trace = before irradiation, red trace = after 1 h 30’ irradiation. Analyses were conducted under **HPLC-4 conditions**.

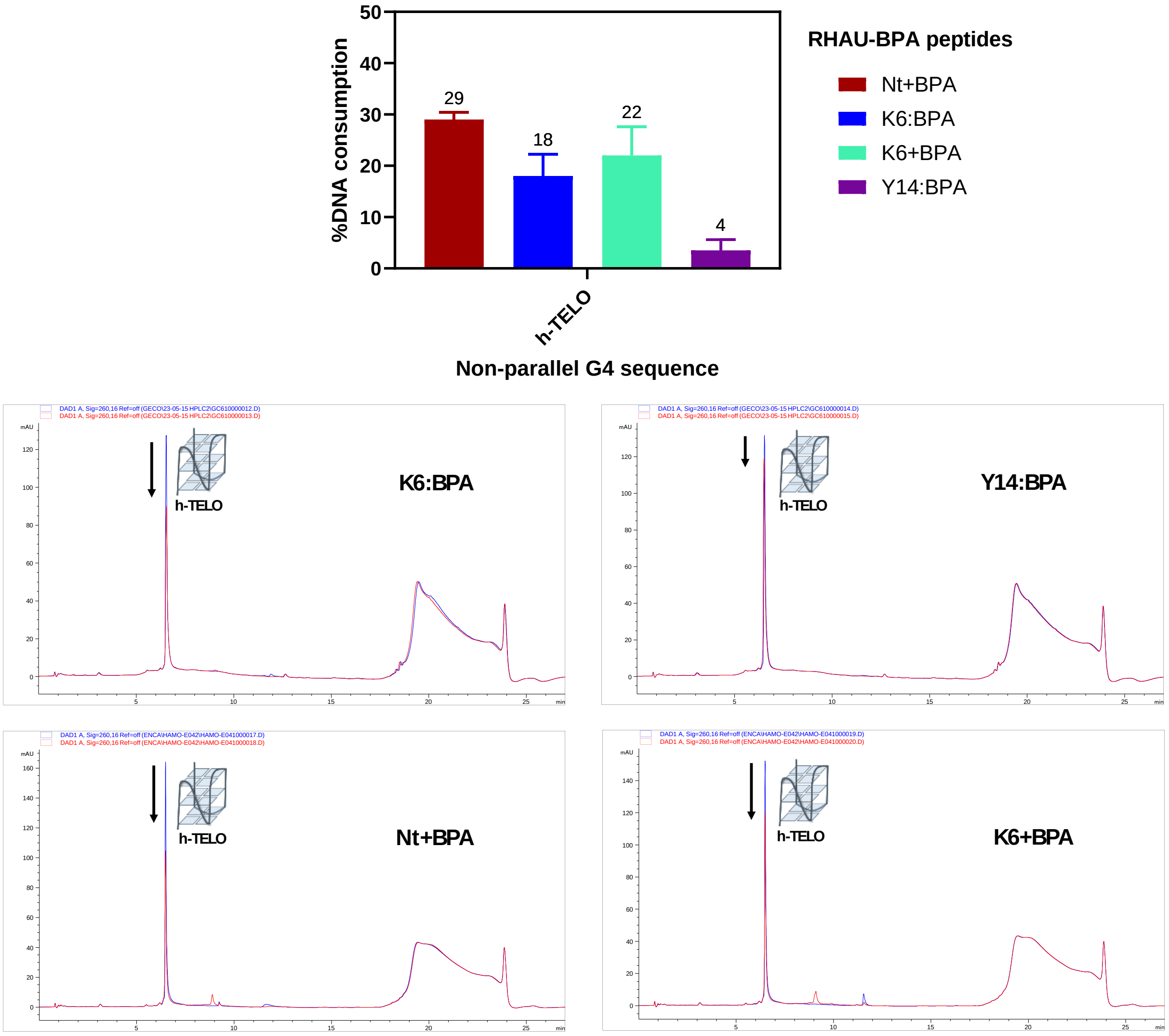


**Figure S22**. HPLC alkylation experiment of non-parallel h-TELO G4 with Nt+BPA (**2**), K6:BPA (**3**), Y14:BPA (**5**) and K6+BPA (**4**). Blue trace = before irradiation, red trace = after 10’ irradiation. Analyses were conducted under **HPLC-4 conditions**.

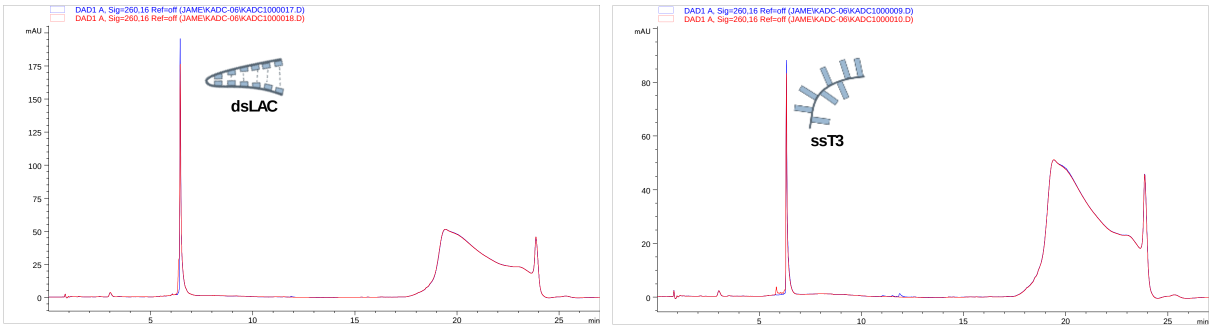


**Figure S23**. Control experiment: HPLC alkylation experiment of dsLAC (hairpin) and ssT3 (ssDNA) with K6:BPA (**3**). Blue trace = before irradiation, red trace = after 10’ irradiation. Analyses were conducted under **HPLC-4 conditions**.

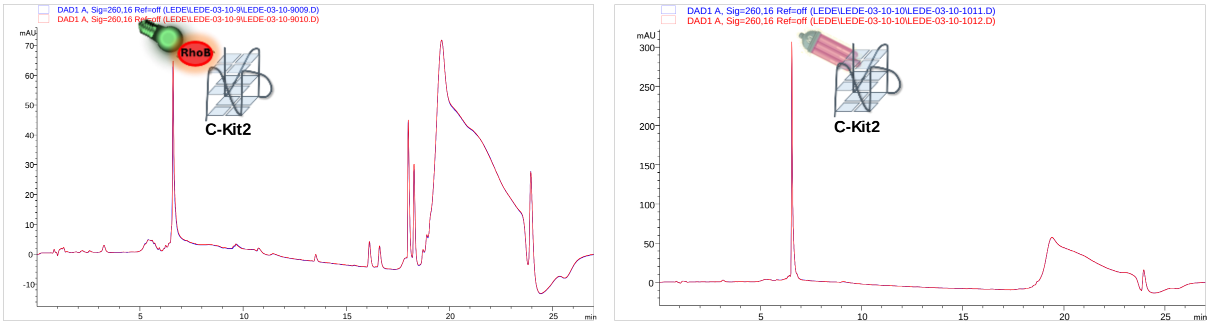


**Figure S24**. Control experiment: HPLC alkylation experiment of parallel c-Kit2 with RHAU18 (**1**) using both the FurA (left) and BPA activation conditions (right). Blue trace = before irradiation, red trace = after 10' irradiation. Analyses were conducted under **HPLC-4 conditions**.

## Competition experiments:

50 µL of a buffered solution (20 mM phosphate, $K_2HPO_4$, pH7) containing 5 µM DNA (both the FAM-labelled G4 and the competitor sequence) was pre-annealed by heating for 5 min at 95°C, followed by a cooling down period for 4h. 2.5 eq of peptide was then added to the sample and left to equilibrate for 1 h. The furan-containing peptides were irradiated for 1 h 30' in presence of 20 mM Rhodamine B, whereas the benzophenone-containing peptides were irradiated with UV-A light for 10 minutes. Before irradiation, each sample was aliquoted in a blank (no light exposure) and tested sample (light exposure). Alkylation efficiencies were determined by the %DNA consumption, calculated via integration of the DNA peak.

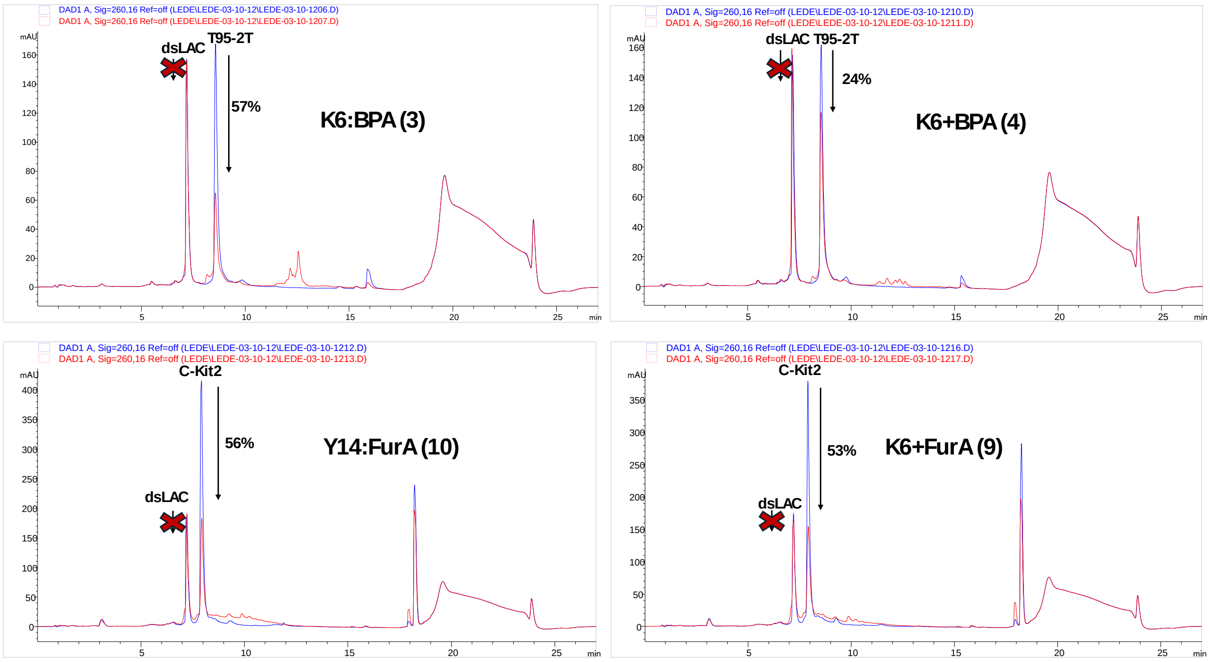


**Figure S25**. HPLC competition experiments of BPA-peptides (K6:BPA (**3**) and K6+BPA (**4**)) and FurA-peptides (K6+FurA (**9**) and Y14:FurA (**10**)) in presence of a FAM-labelled G4 sequence (T95-2T and c-Kit2) and non-G4 competitor (dsLAC). Analyses were conducted under **HPLC-4 conditions**.

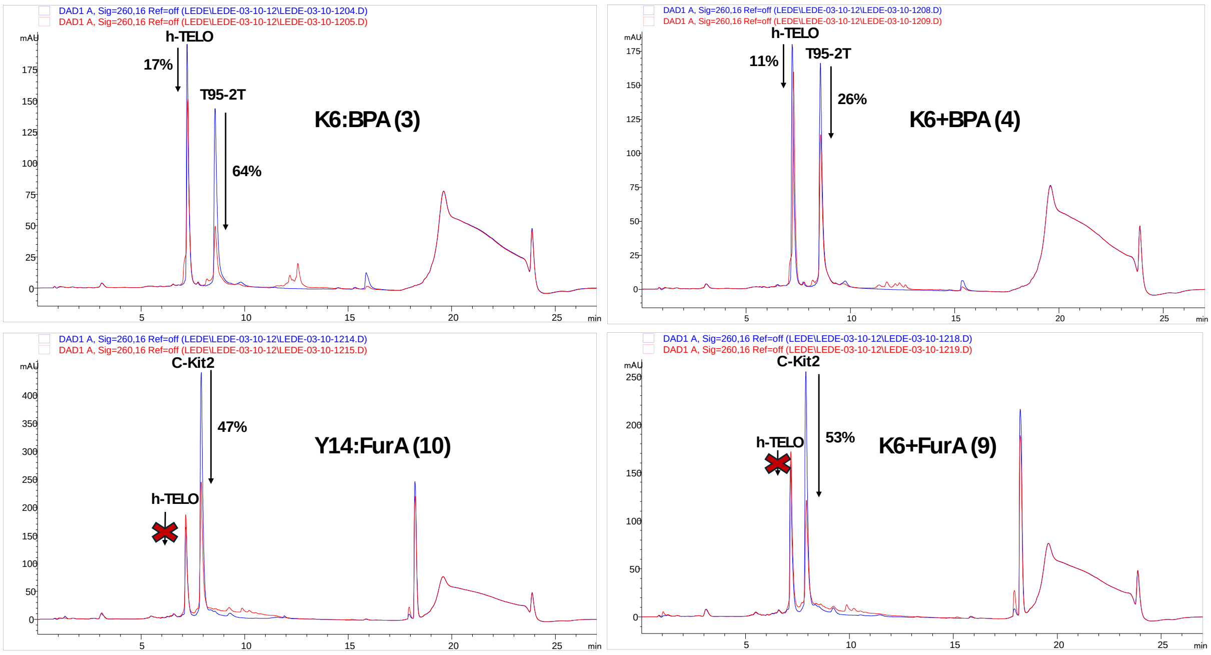


**Figure S26**. HPLC competition experiments of BPA-peptides (K6:BPA (**3**) and K6+BPA (**4**)) and FurA-peptides (K6+FurA (**9**) and Y14:FurA (**10**)) in presence of a parallel FAM-labelled G4 sequence (T95-2T and c-Kit2) and hybrid G4 competitor (h-TELO). Analyses were conducted under **HPLC-4 conditions**.

## 4. UV-melting experiments with first set of peptides

In a typical experiment, a 150 µL DNA solution (5 µM) was prepared in a 20 mM Tris HCl pH 7.4 buffer, containing either 10 mM KCl (for BCL-2, c-Kit2, VEGF, h-TELO and dsDNA) and 25 mM NaCl (for c-Myc and T95-2T). Given the high Tm value of c-Myc (76°C) and T95-2T (87°C) in 10 mM KCl, we performed the melting experiments in NaCl-rich buffer. Each experiment was performed without or in presence of 2.2 equivalents of peptide, and the solution was pre-annealed by the instrument.

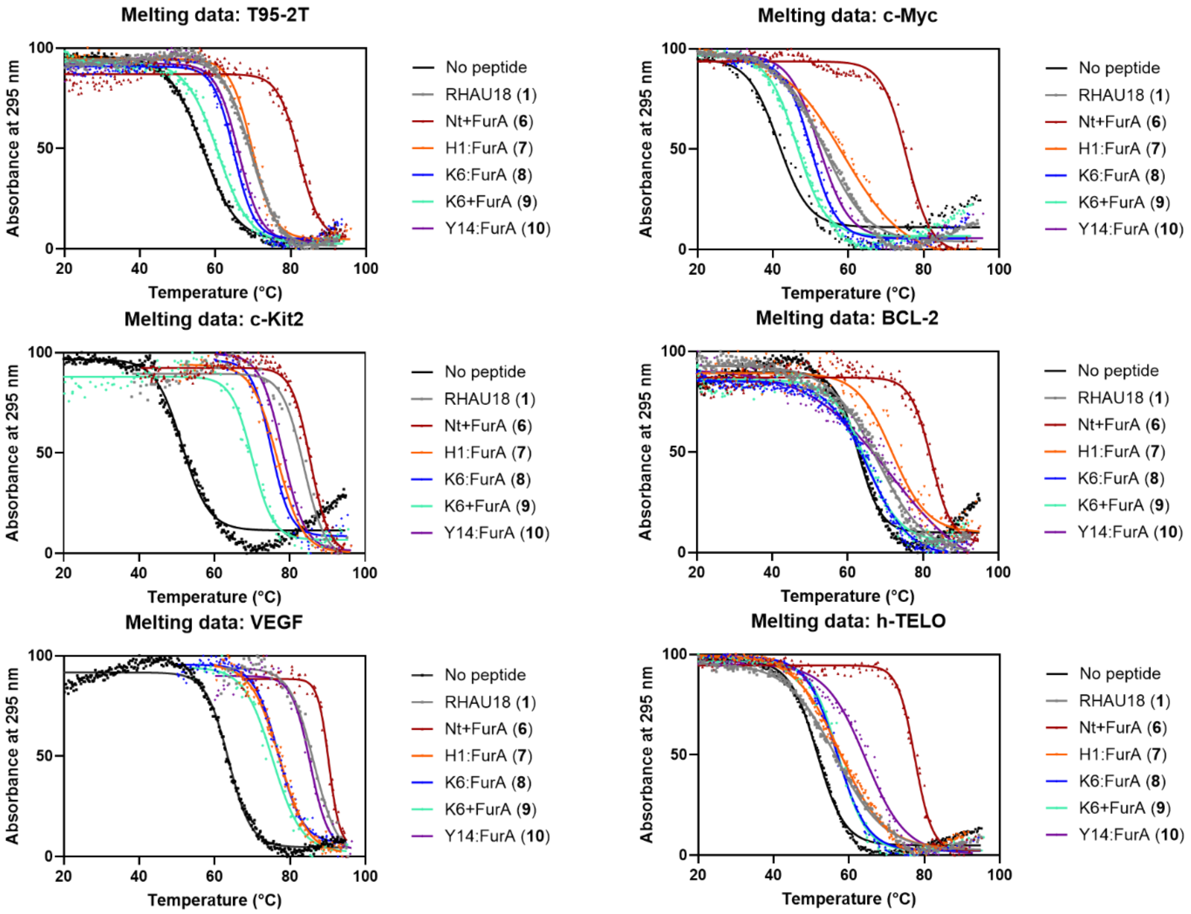


**Figure S27**. UV melting curves of all tested G4s with the FurA probes compared to RHAU18 and the G4 as such.

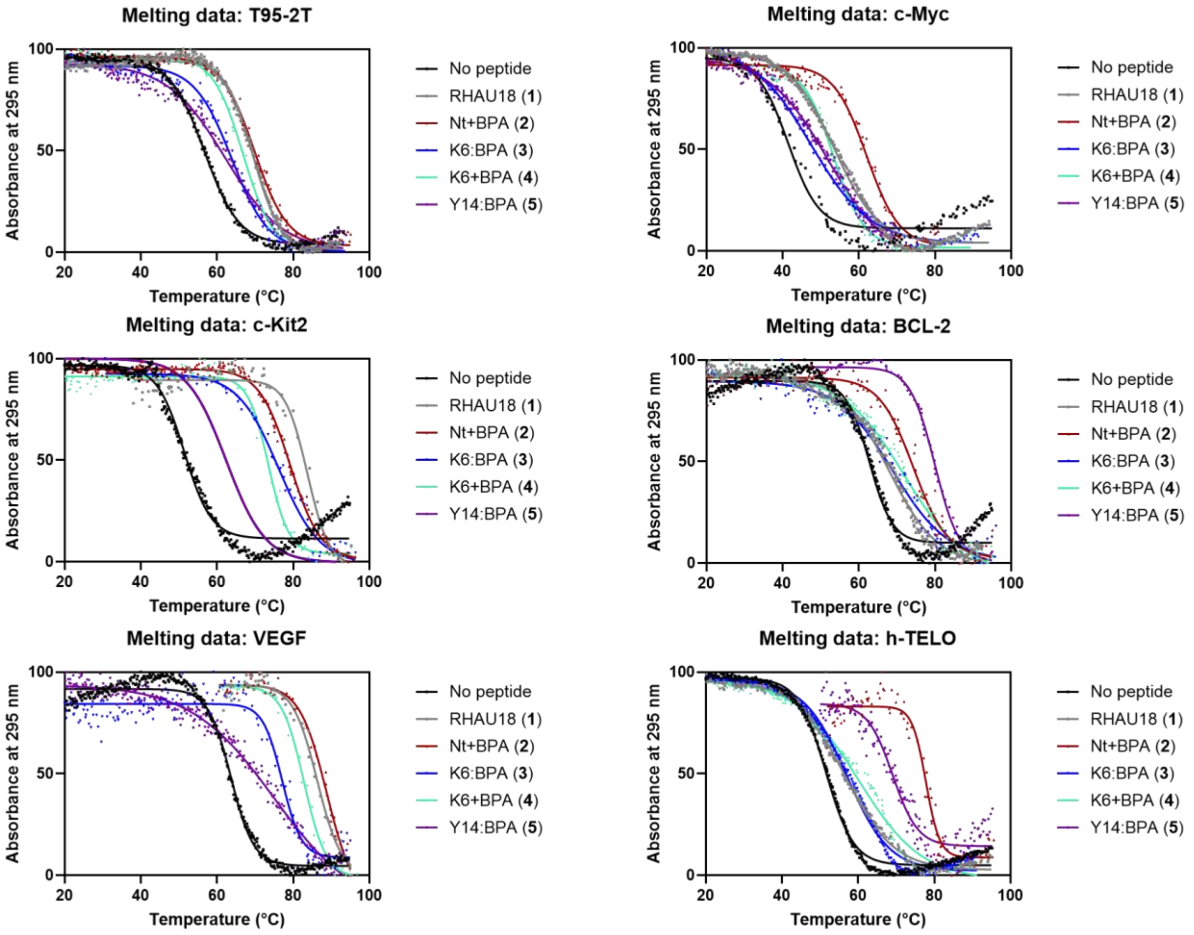


**Figure S28**. UV melting curves of all tested G4s with the BPA probes compared to RHAU18 and the G4 as such.

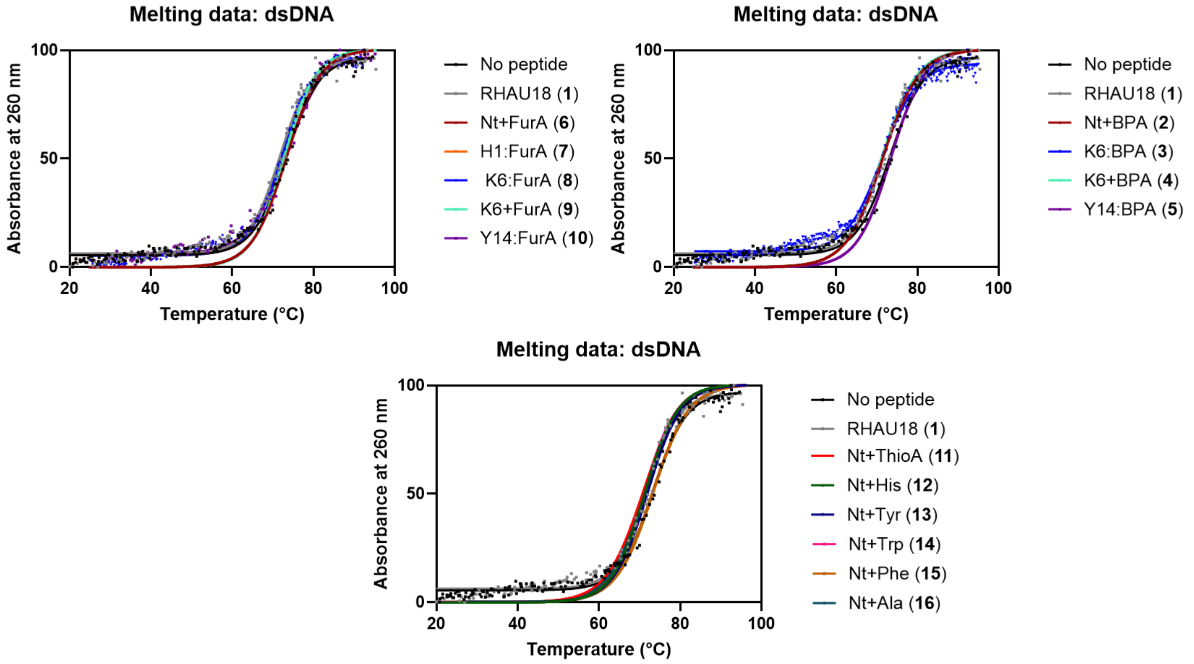


**Figure S29**. UV melting curves of dsDNA with all peptides (FurA probes at the top left; BPA probes at the top right and N-term modified probes at the bottom), compared to RHAU18 and dsDNA as such.

**Table S4**: Melting temperature values for the G4 sequences with and without the RHAU-FurA and -BPA peptides.

| G4 | $T_m$ (°C) | Used peptide | $\Delta T_m$ (°C) | G4 | $T_m$ (°C) | Used peptide | $\Delta T_m$ (°C) |
|---|---|---|---|---|---|---|---|
| T95-2T | 57.2 ± 0.2 | RHAU18 (**1**) | 12.2 ± 0.3 | BCL-2 | 62.9 ± 0.1 | RHAU18 (**1**) | 4.9 ± 1.6 |
| | | Nt+BPA (**2**) | 14.2 ± 1.3 | | | Nt+BPA (**2**) | 12.7 ± 1.7 |
| | | K6:BPA (**3**) | 6.7 ± 0.7 | | | K6:BPA (**3**) | 8.0 ± 0.4 |
| | | K6+BPA (**4**) | 9.4 ± 0.5 | | | K6+BPA (**4**) | 8.4 ± 1.0 |
| | | Y14:BPA (**5**) | 5.5 ± 0.6 | | | Y14:BPA (**5**) | 16.3 ± 0.9 |
| | | Nt+FurA (**6**) | 25.4 ± 0.5 | | | Nt+FurA (**6**) | 19.9 ± 1.3 |
| | | H1:FurA (**7**) | 12.9 ± 1.0 | | | H1:FurA (**7**) | 7.8 ± 1.2 |
| | | K6:FurA (**8**) | 7.2 ± 0.7 | | | K6:FurA (**8**) | 1.8 ± 0.8 |
| | | K6+FurA (**9**) | 3.5 ± 0.4 | | | K6+FurA (**9**) | 2.8 ± 1.4 |
| | | Y14:FurA (**10**) | 9.1 ± 0.4 | | | Y14:FurA (**10**) | 12.5 ± 0.2 |
| c-Myc | 41.0 ± 0.3 | RHAU18 (**1**) | 12.4 ± 0.4 | VEGF | 63.6 ± 0.1 | RHAU18 (**1**) | 23.2 ± 1.2 |
| | | Nt+BPA (**2**) | 20.9 ± 0.6 | | | Nt+BPA (**2**) | 24.4 ± 1.2 |
| | | K6:BPA (**3**) | 5.0 ± 1.7 | | | K6:BPA (**3**) | 13.3 ± 0.3 |
| | | K6+BPA (**4**) | 12.2 ± 0.7 | | | K6+BPA (**4**) | 18.4 ± 1.0 |
| | | Y14:BPA (**5**) | 10.4 ± 0.8 | | | Y14:BPA (**5**) | 22.9 ± 0.7 |
| | | Nt+FurA (**6**) | 34.3 ± 1.0 | | | Nt+FurA (**6**) | 27.4 ± 0.8 |
| | | H1:FurA (**7**) | 13.0 ± 1.0 | | | H1:FurA (**7**) | 14.3 ± 1.1 |
| | | K6:FurA (**8**) | 8.0 ± 1.8 | | | K6:FurA (**8**) | 17.2 ± 1.1 |
| | | K6+FurA (**9**) | 5.4 ± 0.4 | | | K6+FurA (**9**) | 11.3 ± 0.8 |
| | | Y14:FurA (**10**) | 10.3 ± 1.1 | | | Y14:FurA (**10**) | 20.6 ± 1.1 |
| c-Kit2 | 51.7 ± 0.3 | RHAU18 (**1**) | 31.5 ± 0.4 | h-TELO | 51.6 ± 0.1 | RHAU18 (**1**) | 5.3 ± 0.4 |
| | | Nt+BPA (**2**) | 27.4 ± 1.0 | | | Nt+BPA (**2**) | 25.3 ± 0.9 |
| | | K6:BPA (**3**) | 24.2 ± 1.0 | | | K6:BPA (**3**) | 4.8 ± 1.0 |
| | | K6+BPA (**4**) | 22.3 ± 1.5 | | | K6+BPA (**4**) | 8.2 ± 1.6 |
| | | Y14:BPA (**5**) | 10.4 ± 1.2 | | | Y14:BPA (**5**) | 19.2 ± 0.7 |
| | | Nt+FurA (**6**) | 34.3 ± 1.1 | | | Nt+FurA (**6**) | 26.1 ± 0.7 |
| | | H1:FurA (**7**) | 26.2 ± 0.9 | | | H1:FurA (**7**) | 6.7 ± 0.9 |
| | | K6:FurA (**8**) | 23.3 ± 0.8 | | | K6:FurA (**8**) | 3.9 ± 1.2 |
| | | K6+FurA (**9**) | 19.2 ± 1.5 | | | K6+FurA (**9**) | 3.8 ± 0.6 |
| | | Y14:FurA (**10**) | 27.2 ± 1.3 | | | Y14:FurA (**10**) | 13.6 ± 0.4 |

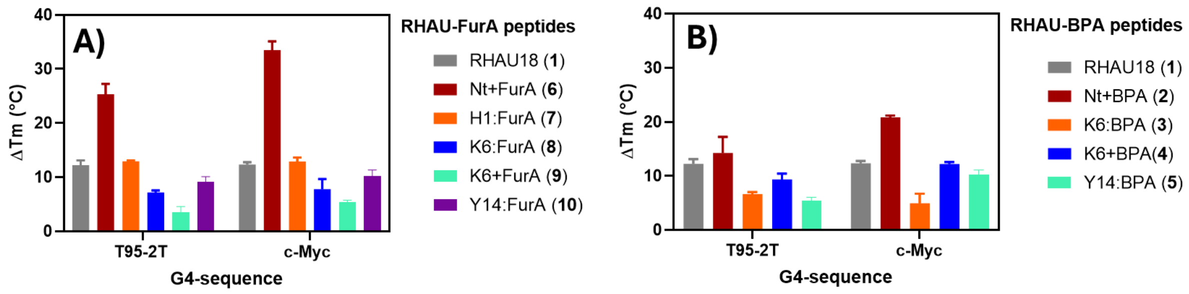


**Figure S30**. UV melting data of T95-2T and c-Myc G4 sequences in combination with **A**) RHAU-FurA peptide series and **B**) RHAU-BPA peptide series. Experiment was performed in a buffered solution (20 mM Tris HCl pH 7.4 buffer, supplemented with either 0.1 mM KCl or 25 mM NaCl at 5 µM strand concentration in a 1:2.2 DNA : peptide ratio.

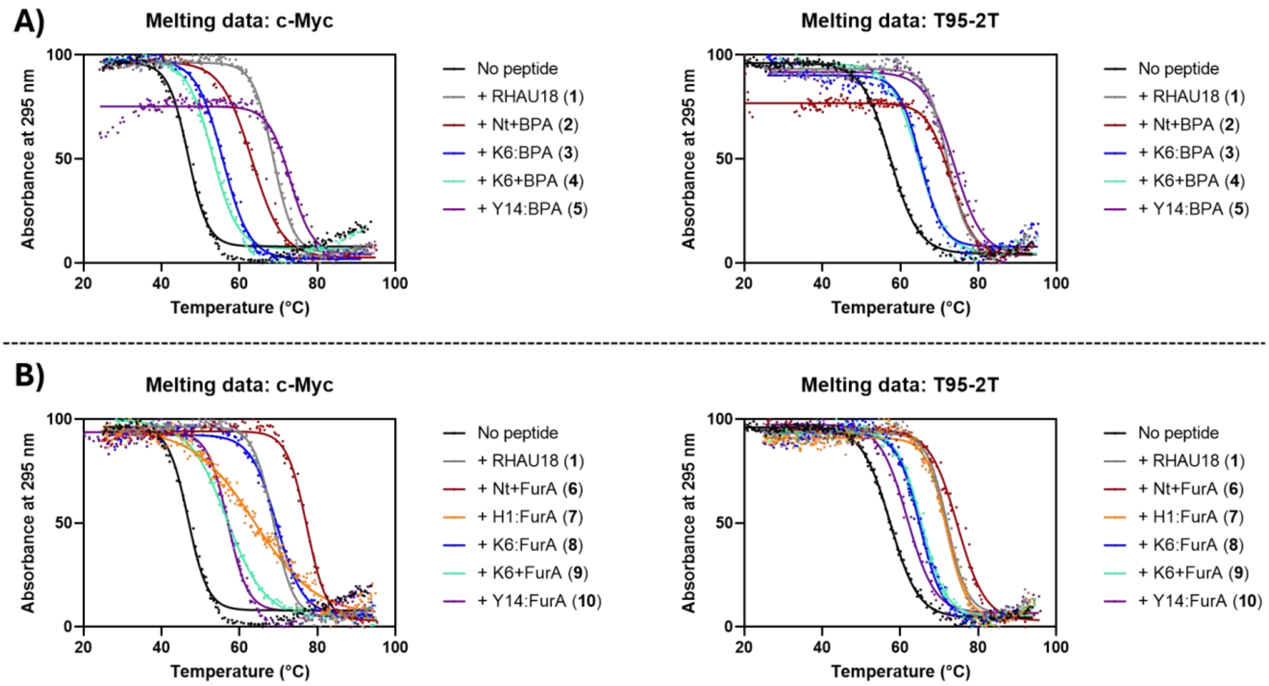


**Figure S31**. UV melting curves of c-Myc and T95-2T with the FurA (***A***) and BPA (***B***) probes compared to RHAU18 and the G4 as such. Experiment was performed in a buffered solution (20 mM Tris HCl pH 7.4 buffer, supplemented with 0.1 mM KCl.

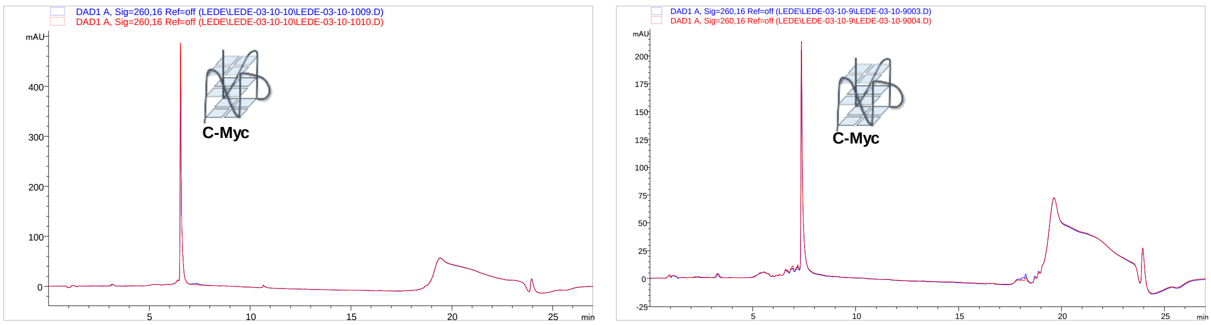


**Figure S32**. Control experiment: HPLC traces of c-Myc in presence of Nt+FurA (**6**, left) or Nt+BPA (**2**, right) before and after exposure to high-energy UV during UV melting experiments.

## 5. CD conformational shift experiments with FurA- and BPA-peptides

A 150 µL of 5 µM DNA solution was prepared in 20 mM Tris HCl pH 7.4 buffer containing 10 mM KCl (for hybrid h-TELO) or 25 mM NaCl (for anti-parallel h-TELO) or 25 mM KCl (for hTel21T18T and kit*). The solutions were pre-annealed either without or in presence of peptide by heating for 5 minutes at 95°C, followed by a cooling down period of 4 h. The CD signature was measured every day, for 2-4 days long, from 320 nm to 220 nm as wavelength range.

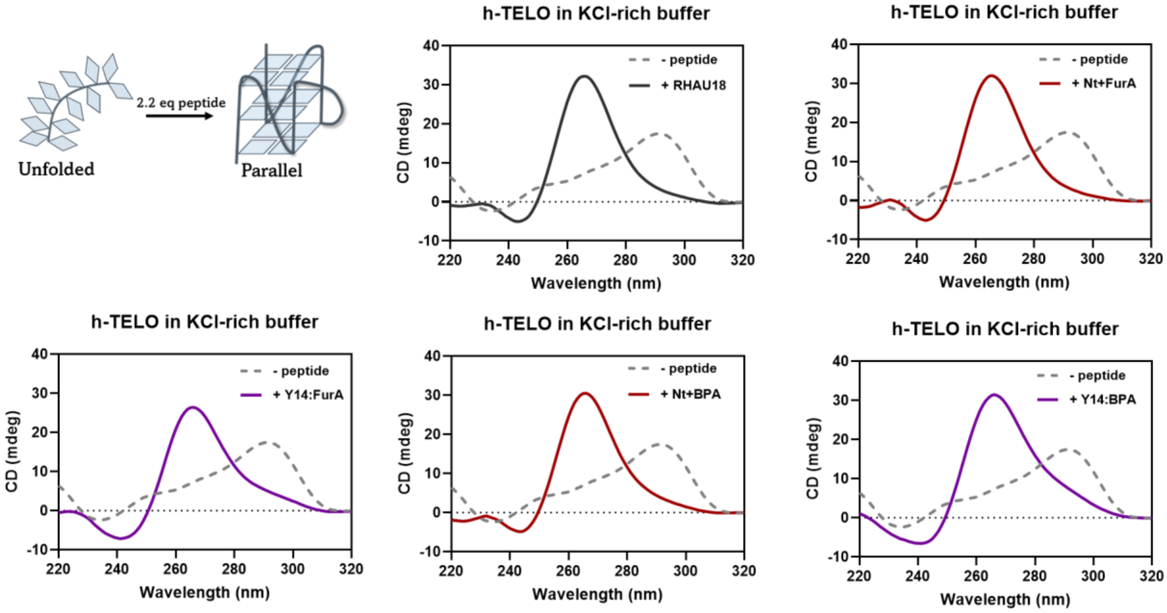


**Figure S33**. CD spectra of h-TELO in KCl-rich buffer, pre-annealed with RHAU18 (**1**), Nt+FurA (**6**), Y14:FurA (**10**), Nt+BPA (**2**) or Y14:BPA (**5**). The obtained spectrum was compared with the signature of the hybrid h-TELO as such (grey dotted line).

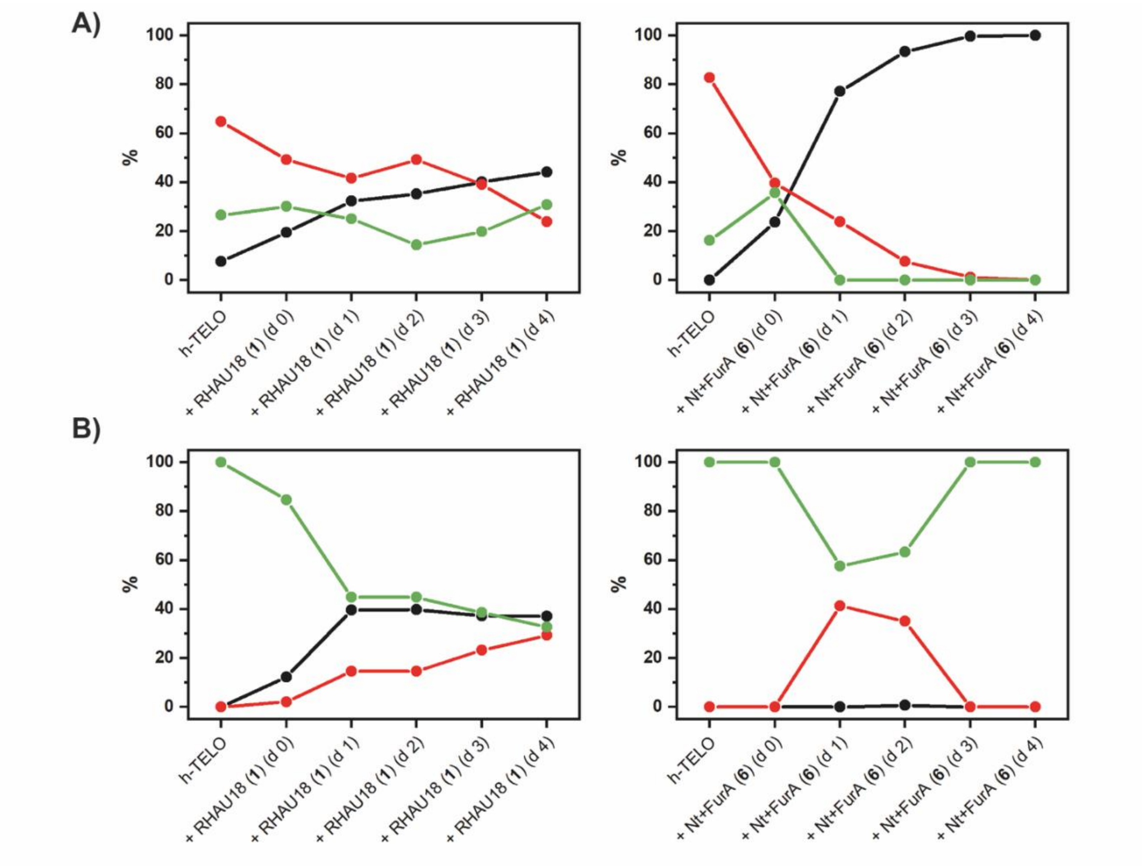


**Figure S34**. Time evolution of the estimated percentages of parallel (black), hybrid (red), and anti-parallel (green) G4 topologies for h-TELO in **A**) $K^+$- and **B**) $Na^+$-containing solution upon addition of (left panels) RHAU18 (**1**) and (right panels) Nt+FurA (**6**).

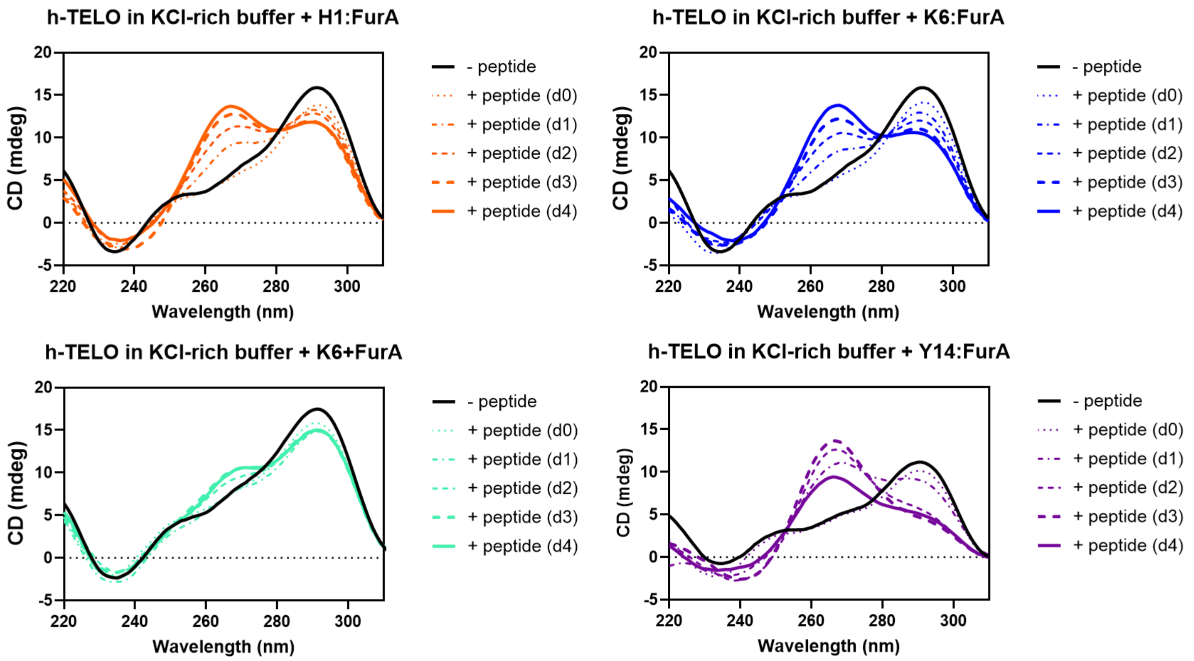


**Figure S35**. Kinetic refolding experiment: CD spectra of pre-annealed h-TELO in KCl-rich buffer, to which the RHAU-FurA derivatives were added (H1:FurA (**7**), K6:FurA (**8**), K6+FurA (**9**) and Y14:FurA (**10**)). The CD signature was measured over a time course of 4 days.

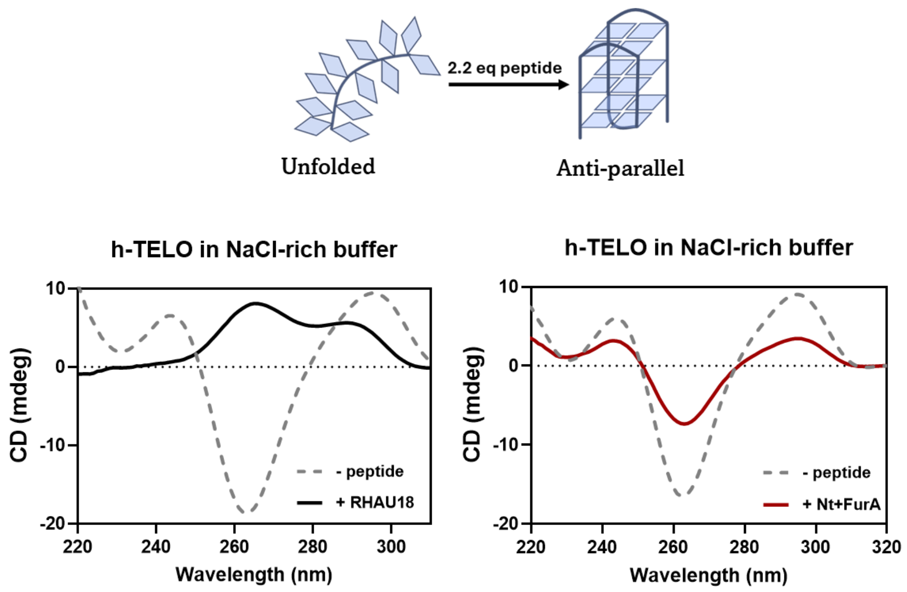


**Figure S36**. CD spectra of h-TELO in NaCl-rich buffer, pre-annealed with RHAU18 (**1**) and Nt+FurA (**6**). The obtained spectrum was compared with the signature of the anti-parallel h-TELO as such (grey dotted line).

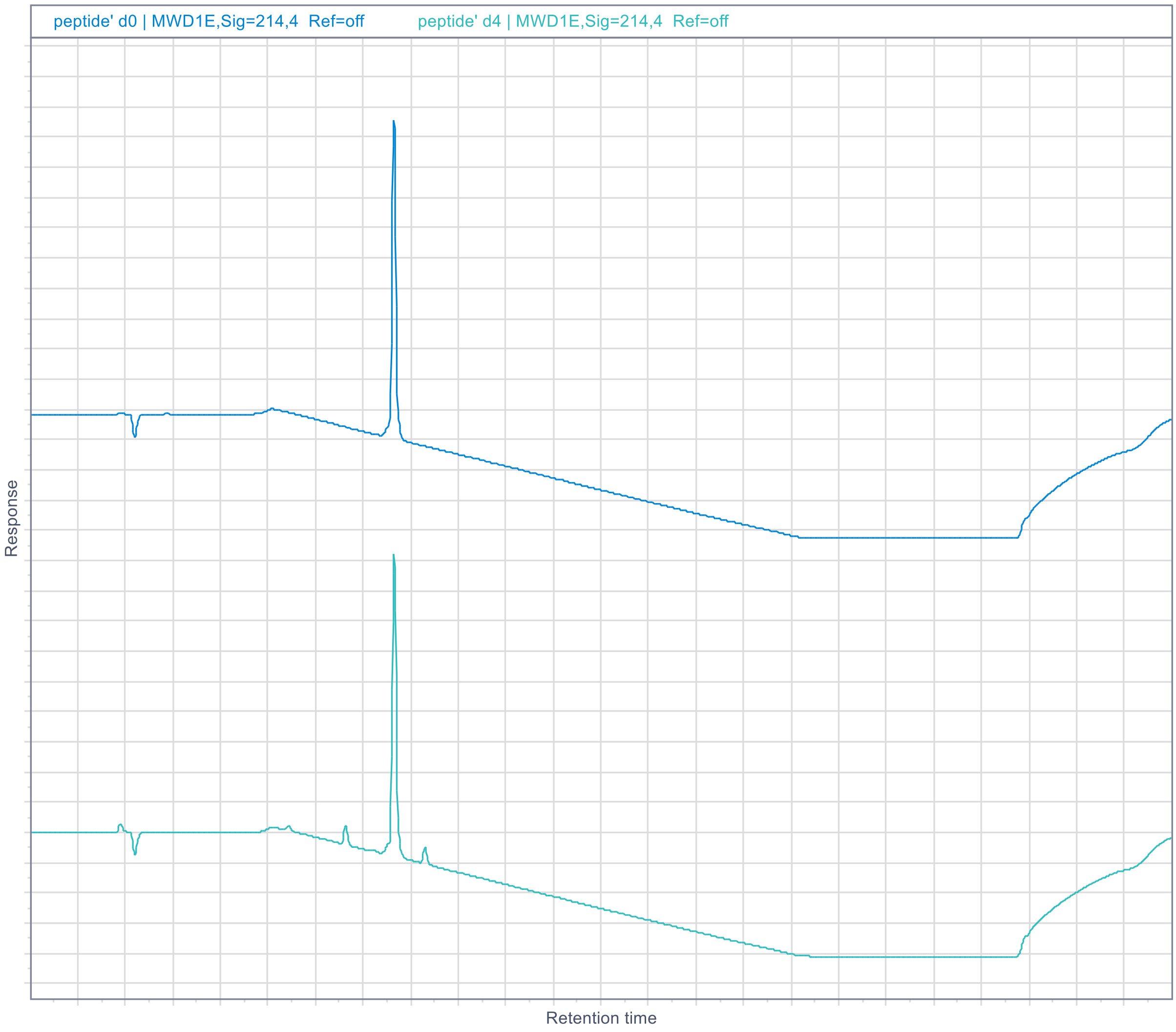


**Figure S37**. HPLC trace of Nt+FurA (**6**) on day 0 (dark blue, up) and day 4 (light blue, bottom).

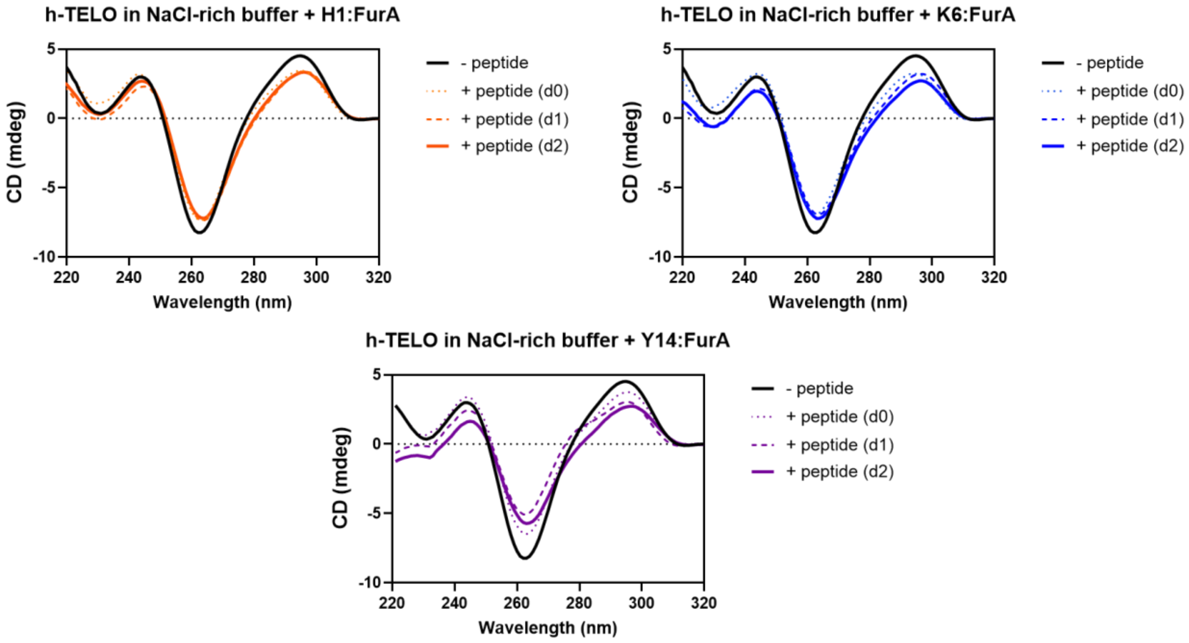


**Figure S38**. Kinetic refolding experiment: CD spectra of pre-annealed h-TELO in NaCl-rich buffer, to which the FurA derivatives were added (H1:FurA (**7**), K6:FurA (**8**) and Y14:FurA (**10**)). The CD signature was measured over a time course of 2 days.

## 7. CD characterisation of N-terminal modified peptides

250 µL of a 11 µM peptide solution was prepared in 20 mM Tris HCl pH 7.4 buffer containing 10 mM KCl. The CD signature was measured over a wavelength range from 260 nm to 190 nm (without T95-2T) or from 320 nm to 190 nm (in presence of T95-2T). For determining the CD signature of the peptide in complex with T95-2T, the CD signature of T95-2T as such was subtracted from the obtained value. The secondary structure content was estimated using the BestSel software.

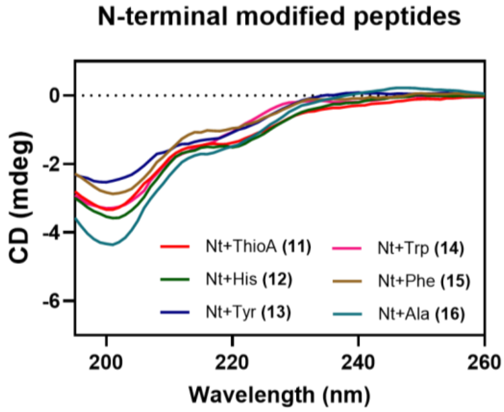


**Estimated secondary structure content (%) based on BeStSel software using CD data:**

| Peptide | Helix | Anti-parallel | Parallel | Turn | Others |
|---|---|---|---|---|---|
| Nt+ThioA (**11**) | 3.9 | 30.1 | 0.0 | 14.1 | 51.9 |
| Nt+His (**12**) | 4.9 | 29.6 | 0.0 | 19.5 | 49.9 |
| Nt+Tyr (**13**) | 3.1 | 36.3 | 0.0 | 14.8 | 45.9 |
| Nt+Trp (**14**) | 4.0 | 31.1 | 0.0 | 13.3 | 51.6 |
| Nt+Phe (**15**) | 3.8 | 32.4 | 0.0 | 14.7 | 49.1 |
| Nt+Ala (**16**) | 4.6 | 29.8 | 0.0 | 15.3 | 50.2 |

**Figure S39**. CD spectra and estimated secondary structure content of the N-terminal modified peptides.

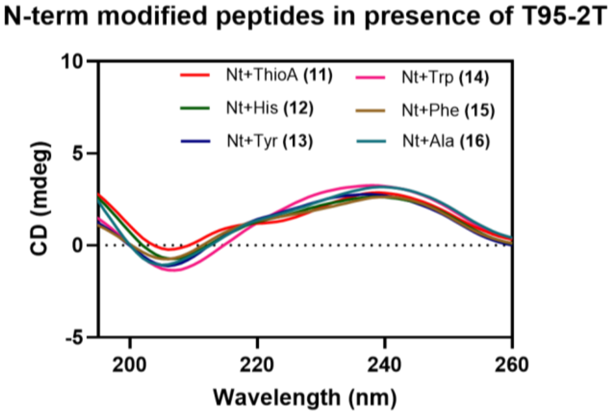


**Estimated secondary structure content (%) based on BestSel software using CD data:**

| Peptide | Helix | Anti-parallel | Parallel | Turn | Others |
|---|---|---|---|---|---|
| Nt+ThioA (**11**) | 13.8 | 49.9 | 0.0 | 0.0 | 36.2 |
| Nt+His (**12**) | 16.3 | 53.0 | 0.0 | 0.0 | 30.7 |
| Nt+Tyr (**13**) | 4.2 | 47.2 | 0.0 | 0.0 | 48.6 |
| Nt+Trp (**14**) | 13.8 | 49.9 | 0.0 | 0.0 | 32.6 |
| Nt+Phe (**15**) | 5.4 | 49.2 | 6.0 | 0.0 | 39.4 |
| Nt+Ala (**16**) | 11.5 | 24.5 | 0.0 | 15.2 | 48.8 |

**Figure S40**. CD spectra and estimated secondary structure content of the N-terminal modified peptides in presence of T95-2T (the CD signature of T95-2T was subtracted).

## 8. UV melting experiments with extended set of N-terminal modified peptides

In a typical experiment, a 150 µL DNA solution (5 µM) was prepared in a 20 mM Tris HCl pH 7.4 buffer, containing either 10 mM KCl (for BCL-2, c-Kit2, VEGF, h-TELO and dsDNA) and 25 mM NaCl (for c-Myc and T95-2T). Given the high Tm value of c-Myc (76 °C) and T95-2T (87 °C) in 10 mM KCl, we performed the melting experiments in NaCl-rich buffer. Each experiment was performed without or in presence of 2.2 equivalents of peptide, and the solution was pre-annealed by the instrument.

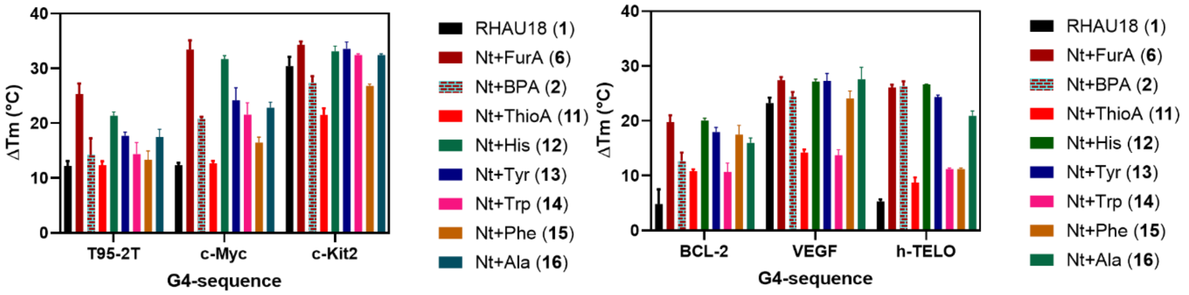


**Figure S41**. Induced thermal stability of all N-terminal modified peptides on parallel G4-sequences (T95-2T, c-Myc, c-Kit2, BCL-2 and VEGF) and non-parallel sequence (h-TELO) derived from UV melting experiments. The increase in Δ*Tm* appeared to be mainly dependent on two factors (aromaticity and hydrogen bonding, wherein aromaticity is the main contributor). For example, peptide **15** gave slightly higher stabilisation for c-Myc and c-Kit compared to peptide **11**, hinting to the fact that aromaticity (phenyl > thiophene) of the side chain might be the determining factor. However, very bulky side chains, such as in peptide **14**, which can both engage in hydrogen bonding and π-π-stacking interactions, gave relatively lower induced *Tm* values for more compact G4s, such as c-Myc, h-TELO and T95-2T. Notably, the non-aromatic Ala at the N-terminus (peptide **16**) also resulted in significant stabilisation of most G4s, as it might interact, similar to residue A15, by $CH_3$-π stacking with the outer tetrads and by H-bond donation.

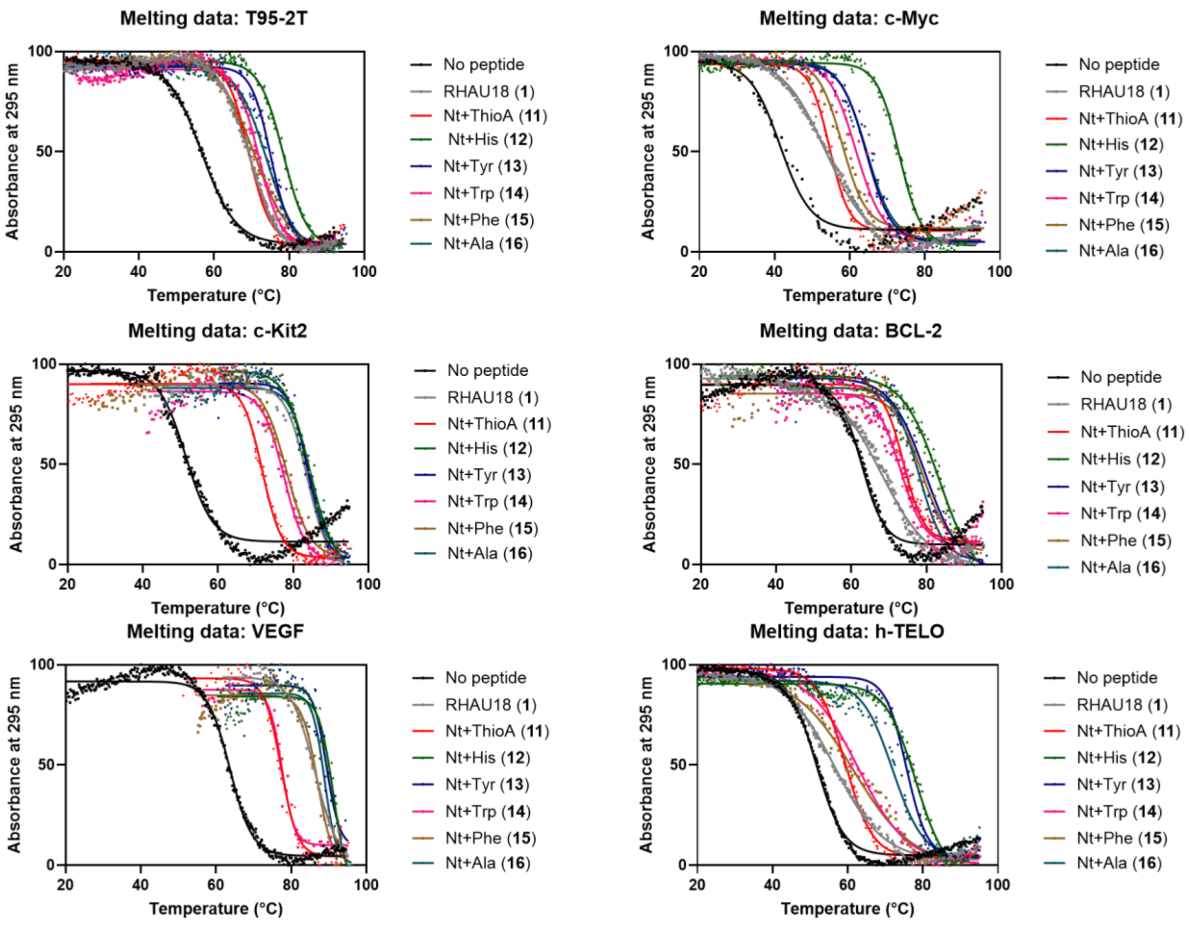


**Figure S42**. UV melting curves of all tested G4s with the N-terminal modified probes compared to RHAU18 and the G4 as such.

**Table S5**: Melting temperature values for the G4 sequences with and without the N-terminal modified peptides.

| G4 | $T_m$ (°C) | Used peptide | $\Delta T_m$ (°C) | G4 | $T_m$ (°C) | Used peptide | $\Delta T_m$ (°C) |
|---|---|---|---|---|---|---|---|
| T95-2T | 57.2 ± 0.2 | Nt+ThioA (**11**) | 12.3 ± 0.4 | BCL-2 | 62.9 ± 0.1 | Nt+ThioA (**11**) | 10.8 ± 0.4 |
| | | Nt+His (**12**) | 21.3 ± 0.4 | | | Nt+His (**12**) | 20.1 ± 0.7 |
| | | Nt+Tyr (**13**) | 17.7 ± 0.7 | | | Nt+Tyr (**13**) | 18.0 ± 0.9 |
| | | Nt+Trp (**14**) | 14.4 ± 1.5 | | | Nt+Trp (**14**) | 10.7 ± 1.8 |
| | | Nt+Phe (**15**) | 13.3 ± 0.9 | | | Nt+Phe (**15**) | 17.5 ± 1.8 |
| | | Nt+Ala (**16**) | 17.4 ± 0.8 | | | Nt+Ala (**16**) | 16.0 ± 0.9 |
| c-Myc | 41.0 ± 0.3 | Nt+ThioA (**11**) | 12.7 ± 1.1 | VEGF | 63.6 ± 0.1 | Nt+ThioA (**11**) | 14.2 ± 0.7 |
| | | Nt+His (**12**) | 32.2 ± 0.4 | | | Nt+His (**12**) | 27.2 ± 0.5 |
| | | Nt+Tyr (**13**) | 24.2 ± 1.5 | | | Nt+Tyr (**13**) | 27.3 ± 1.5 |
| | | Nt+Trp (**14**) | 20.0 ± 0.9 | | | Nt+Trp (**14**) | 13.7 ± 1.1 |
| | | Nt+Phe (**15**) | 16.5 ± 0.7 | | | Nt+Phe (**15**) | 24.1 ± 1.5 |
| | | Nt+Ala (**16**) | 22.9 ± 0.9 | | | Nt+Ala (**16**) | 27.6 ± 1.3 |
| c-Kit2 | 51.7 ± 0.3 | Nt+ThioA (**11**) | 21.5 ± 1.7 | h-TELO | 51.6 ± 0.1 | Nt+ThioA (**11**) | 8.7 ± 0.4 |
| | | Nt+His (**12**) | 33.1 ± 1.5 | | | Nt+His (**12**) | 26.6 ± 0.2 |
| | | Nt+Tyr (**13**) | 33.6 ± 1.5 | | | Nt+Tyr (**13**) | 24.3 ± 0.4 |
| | | Nt+Trp (**14**) | 26.1 ± 1.1 | | | Nt+Trp (**14**) | 11.2 ± 0.1 |
| | | Nt+Phe (**15**) | 26.7 ± 0.6 | | | Nt+Phe (**15**) | 11.5 ± 1.6 |
| | | Nt+Ala (**16**) | 32.4 ± 0.7 | | | Nt+Ala (**16**) | 20.9 ± 1.0 |

## 9. CD conformational shift experiments with N-terminal modified peptides

A 150 µL of 5 µM DNA solution was prepared in 20 mM Tris HCl pH 7.4 buffer containing 10 mM KCl (for hybrid h-TELO) or 25 mM NaCl (for anti-parallel h-TELO) or 25 mM KCl (for NOP56 and kit*). The solutions were pre-annealed either without or in presence of peptide by heating for 5 minutes at 95°C, followed by a cooling down period of 4 h. The CD signature was measured every day, for 2-4 days long, from 320 nm to 220 nm as wavelength range.

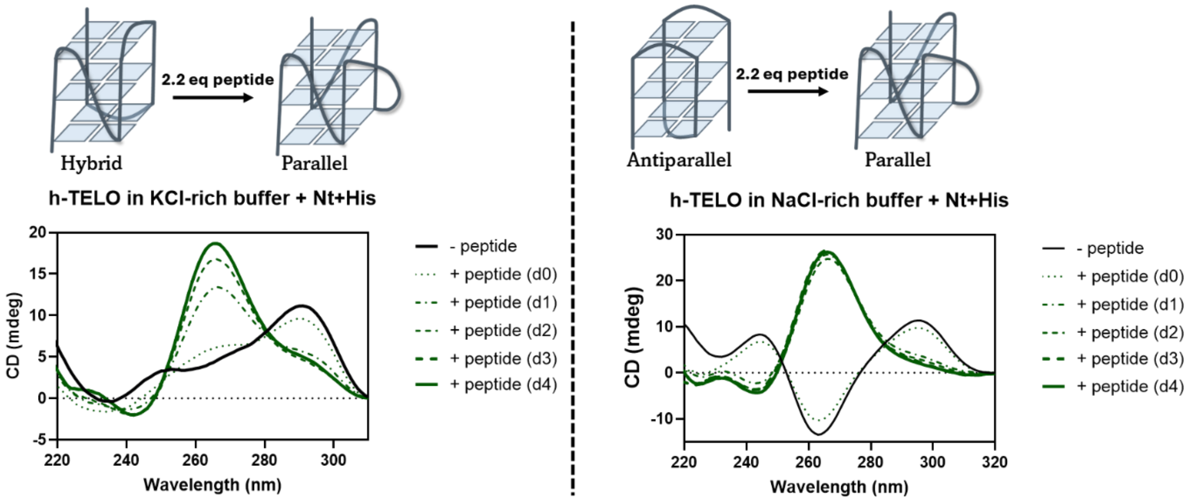


**Figure S43**. CD spectra of h-TELO in KCl-rich buffer (left) or in NaCl-rich buffer (right) to which Nt+His (**12**) was added and the kinetic refolding was followed for 4 days. The obtained spectrum was compared with the signature of the anti-parallel h-TELO as such (black line).

## 10. ITC experiments

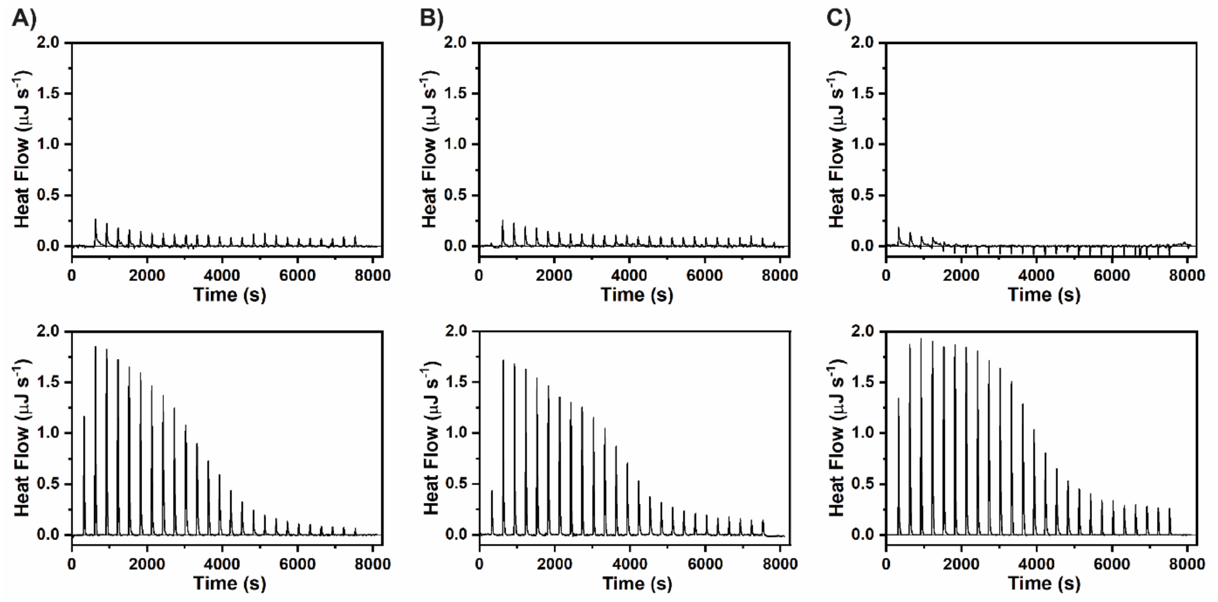


**Figure S44**. Raw ITC data for (top) control experiments and (bottom) c-Myc G4 titrations performed at 25 °C by injecting A) RHAU18 (**1**), B) Nt+FurA (**6**), and C) Nt+His (**12**) peptides into the buffer (5 mM $KH_2PO_4/K_2HPO_4$, pH 7.0, with 20 mM KCl) and into the G4 solution, respectively.

# 11. Structural analysis of the peptides upon c-Myc G4-binding

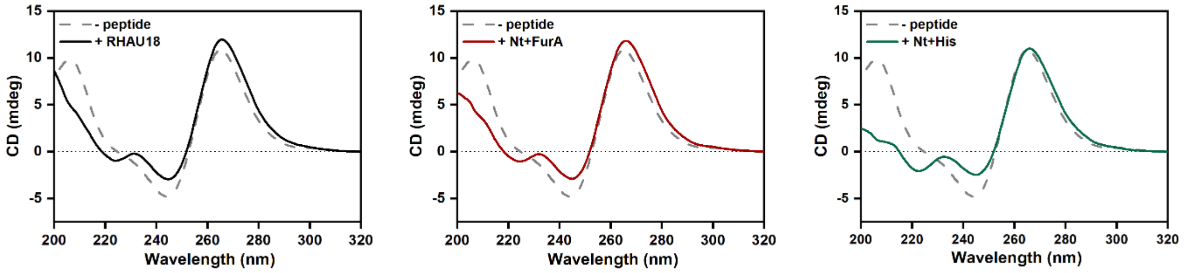


**Figure S45**. CD spectra of the c-Myc G4 (1.5 μM) recorded at 20 °C in 5 mM $KH_2PO_4/K_2HPO_4$ buffer (pH 7.0) containing 20 mM KCl, in the absence (grey dotted line) and presence (color-coded lines) of each peptide (2 molar equiv). The addition of RHAU18 (**1**), Nt+FurA (**6**) and Nt+His (**12**) did not induce significant changes in the characteristic CD signature of DNA.

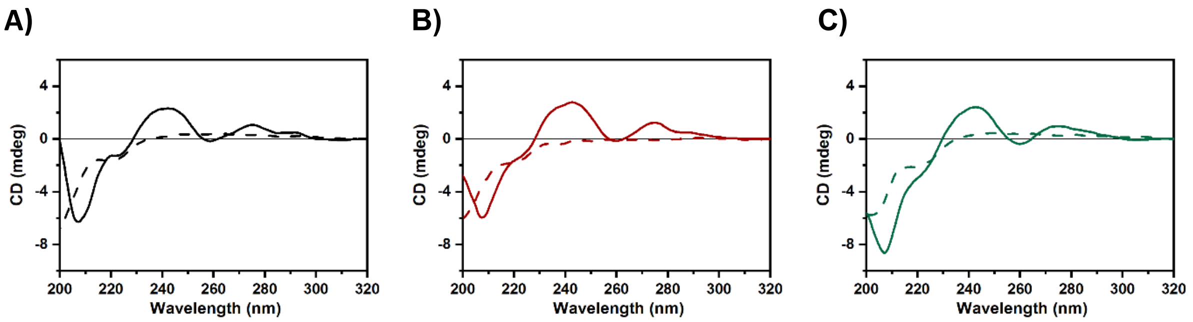


**Figure S46**. CD spectra of **A**) RHAU18 (**1**), **B**) Nt+FurA (**6**), and **C**) Nt+His (**12**) peptides (3 μM) recorded at 25 °C in 5 mM $KH_2PO_4/K_2HPO_4$ buffer (pH 7.0) containing 20 mM KCl, in the absence (dashed line) and presence (solid line) of c-Myc G4 (1.5 μM). Subtraction of the G4 signal from the spectrum of each G4/peptide complex was carried out to enable the detection of conformational changes in the peptide upon G4-binding.

**Table S6**: Secondary structure content estimation (in %) for RHAU18 (**1**), Nt+FurA (**6**) and Nt+His (**12**) in the absence and presence of the c-Myc G4 using BeStSel.

| | Helix | Antiparallel | Turn | Others |
|---|---|---|---|---|
| RHAU18 (**1**) | 2.1 | 28.6 | 19.6 | 49.6 |
| Nt+FurA (**6**) | 2.9 | 29.6 | 17.6 | 49.9 |
| Nt+His (**12**) | 4.9 | 22.2 | 19.5 | 53.5 |
| RHAU18 (**1**) + c-Myc G4 | 34.7 | 26.0 | 8.2 | 31.0 |
| Nt+FurA (**6**) + c-Myc G4 | 36.7 | 37.4 | 11.3 | 14.6 |
| Nt+His (**12**) + c-Myc G4 | 35.4 | 30.8 | 12.1 | 21.6 |

## 12. NMR experiments

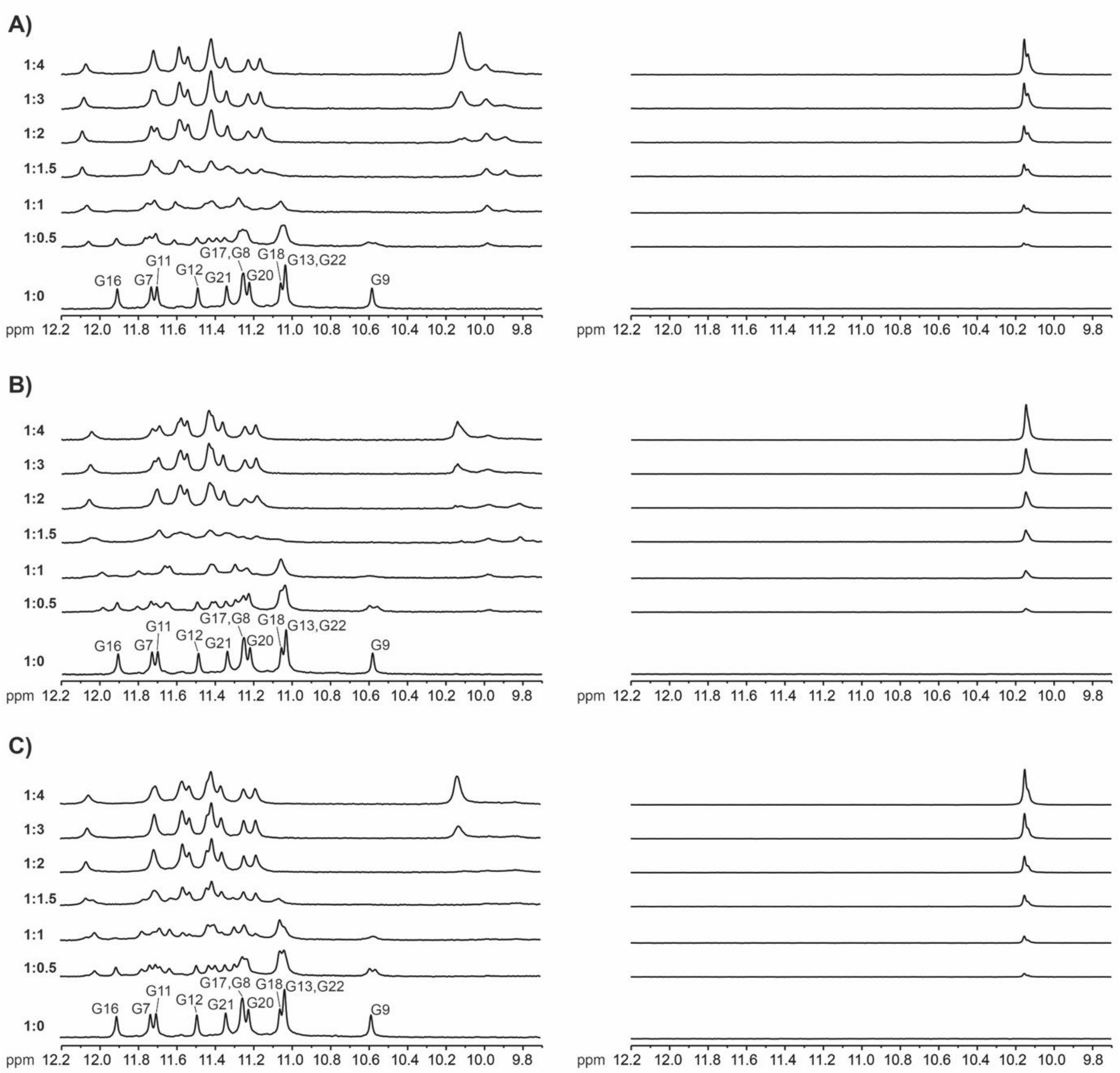


**Figure S47**. Imino proton region of the NMR spectra for the c-Myc G4 at 25°C, showing titrations with three peptides: A) RHAU18 (**1**), B) Nt+FurA (**6**), and C) Nt+His (**12**); the ratio between DNA and peptide equivalents is indicated to the left of each spectrum. (*Right*) Imino proton region of the NMR spectra for the peptides alone, recorded at the same concentrations used in the titrations. All samples were prepared in 5 mM $KH_2PO_4$/$K_2HPO_4$ buffer, pH 7.0, containing 20 mM KCl and 10% $D_2O$.

# 13. Appendix

## HPLC-UV stability check and HPLC-MS characterisation of peptides

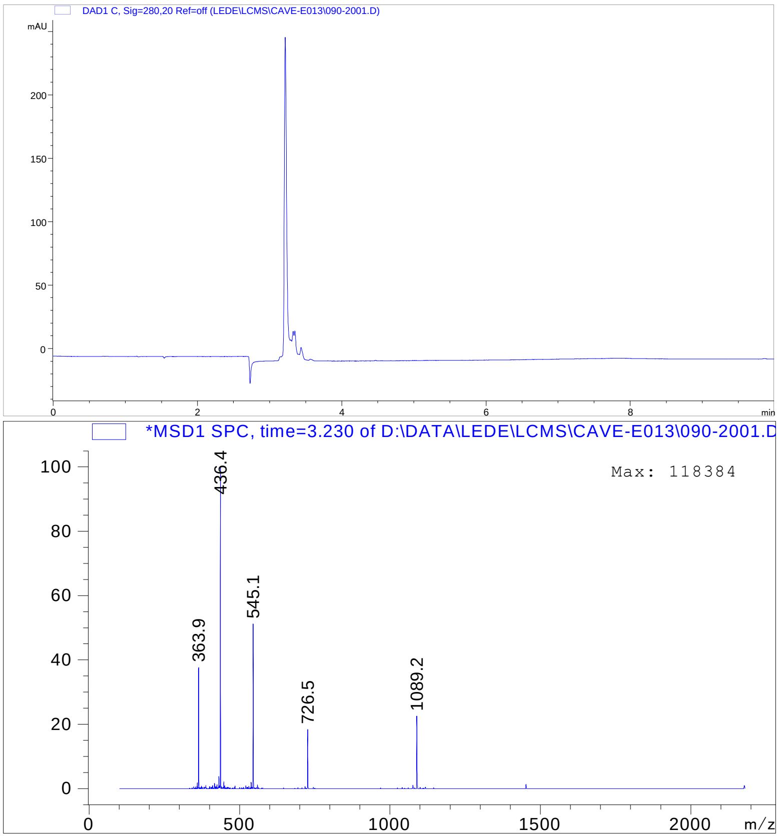


**Figure SA1**. HPLC-MS characterisation of purified RHAU18 (**1**). HPLC-UV trace at 280 nm (top) and MS spectrum of the corresponding peak (bottom). Calcd. MW: 2176.

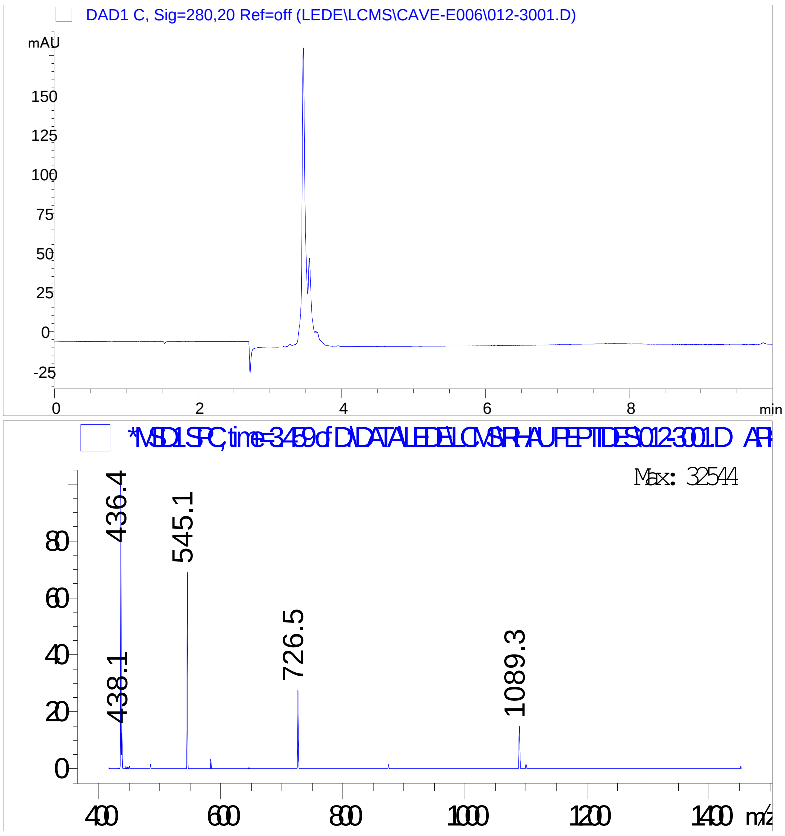


**Figure SA2**. HPLC-MS characterisation of purified H1:FurA (**7**). HPLC-UV trace at 280 nm (top) and MS spectrum of the corresponding peak (bottom). Calcd. MW: 2176.

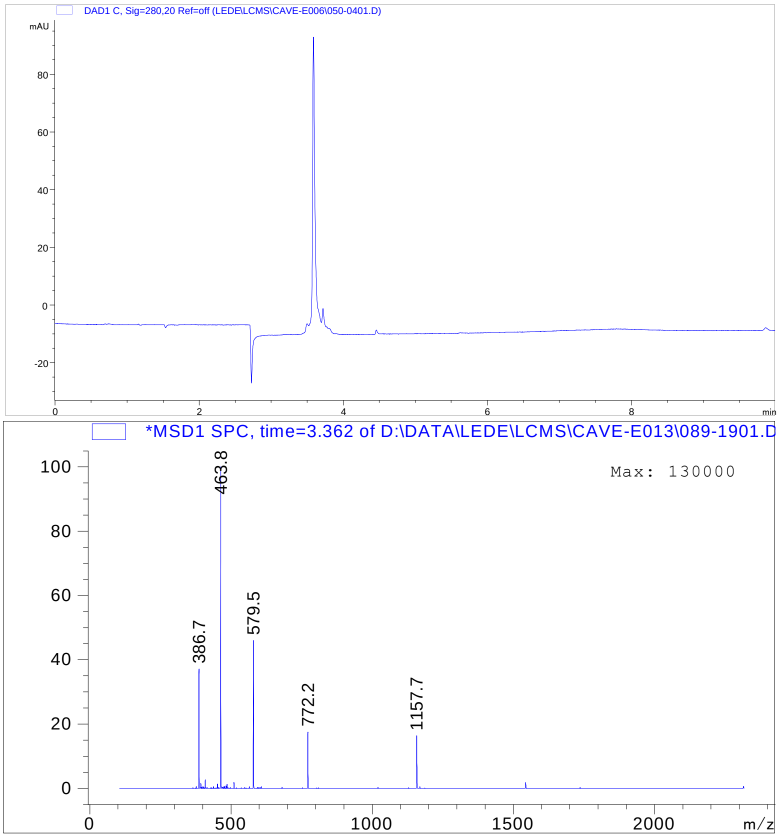


**Figure SA3**. HPLC-MS characterisation of purified Nt+FurA (**6**). HPLC-UV trace at 280 nm (top) and MS spectrum of the corresponding peak (bottom). Calcd. MW: 2316.

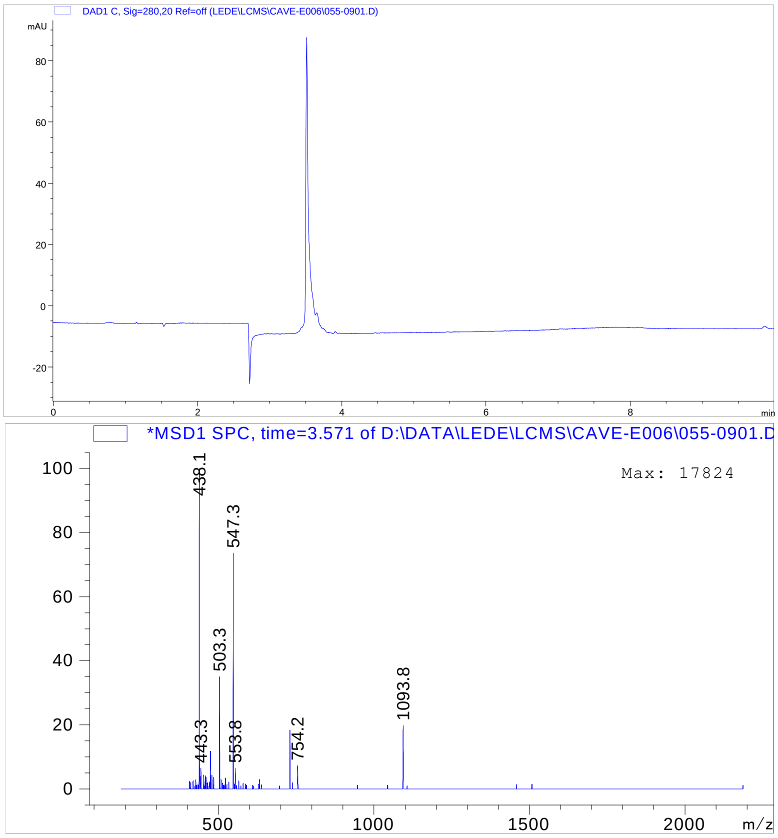


**Figure SA4**. HPLC-MS characterisation of purified K6:FurA (**8**). HPLC-UV trace at 280 nm (top) and MS spectrum of the corresponding peak (bottom). Calcd. MW: 2185.

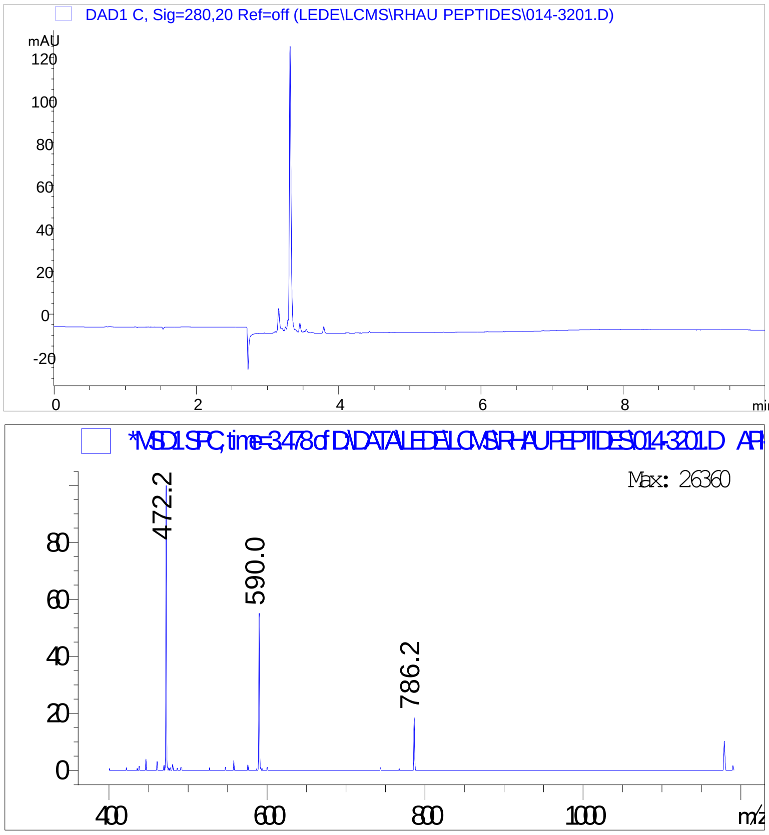


**Figure SA5**. HPLC-MS characterisation of purified K6+FurA (**9**). HPLC-UV trace at 280 nm (top) and MS spectrum of the corresponding peak (bottom). Calcd. MW: 2356.

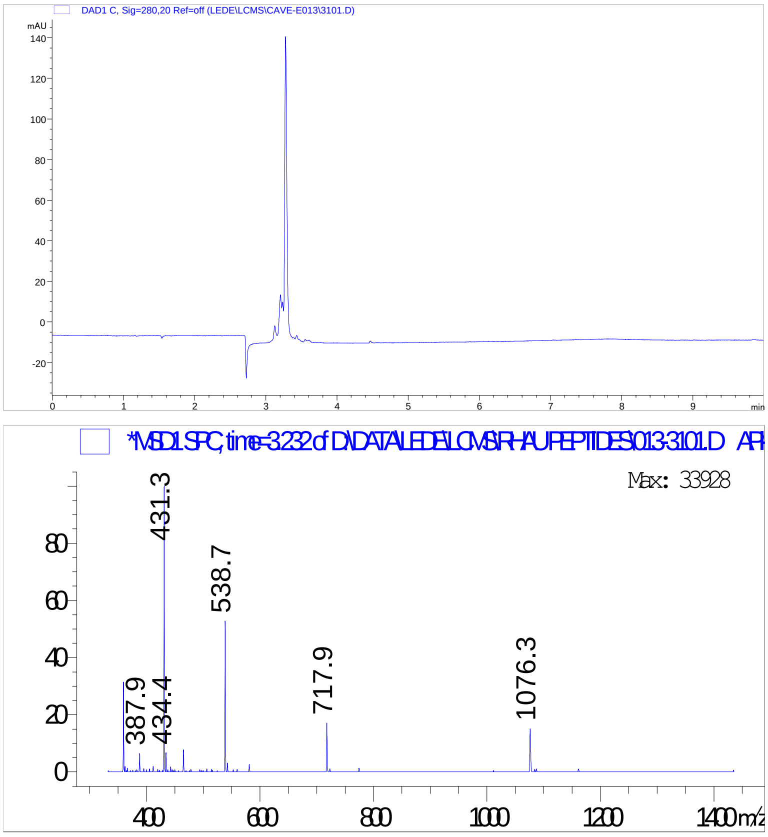


**Figure SA6**. HPLC-MS characterisation of purified Y14:FurA (**10**). HPLC-UV trace at 280 nm (top) and MS spectrum of the corresponding peak (bottom). Calcd. MW: 2151.

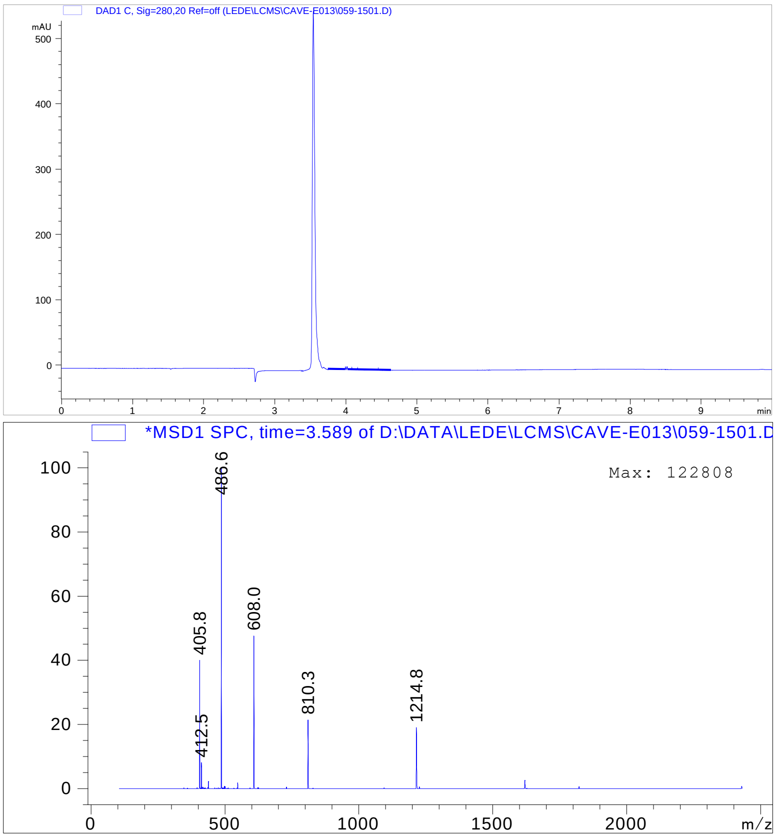


**Figure SA7**. HPLC-MS characterisation of purified Nt+BPA (**2**). HPLC-UV trace at 280 nm (top) and MS spectrum of the corresponding peak (bottom). Calcd. MW: 2428.

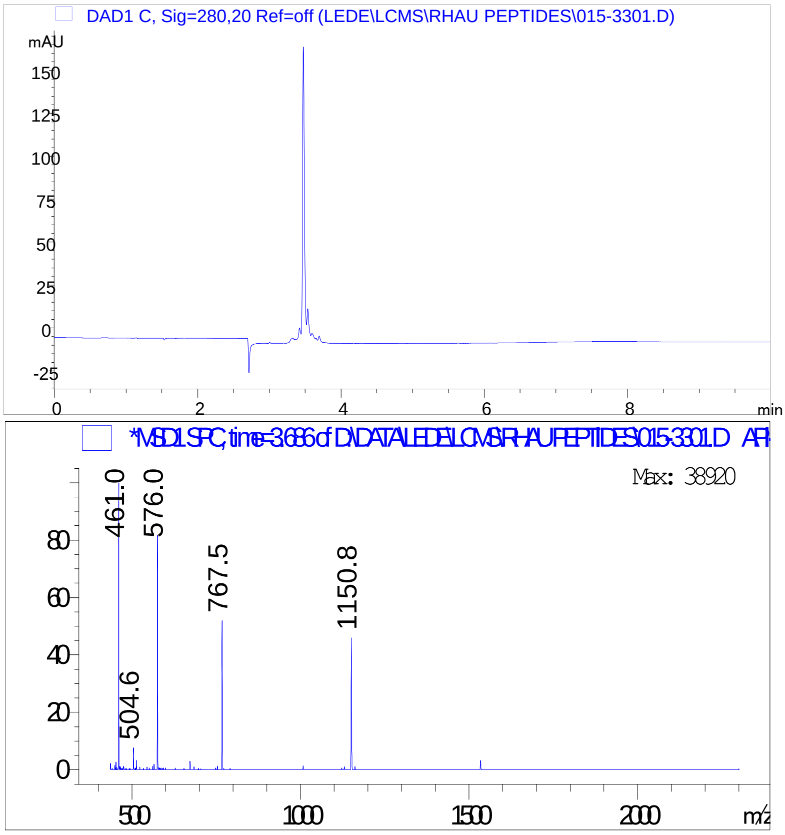


**Figure SA8**. HPLC-MS characterisation of purified K6:BPA (**3**). HPLC-UV trace at 280 nm (top) and MS spectrum of the corresponding peak (bottom). Calcd. MW: 2300.

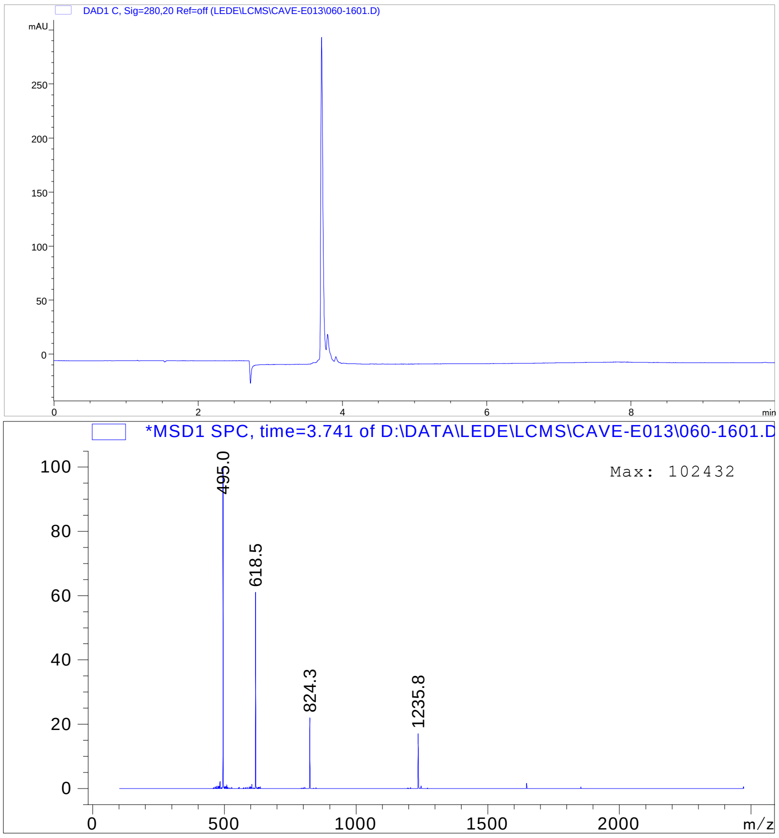


**Figure SA9**. HPLC-MS characterisation of purified K6+BPA (**4**). HPLC-UV trace at 280 nm (top) and MS spectrum of the corresponding peak (bottom). Calcd. MW: 2470.

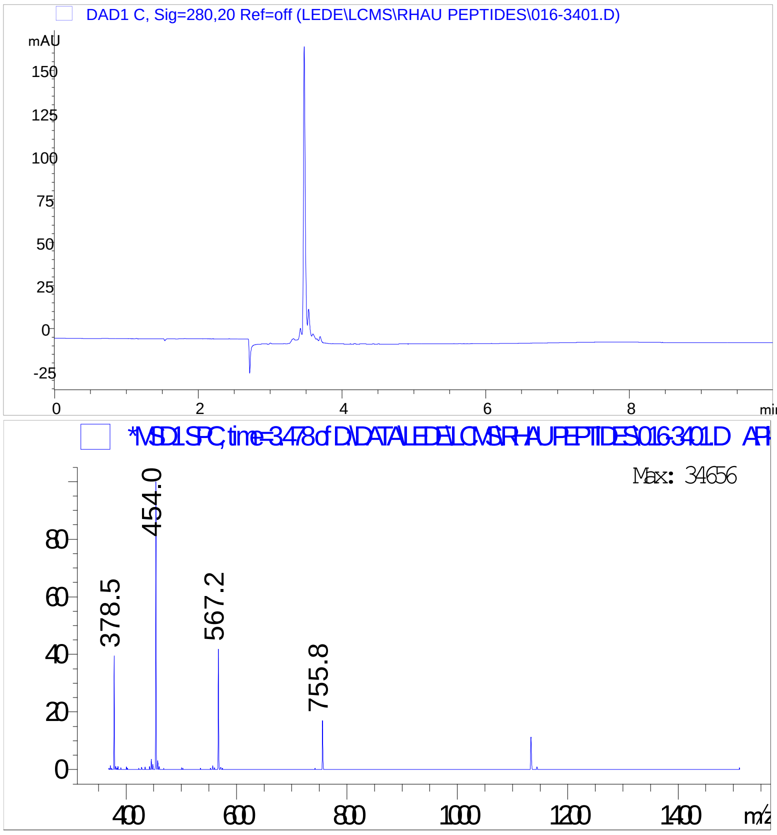


**Figure SA10**. HPLC-MS characterisation of purified Y14:BPA (**5**). HPLC-UV trace at 280 nm (top) and MS spectrum of the corresponding peak (bottom). Calcd. MW: 2265.

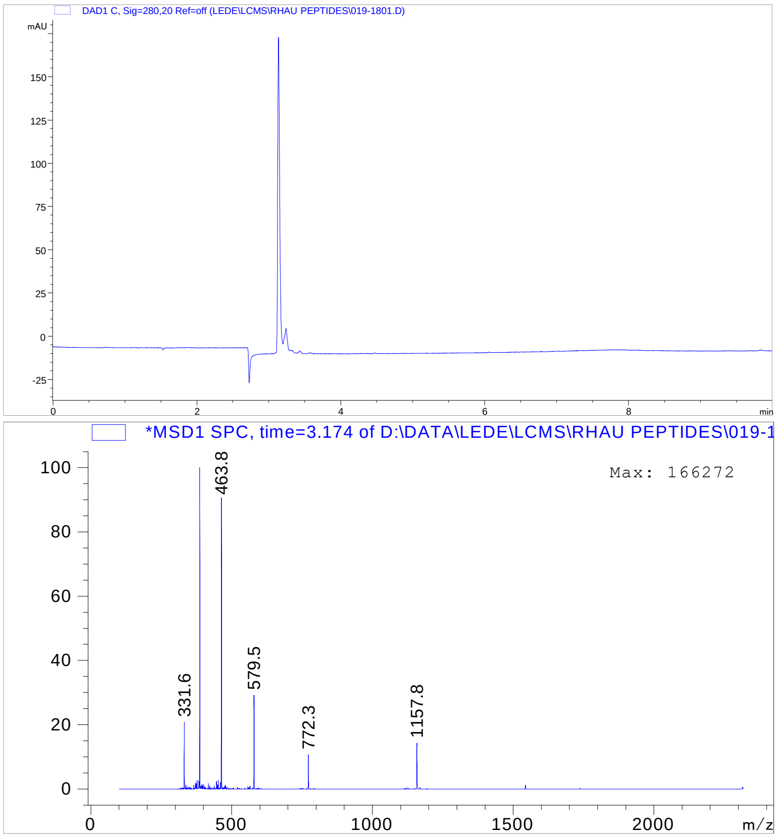


**Figure SA11**. HPLC-MS characterisation of purified Nt+His (**12**). HPLC-UV trace at 280 nm (top) and MS spectrum of the corresponding peak (bottom). Calcd. MW: 2316.

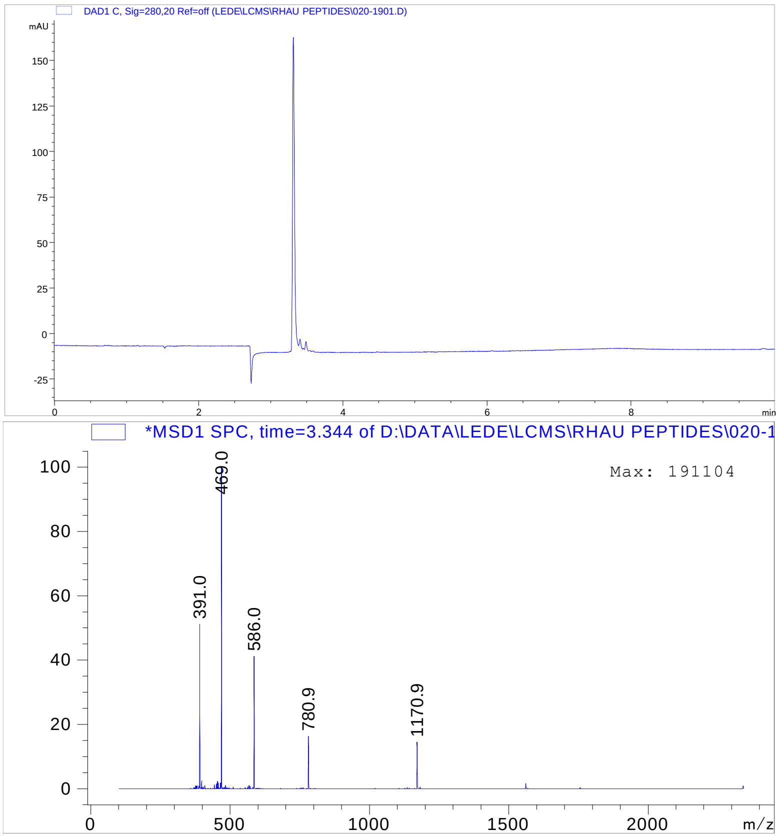


**Figure SA12**. HPLC-MS characterisation of purified Nt+Tyr (**13**). HPLC-UV trace at 280 nm (top) and MS spectrum of the corresponding peak (bottom). Calcd. MW: 2342.

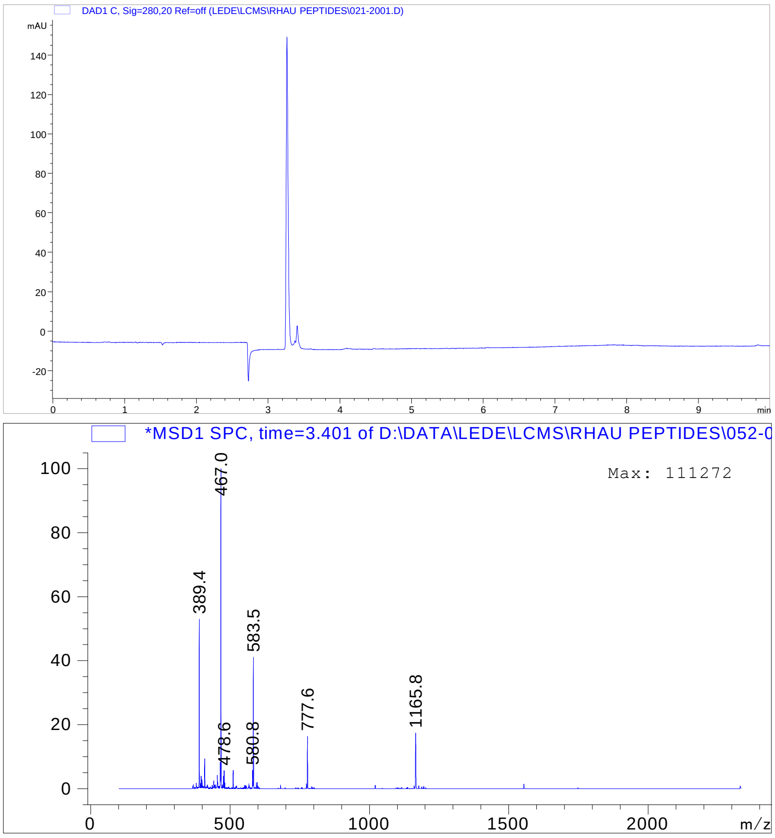


**Figure SA13**. HPLC-MS characterisation of purified Nt+ThioA (**11**). HPLC-UV trace at 280 nm (top) and MS spectrum of the corresponding peak (bottom). Calcd. MW: 2331.

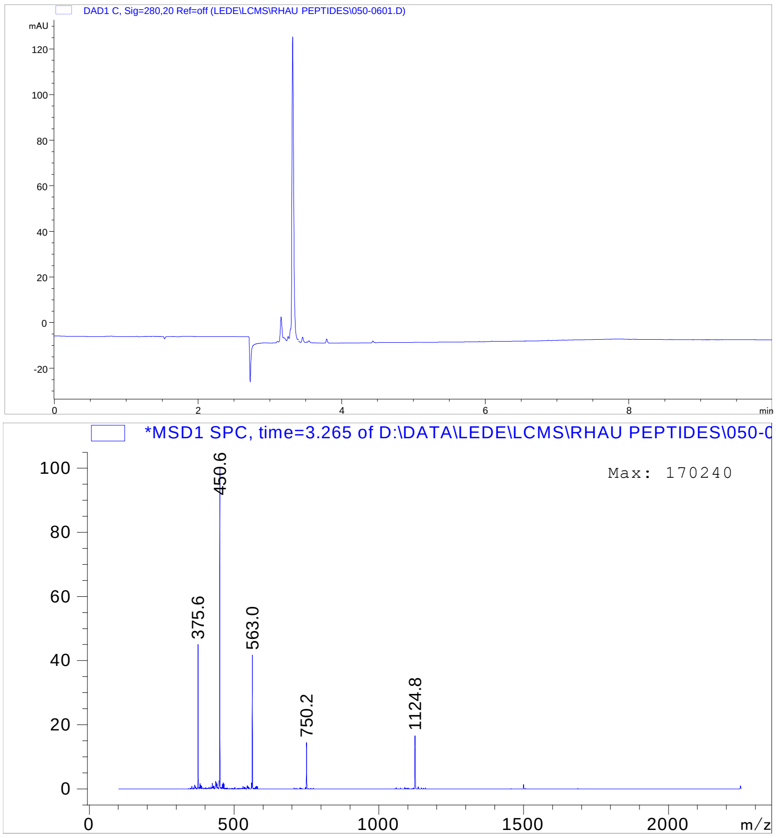


**Figure SA14**. HPLC-MS characterisation of purified Nt+Ala (**16**). HPLC-UV trace at 280 nm (top) and MS spectrum of the corresponding peak (bottom). Calcd. MW: 2249.

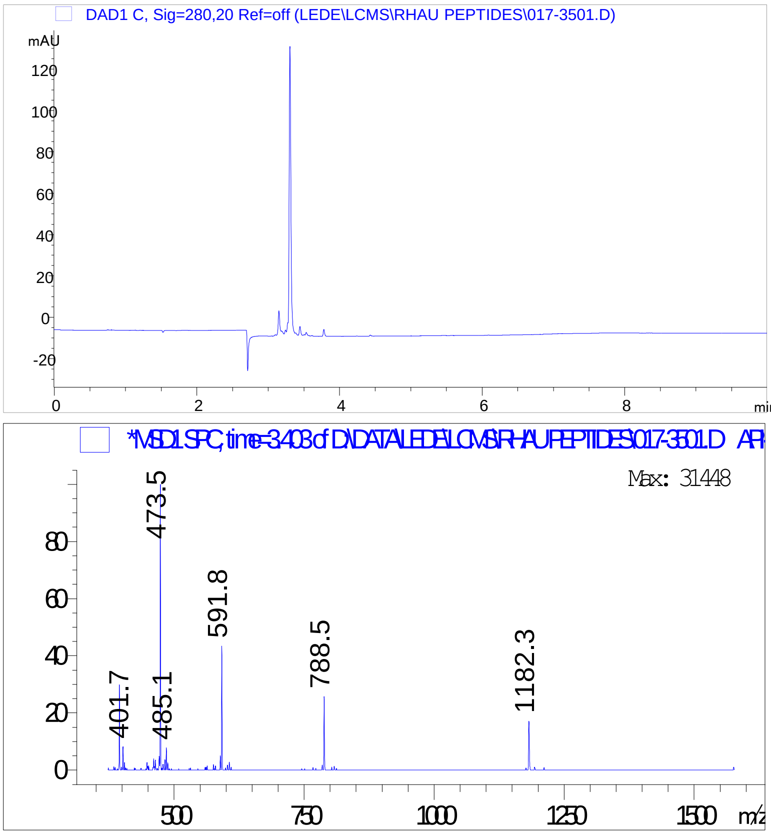


**Figure SA15**. HPLC-MS characterisation of purified Nt+Trp (**14**). HPLC-UV trace at 280 nm (top) and MS spectrum of the corresponding peak (bottom). Calcd. MW: 2363.

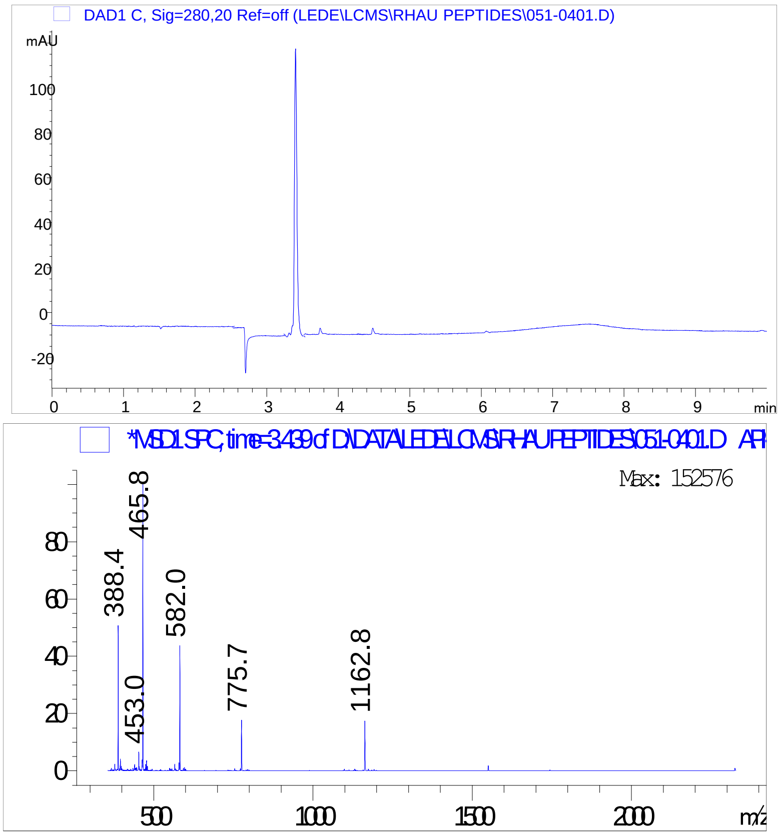


***Figure SA16***. *HPLC-MS characterisation of purified Nt+Phe (**15**). HPLC-UV trace at 280 nm (top) and MS spectrum of the corresponding peak (bottom). Calcd. MW: 2325.*
